# Macrocyclic molecular engineering enables high-energy and durable organic electrode for lithium-ion batteries

**DOI:** 10.1038/s41467-026-76984-5

**Published:** 2026-08-29

**Authors:** Huiling Peng, Yaheng Geng, Yu Han, Jin Xiao, Zehao Yu, Zichao Yan, Zhiqiang Zhu

**Affiliations:** 1 State Key Laboratory of Chemo and Biosensing, College of Chemistry and Chemical Engineering, Hunan University, Changsha, China; 2 School of Science, Hunan University of Technology, Zhuzhou, China

**Keywords:** Batteries, Batteries, Batteries

## Abstract

Organic electrode materials are promising candidates for lithium-ion batteries, but their practical adoption is constrained by the persistent trade-off between capacity and cycling stability. While small-molecule organic compounds deliver high capacities yet suffer from dissolution, covalent organic frameworks enhance stability at the expense of active-site accessibility. Here, we introduce a macrocyclic molecular design synergizing framework-like robustness with small-molecule redox efficiency. The designed hexagonal monocyclic benzoquinone integrates a π-conjugated core with densely packed electroactive moieties (carbonyl, imine, and hydroxyl), enabling a high theoretical capacity of 812 mAh g^−1^. As a positive electrode, it achieves near-theoretical capacity (806 mAh g^−1^ at 0.05 A g^−1^) with a high specific energy of 1507 Wh kg_HPBQ_^−1^, while maintaining 81% capacity after 1000 cycles at 5 A g^−1^. Mechanistic studies explore a potential 36-electron transfer mechanism per macrocyclic unit during initial discharge, followed by irreversible hydroxyl-to-carbonyl conversion upon charging, which generates a phenanthrenequinone-based macrocycle capable of reversible 48-electron transfer per macrocyclic unit in subsequent cycles. This molecular architecture has the potential to overcome the longstanding capacity-stability trade-off in organic electrodes.

## Abstract (web summary)

Organic electrode materials face a persistent capacity–stability trade-off. Here, authors design a macrocycle combining covalent organic framework-like robustness with small-molecule redox efficiency, which renders a capacity of 806 mAh g^−1^ at 50 mA g^−1^ and retains 81% capacity after 1000 cycles at 5 A g^−1^.

## Introduction

The rapid growth of electric vehicles and grid-scale energy storage systems demands lithium-ion batteries (LIBs) with high energy density, long cycle life, and minimal environmental footprint^1–5^. However, traditional transition-metal-based inorganic positive electrodes, such as layered oxides and polyanionic compounds, are constrained by their intrinsic theoretical capacity limits (typically ≤200 mAh g^−1^) and reliance on scarce metal resources, posing significant challenges to sustainable energy storage development^6–9^. Organic electrode materials (OEMs), composed of earth-abundant elemental constituents (C, H, O, N, S, etc), emerge as a disruptive paradigm owing to their molecularly tunable redox properties, potential for green synthesis, and inherent sustainability^10–15^. Despite these advantages, their practical adoption remains hindered by critical barriers, such as severe dissolution in liquid electrolytes, inefficient utilization of active sites, and poor electronic/ionic conductivity, resulting in rapid capacity decay and energy densities far below theoretical predictions.

Numerous organic compounds, such as conducting polymers^16,17^, organosulfur compounds^18^, organic radicals^19^, carbonyl compounds^20–22^, imine compounds^23^, and azo compounds^24^, have been explored as positive electrode materials for LIBs. Among these, small-molecule organic carbonyl compounds (SMOCCs) are particularly attractive as high-energy positive electrode candidates due to their potential for achieving high theoretical capacities through dense integration of redox-active sites^25–27^. In addition, their compact molecular structure enables full exposure of active sites, ensuring near-unity utilization efficiency^28–31^. For this reason, certain SMOCCs demonstrate specific capacities exceeding 500 mAh g^−1^ (see refs. ^13, 32, 33^). However, their low molecular weights and insufficient intermolecular forces trigger significant dissolution in conventional electrolytes, causing rapid capacity fading^28,34,35^. Although several rigid macrocycles have been designed to partially address the dissolution issue, the incorporation of non-active structural units inevitably compromises the theoretical capacity^36–38^. To date, only two SMOCCs (C_6_O_6_ and C_5_O_5_) have achieved capacities higher than 600 mAh g^−1^, yet both exhibit inadequate cycle stability (< 100 cycles) even with specialized electrolytes (i.e., ionic liquids or quasi-solid systems)^27,33^. In addition, achieving the high specific capacity of C_6_O_6_ required an elevated operating temperature of 70 °C. Moreover, their inherent high chemical reactivity mandates inert synthesis/storage conditions, further compromising practical viability. Therefore, designing high-capacity SMOCCs (>600 mAh g^−1^) with stable cycle stability remains a big challenge.

Covalent organic frameworks (COFs), an emerging class of crystalline porous polymers, represent a structural innovation to mitigate dissolution due to their good chemical and thermal stability derived from robust covalent bonding^30,39^. Nevertheless, their rigid frameworks usually incorporate non-redox-active linkers, diminishing theoretical capacities^40^. While dense-redox-group COFs have been designed to overcome this limitation, their deeply buried active sites within stacked layers restrict ion accessibility, yielding low utilization rates and consequently unsatisfactory practical capacities^41–43^. For example, HAPT-COF features a theoretical capacity of 788 mAh g^−1^, but its practical capacity at 0.1 A g^−1^ was merely 192 mAh g^−1^. Integrating HAPT-COF with reduced graphene oxide to form a nanocomposite enhances electronic conductivity and ionic transport, thus increasing the initial capacity to 558 mAh g^−1^ (70% active-site utilization)^44^. Nevertheless, a portion of active sites still remains inaccessible. This trade-off between solubility suppression and redox accessibility has persisted as a fundamental barrier for organic positive electrodes to achieving high capacity and long-term cyclability simultaneously^45^.

**Fig. 1:**
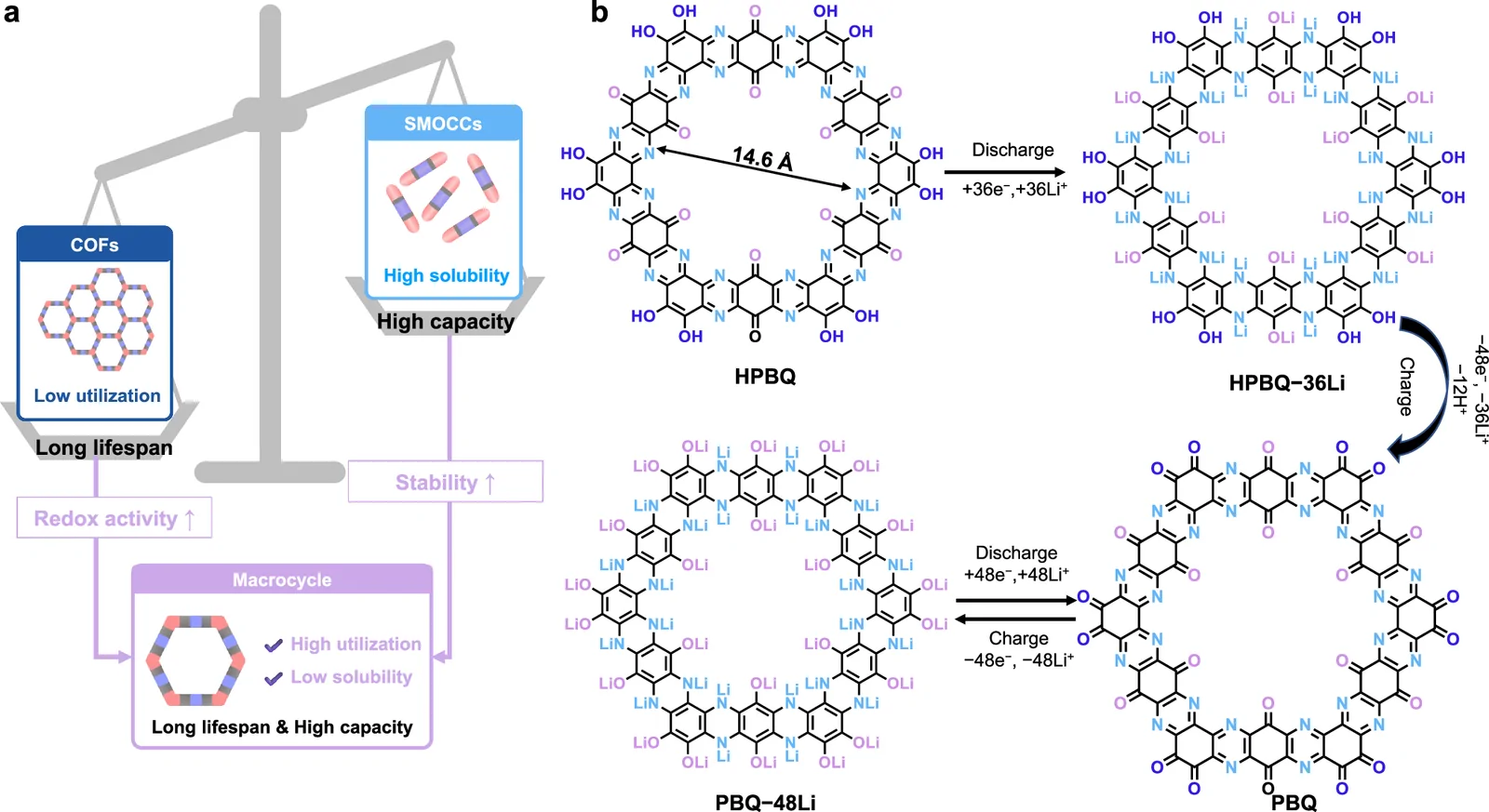
Molecular design of the macrocyclic system. **a** Design concept illustrating how the macrocyclic architecture integrates the high specific capacity of SMOCCs with the structural stability of COFs. Electrochemically active sites are denoted in pink and blue; aromatic rings and the conjugated framework are highlighted in grey. **b** Proposed reaction mechanism schematic of HPBQ.

Herein, we report a macrocyclic molecular engineering strategy that reconciles the structural stability of COFs with the redox efficiency of SMOCCs (Fig. 1a). Our designed molecule, hexagonal monocyclic benzoquinone (HPBQ, Fig. 1b), integrates a π-conjugated benzoquinone core with strategically positioned carbonyl (C = O), hydroxy (–OH), and imine (C = N) groups. This architecture synergistic achieves three objectives: (1) dense integration of electroactive moieties enables high theoretical capacity (812 mAh g^−1^), (2) small-molecule dimensions ensure full active site accessibility, and (3) extended π-conjugation enhances structural stability and suppresses dissolution. Remarkably, HPBQ not only achieved near-theoretical capacity delivery (806 mAh g^−1^ at 0.05 A g^−1^) with an average operating potential of 1.87 V (vs. Li^+^/Li), yielding a high specific energy of 1507 Wh kg^−1^_HPBQ_, but also demonstrated satisfactory cycling stability, maintaining 81% capacity retention after 1000 cycles at 5 A g^−1^. This macrocyclic molecular design shows the potential to overcome conventional capacity-stability trade-offs in organic electrodes, offering a blueprint toward sustainable high-energy batteries.

## Results

### Molecular design principles and mechanism prediction

To simultaneously achieve high capacity and long-term cyclability, we rationally designed a hyper conjugated macrocyclic molecule, HPBQ, which features a rigid π-conjugated hexagonal monocyclic framework integrating periodic pyrazine (C = N), carbonyl (C = O), and hydroxyl (–OH) moieties. Its optimized molecular structure exhibits a large inner ring diameter of 14.6 Å that facilitates active-sites exposure and ion transport. As demonstrated in previous reports^46,47^, conjugated organic molecules bearing hydroxyl groups undergo irreversible conversion to carbonyl-based analogues during initial electrochemical cycling, thus increasing redox-active sites as well as charge storage capacity. The released H^+^ could react with anions from the electrolyte, thereby avoiding the detrimental formation of water within the battery system^46,47^. Aligning with this mechanism, HPBQ is expected to deliver 36 redox-active sites (i.e., the total number of C = O and C = N) per macrocyclic unit during the initial discharging process, yielding a theoretical capacity of 604 mAh g^−1^. Upon charging, the 12 hydroxyl groups in HPBQ are irreversibly oxidized to carbonyl groups, forming PBQ. This structural evolution enables reversible 48-electron transfer (i.e., the total number of C = O and C = N) per macrocyclic unit in subsequent cycles, offering a high theoretical capacity of 812 mAh g^−1^. Moreover, HPBQ’s molecular-scale dimensions ensure full active-site accessibility, while its extended π-conjugation suppresses dissolution, thereby resolving the critical capacity-stability trade-off for organic electrodes.

### Material characterizations

**Fig. 2:**
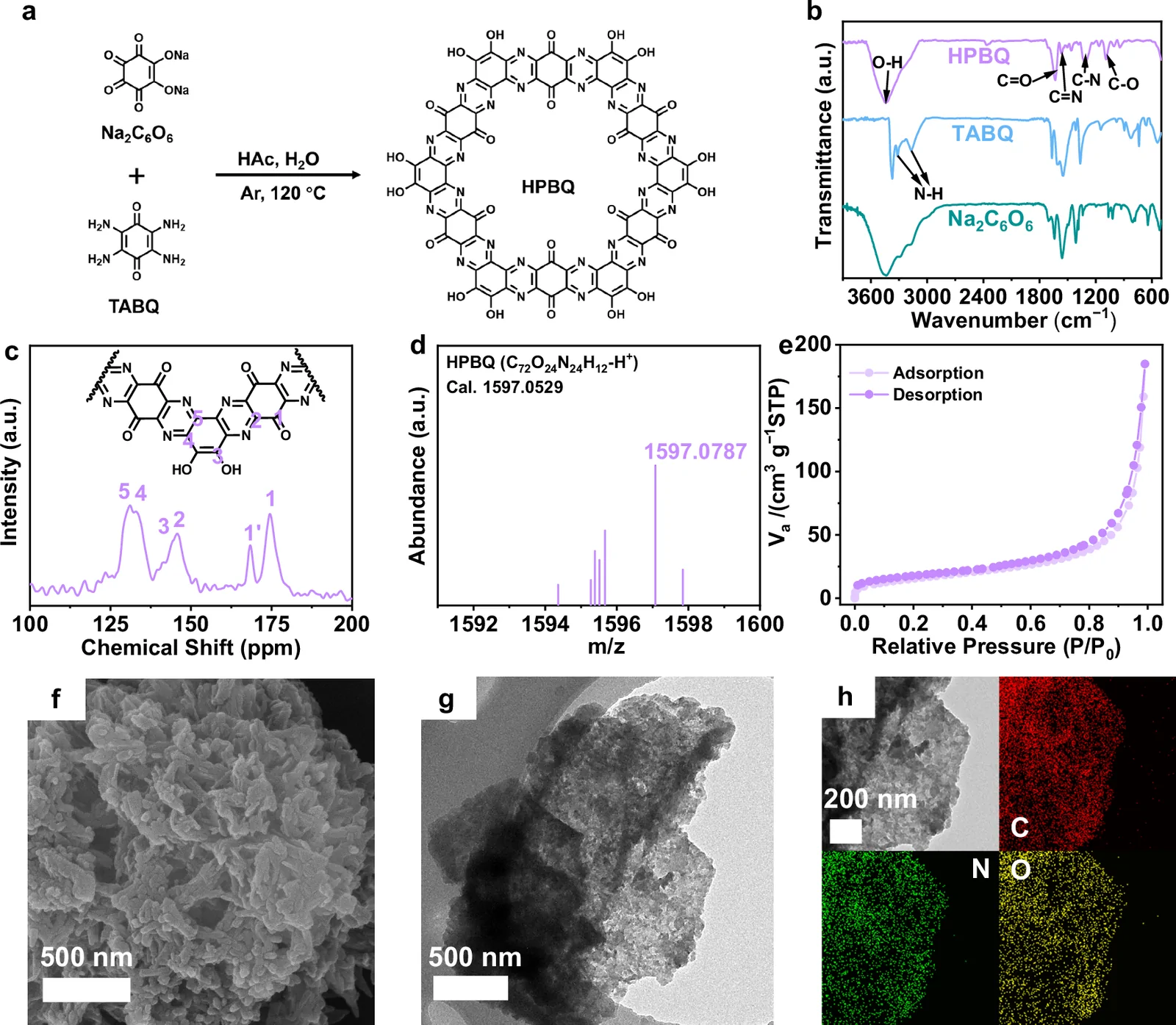
Synthesis and characterization of HPBQ. **a** Scheme for the synthesis of HPBQ. **b** FTIR spectra of HPBQ and precursor compounds. **c** ^13^C solid-state NMR spectrum, **d** liquid chromatography high-resolution mass spectrometry (LC–HRMS), **e** N_2_ adsorption/desorption isotherms, **f** SEM image, **g** TEM image, and **h** EDS elemental mapping of HPBQ.

HPBQ was prepared via a condensation reaction between 2,3,5,6-tetraamino-1,4-benzoquinone (TABQ, Fig. 2a and Supplementary Figs. S1 and S2) and disodium rhodizonate (Na_2_C_6_O_6_)^48^. Comprehensive spectroscopic characterization confirmed the successful formation of the target structure. Fourier transform infrared (FTIR) spectroscopy (Fig. 2b) reveals the complete disappearance of primary diamine N-H stretching bands at 3297 and 3164 cm^−1^ relative to precursor compounds, indicating full consumption of amino groups. Furthermore, HPBQ exhibited characteristic peaks for hydroxy (–OH, 3442 cm^−1^), carbonyl (C = O, 1619 cm^−1^), and C–O bond bending vibration (1093 cm^−1^), alongside new peaks corresponding to the C = N (1555 cm^−1^) and C–N bonds (1308 cm^−1^), consistent with the proposed pyrazine-quinone conjugated framework^25^. This structural assignment was corroborated by Raman spectroscopy (Supplementary Fig. S3), which showed distinct scattering bands for –OH (3352 cm^−1^), C = N (1570 cm^−1^), C–N (1388 cm^−1^), and C = O (1628 cm^−1^). X-ray photoelectron spectroscopy (XPS) elucidates the chemical bonding environments of HPBQ. The C *1 s* spectrum (Supplementary Fig. S4a) exhibits three peaks at 288.3 (C = O), 286.4 (C = N), and 284.8 eV (C = C), while the N *1 s* spectrum (Supplementary Fig. S4b) shows two peaks at 400.1 (C = N) and 398.8 eV (C–N), consistent with the HPBQ framework. Additionally, the O *1 s* spectrum shows two peaks at 531.6 and 533.4 eV, respectively representing O = C and O–C groups within the proposed molecular structure (Supplementary Fig. S4c).

Solid-state ^13^C NMR was performed to further elucidate the structure of HPBQ^49^. As shown in Fig. 2c, the characteristic signals of the precursors, including the carbonyl peak of Na_2_C_6_O_6_ at 171.12 ppm and the C–NH_2_ peak of TBAQ at 121.45 ppm, were completely absent (Supplementary Fig. S5). In the spectrum of HPBQ, resonances corresponding to the carbonyl carbon (peak 1), pyrazine carbons (peak 2, 4, 5), and hydroxyl-bearing carbon (peak 3) can be identified, which is consistent with the carbon skeleton of HPBQ. Given that no other carbonyl groups are present in the structure, peak 1′ may originate from terminal carbonyl groups resulting from incomplete cyclization^50,51^. Elemental analysis agrees well with the theoretical formula on a stoichiometric basis (Supplementary Table S1). It is worth noting that occasionally slightly lower carbon content was observed, likely due to the presence of a small fraction of uncyclized species. Liquid chromatography-high resolution mass spectrometry (LC–HRMS, Fig. 2d) displays a signal at *m/z* = 1597.0787 ([M–H]^+^), matching the calculated mass of HPBQ (*M* = 1596.0529). Collectively, these spectroscopic data confirm the successful synthesis of HPBQ, despite the presence of trace amounts of uncyclized and oxidized byproducts are difficult to remove completely.

The physicochemical properties of HPBQ were systematically characterized. N_2_ adsorption-desorption measurements reveals that the Brunauer-Emmett-Teller (BET) specific surface area of HPBQ was 70.56 m^2^ g^−1^ (Fig. 2e). Pore size and volume distribution analysis reveals that HPBQ possesses a hierarchically porous structure (Supplementary Fig. S6)^52^. Scanning electron microscopy (SEM) image reveal that HPBQ comprises interconnected nanoparticles (Fig. 2f), while transmission electron microscopy (TEM) analysis confirms the presence of loosely packed, porous aggregates (Fig. 2g), which further facilitate redox-active sites accessibility^53,54^. Corresponding elemental mapping confirms the homogeneous distribution of carbon (C), nitrogen (N), and oxygen (O) throughout the HPBQ structure (Fig. 2h).

The crystallinity of HPBQ was investigated by powder X-ray diffraction (PXRD) and high-resolution transmission electron microscopy (HRTEM). The PXRD pattern (Supplementary Fig. S7 and Table S2) exhibits a strong diffraction peak at 28.3°, corresponding to a regular interlayer distance of approximately 3.13 Å, a clear signature of π–π stacking between the conjugated aromatic systems^50^. Unfortunately, under the current experimental conditions, we were unable to obtain single crystals suitable for detailed structural analysis by single-crystal X-ray diffraction. To gain further insight into the packing arrangement of HPBQ, its PXRD pattern was compared with the simulated patterns of three stacking modes (AA, AB, and ABC) of a structurally analogous BQ-COF (Supplementary Fig. S8 and Supplementary Data 1). The major diffraction peaks of HPBQ at 14.9°, 18.2°, 20.0°, 28.3°, and 30.1° are more similar with the simulated pattern of the AB stacking model. The HRTEM image (Supplementary Fig. S9) further confirms the moderate crystallinity of HPBQ, where resolved lattice fringes with a spacing of 0.34 nm are observed. This spacing agrees well with the interlayer distance determined by PXRD. In addition, direct current (DC) polarization measurement (Supplementary Fig. S10) demonstrated that HPBQ exhibits good electronic conductivity (7.03 × 10^−7^ S cm^−1^), which provides the possibility for HPBQ to achieve high active site utilization^25,30,34^. The large π-conjugation of HPBQ contributes its insolubility in common organic solvents (Supplementary Fig. S11), which ensures structural stability during electrochemical cycling. Thermogravimetric analysis (TGA) revealed that HPBQ is thermally stable up to 359 °C (Supplementary Fig. S12).

### Electrochemical performance

The electrochemical performance of HPBQ as a positive electrode material for LIBs has been comprehensively investigated. For comparison, two structurally analogous compounds, including a small-molecule quinone derivative (QPT, see synthesis details in Supplementary Fig. S13 and material characterizations in Supplementary Figs. S14–S16 and Table S3) and a COF material (BQ-COF, see synthesis details in Supplementary Fig. S17 and material characterizations in Supplementary Figs. S18–S20 and Table S4), were synthesized and investigated. Theoretical capacities of QPT and BQ-COF are 565 and 773 mAh g^−1^, respectively. Systematic optimization of key parameters for the electrochemical testing of HPBQ was conducted, including the potential window (Supplementary Fig. S21), conductive additive (Supplementary Figs. S22 and S23), and electrolyte (Supplementary Fig. S24). The optimized conditions were a potential window of 3.9–1.0 V, a conductive additive composed of graphene oxide and carbon nanotube (1:1 mass ratio, 30% content in the electrode), and an electrolyte of 2 M LiTFSI in 1,2-dimethoxyethane/1,3-dioxolane (DOL/DME, 1:1 by vol.).

**Fig. 3:**
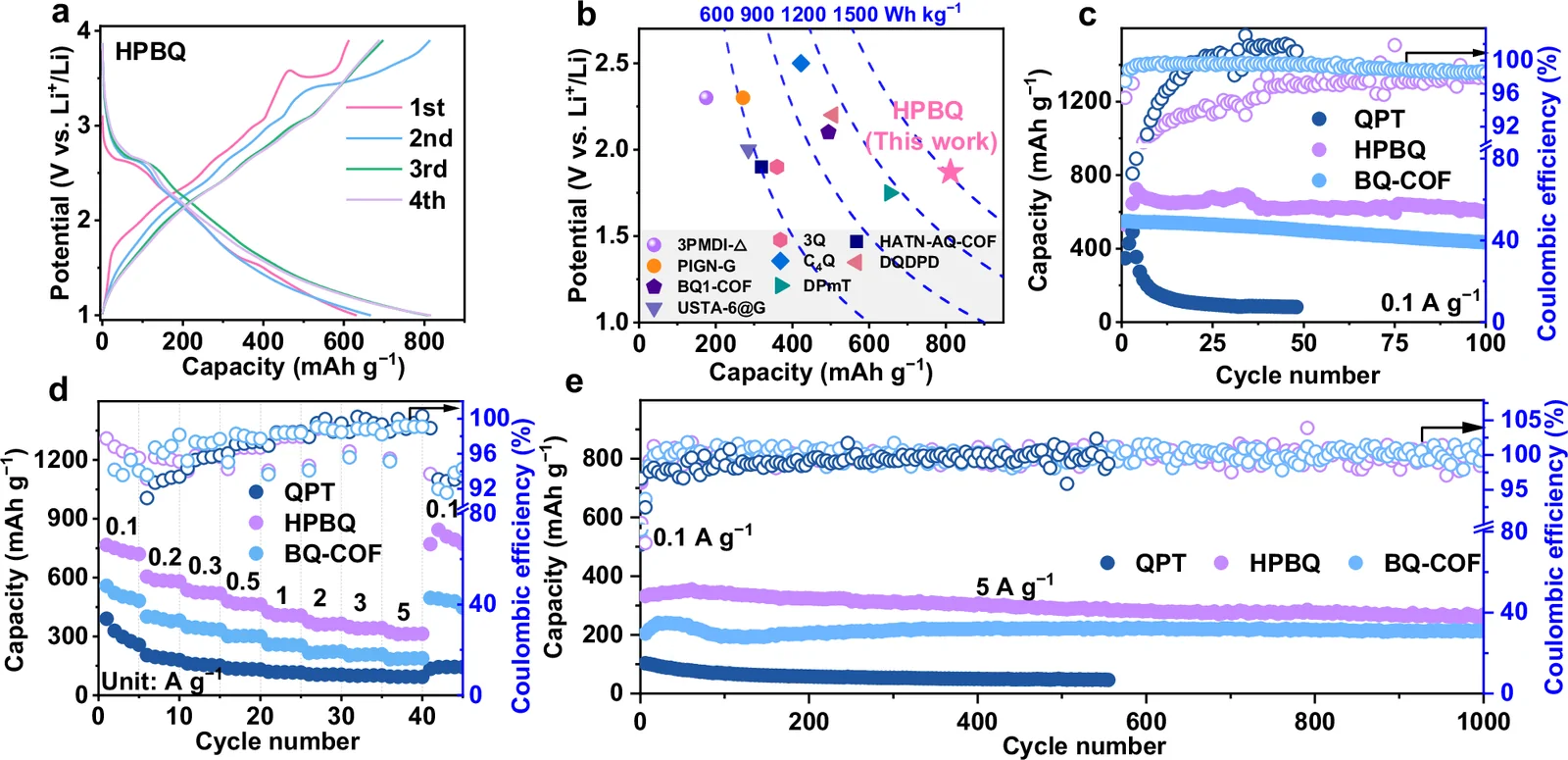
Electrochemical performance. **a** GCD profiles of HPBQ at 0.05 A g^−1^. **b** Comparison of capacity and specific energy of HPBQ with those of recently reported organic positive electrodes for LIBs. The source of the literature data shown in this figure can be found in Supplementary Information, Table S5. **c** Cycling performance of QPT, HPBQ, and BQ-COF at 0.1 A g^−1^.**d** Rate capability and **e** cycling stability at 5 A g^−1^ of HPBQ, QPT, and BQ-COF. Prior to the rate performance test, HPBQ and QPT were activated for 3 cycles at 0.1 A g^−1^.

The galvanostatic charge/discharge (GCD) profile of HPBQ at 0.05 A g^−1^ is presented in Fig. 3a, which revealed an initial discharge capacity of 630 mAh g^−1^, consistent with ~36-electron transfer per macrocyclic unit from lithiation of C = O and C = N groups. The first charge cycle yielded 615 mAh g^−1^ and exhibited a small plateau at 3.8 V (potential vs. Li^+^/Li), corresponding to irreversible conversion of ortho-hydroxyl groups to carbonyl moieties^46,55,56^. This plateau elongated during the second cycle, producing a high charge capacity of 804 mAh g^−1^. Subsequent cycles showed disappearance of this plateau, confirming complete hydroxyl-to-carbonyl conversion. This conversion process is more observed in the corresponding differential capacitance (dQ/dV) curves (Supplementary Fig. S25), which feature an additional oxidation peak at 3.6 V (potential vs. Li^+^/Li) during the initial two cycles that disappears by the third cycle. Moreover, the dQ/dV curves exhibited multiple pairs of redox peaks, indicating a multistep lithiation process. Similar phenomenon is observed in the cyclic voltammetry (CV) results (Supplementary Fig. S26). Remarkably, the discharge capacity subsequently stabilized at 806 mAh g^−1^ (ca. 48-electron transfer), with an average operating potential of ~1.87 V (potential vs. Li^+^/Li). This high reversible capacity is attributed predominantly to the HPBQ active material, as the conductive additive contributed negligible capacity within this potential range (Supplementary Fig. S27). Accordingly, HPBQ achieved a gravimetric specific energy of 1507 Wh kg^−1^, which is competitive to the reported organic positive electrode materials in LIBs (Fig. 3b and Supplementary Table S5). Notably, only C_6_O_6_ has been reported to achieve a slightly higher specific energy (1533 Wh kg^−1^)^27^, but it required ionic liquid electrolytes as well as an elevated operating temperature (70 °C).

The GCD curves of QPT are presented in Supplementary Fig. S28a, exhibiting an initial discharge capacity of 456 mAh g^−1^ (82.3% active-site utilization). During the first charge, a distinct plateau at approximately 3.4 V (potential vs. Li^+^/Li) was observed, corresponding to the hydroxyl-to-carbonyl transformation, which is also evident in the corresponding dQ/dV curves (Supplementary Fig. S29). This process led to an increased charge capacity of 523 mAh g^−1^ (92.5% active-site utilization). However, its capacity dropped to only 401 mAh g^−1^ by the second cycle, primarily due to material dissolution^50^. In contrast, BQ-COF exhibited a lower active-site utilization of 73% (564 mAh g^−1^, Supplementary Fig. S28b), attributed to inaccessible buried redox sites^57^. This result confirms the high active-site utilization achievable with SMOCCs. Notably, HPBQ achieved near-quantitative active-site utilization (99.2%), which is competitive with recently reported organic electrode materials (Supplementary Table S5).

Figure 3c and Supplementary Fig. S30 compare the cycling stability of the three compounds at 0.1 A g^−1^. The HPBQ electrode demonstrated an initial capacity of 612 mAh g^−1^ and reached a peak capacity of 756 mAh g^−1^ by the third cycle. It retained 80% of its peak capacity after 100 cycles, originating primarily from the dissolution resistance imparted by its rigid π-conjugated hexagonal monocyclic scaffold. The relatively low initial Coulombic efficiency might be attributed to partial irreversible Li^+^ trapping in the first cycle^32,58^. Nevertheless, during subsequent cycles, the trapped ions are gradually released while the redox-active sites undergo continuous activation, leading to a steady increase in Coulombic efficiency. The irregular cycling behavior of HPBQ may be attributed to its pronounced structural changes at low current densities. Similarly, BQ-COF retained 78% of its initial capacity after 100 cycles, benefiting from its rigid structure and low solubility. Its gradual capacity decay is primarily caused by kinetics hysteresis at low current densities, a phenomenon commonly observed in COFs-based electrodes^59^. In sharp contrast, QPT suffered rapid capacity degradation due to high solubility, retaining only 17% of its initial capacity after 50 cycles. These results demonstrate that our rationally designed HPBQ leverages its rigid π-conjugated scaffold and highly exposed redox centers to combine the advantages of SMOCCs (high active-site utilization) and COFs (long-term stability).

Rate performance of the three compounds was systematically assessed (Fig. 3d and Supplementary Fig. S31a–c). Prior to testing, QPT and HPBQ electrodes were pre-cycled for three cycles at 0.05 A g^−1^ to ensure complete hydroxyl-to-carbonyl conversion (Supplementary Fig. S31d, e). As expected, the capacity of QPT decreased rapidly as the current density increased, mainly due to dissolution in the electrolyte. In contrast, HPBQ delivered 788 mAh g^−1^ at 0.1 A g^−1^ and 305 mAh g^−1^ at 5 A g^−1^. Moreover, 97% of its initial capacity was recovered upon reverting to 0.1 A g^−1^, demonstrating good charge transport and structural integrity. Similarly, BQ-COF also demonstrated comparable rate performance, delivering 553 mAh g^−1^ at 0.1 A g^−1^ and 185 mAh g^−1^ at 5 A g^−1^. Furthermore, benefited from their rigid π-conjugated framework, both HPBQ and BQ-COF exhibited good cycling stability at high current densities. Specifically, HPBQ and BQ-COF achieved 81% and 92% capacity retention after 1000 cycles at 5 A g^−1^, respectively, outperforming QPT (41% capacity retention after 500 cycles). Furthermore, HPBQ demonstrated favorable electrochemical performance under high mass loading (Supplementary Fig. S32), in pouch-type lithium-ion batteries (Supplementary Fig. S33), and in coin-type sodium-ion batteries (Supplementary Fig. S34), further showcasing its promising practical prospects and versatility.

**Fig. 4:**
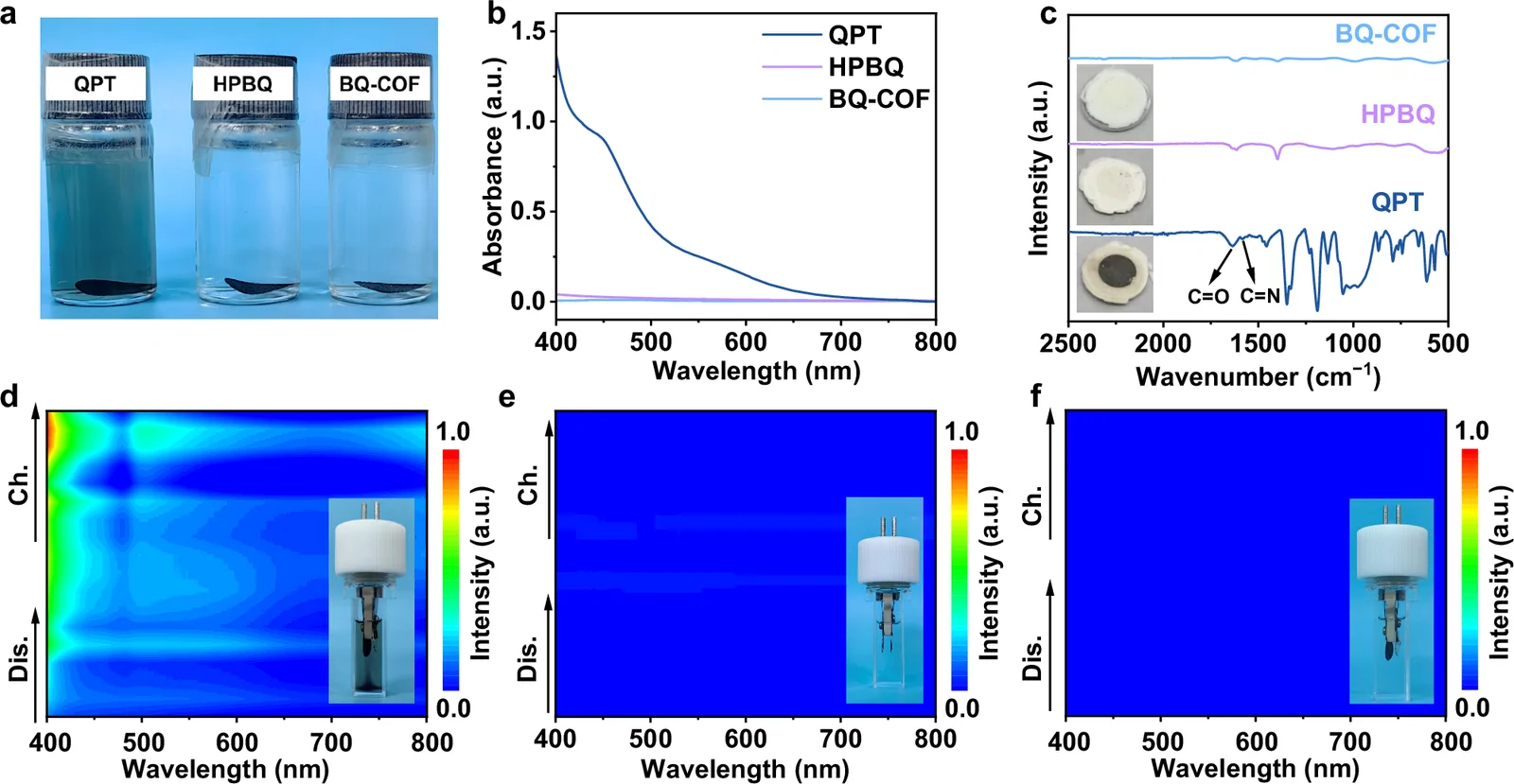
Electrodes solubility investigation. **a** Photographs of the QPT, HPBQ, and BQ-COF electrodes immersed in the 2 M LiTFSI (DOL/DME) electrolyte for 48 h. **b** UV–Vis spectra of the electrolytes after immersion. **c** ATR-FTIR spectra of the separators harvested from the cell at the charged state after 20 cycles at 0.3 A g^−1^ under 25 ± 1 °C. Insets show the photographs of these separators. In situ UV–Vis spectroscopy monitoring the discharging/charge processes of **d** QPT, **e** HPBQ, and **f** BQ-COF electrodes at 25 ± 1 °C and 0.3 A g^−1^ (Insets show photographs of the respective electrolytes in the fully charged state).

To elucidate the cycling stability disparities among QPT, HPBQ, and BQ-COF, we systematically investigated their solubility in electrolyte. Electrode stability was assessed through 48-h electrolyte immersion tests, which revealed significant colour variation for QPT, whereas solutions with HPBQ or BQ-COF remained optically transparent (Fig. 4a). Subsequent UV–Vis analysis of soaked electrolytes (Fig. 4b) exhibits a distinct absorption peak for QPT, suggesting dissolution. In contrast, the electrolytes soaked with HPBQ and BQ-COF show negligible absorption, indicating minimal dissolution of the materials in the electrolyte.

In addition, the separators of the cells after cycling were characterized. As shown in the inset of Fig. 4c, no visible deposits were observed on the separators retrieved from cells based on HPBQ and BQ-COF, while black deposits were evident on QPT-derived separator. ATR-FTIR analysis of QPT-derived separator confirmed material dissolution through distinct characteristic peaks (C = O stretching vibration at 1631 cm^−1^ and C = N stretching vibration at 1584 cm^−1^). Conversely, separators from the BQ-COF and HPBQ cells exhibited no characteristic peaks corresponding to the active materials. In situ UV–Vis spectroscopy monitoring directly observed the dissolution behavior of the three electrodes (Fig. 4d–f and Supplementary Fig. S35). During cycling, QPT exhibited gradually intensifying characteristic absorption peaks in the range of 400–700 nm (Fig. 4d and Supplementary Fig. S35a), and visibly dissolved species were observed in the post-cycling electrolyte (inset of Fig. 4d), indicating dissolution of the active material. In contrast, neither HPBQ (Fig. 4e and Supplementary Fig. S35b) nor BQ-COF (Fig. 4f and Supplementary Fig. S35c) showed detectable absorption features, with their electrolytes retaining high optical clarity. Furthermore, elemental mapping images of the pristine QPT, HPBQ, and BQ-COF electrodes confirm the homogeneous distribution of C, N, and O, indicating good electrode uniformity (Supplementary Fig. S36). No significant differences were observed in the SEM images of the three electrodes before and after cycling (Supplementary Fig. S37), even though QPT is partially dissolved in the electrolyte. These results validate that our rationally designed macrocyclic molecule HPBQ achieves dissolution resistance comparable to COF materials, thus ensuring long-term cycle stability.

### Charge storage mechanism

**Fig. 5:**
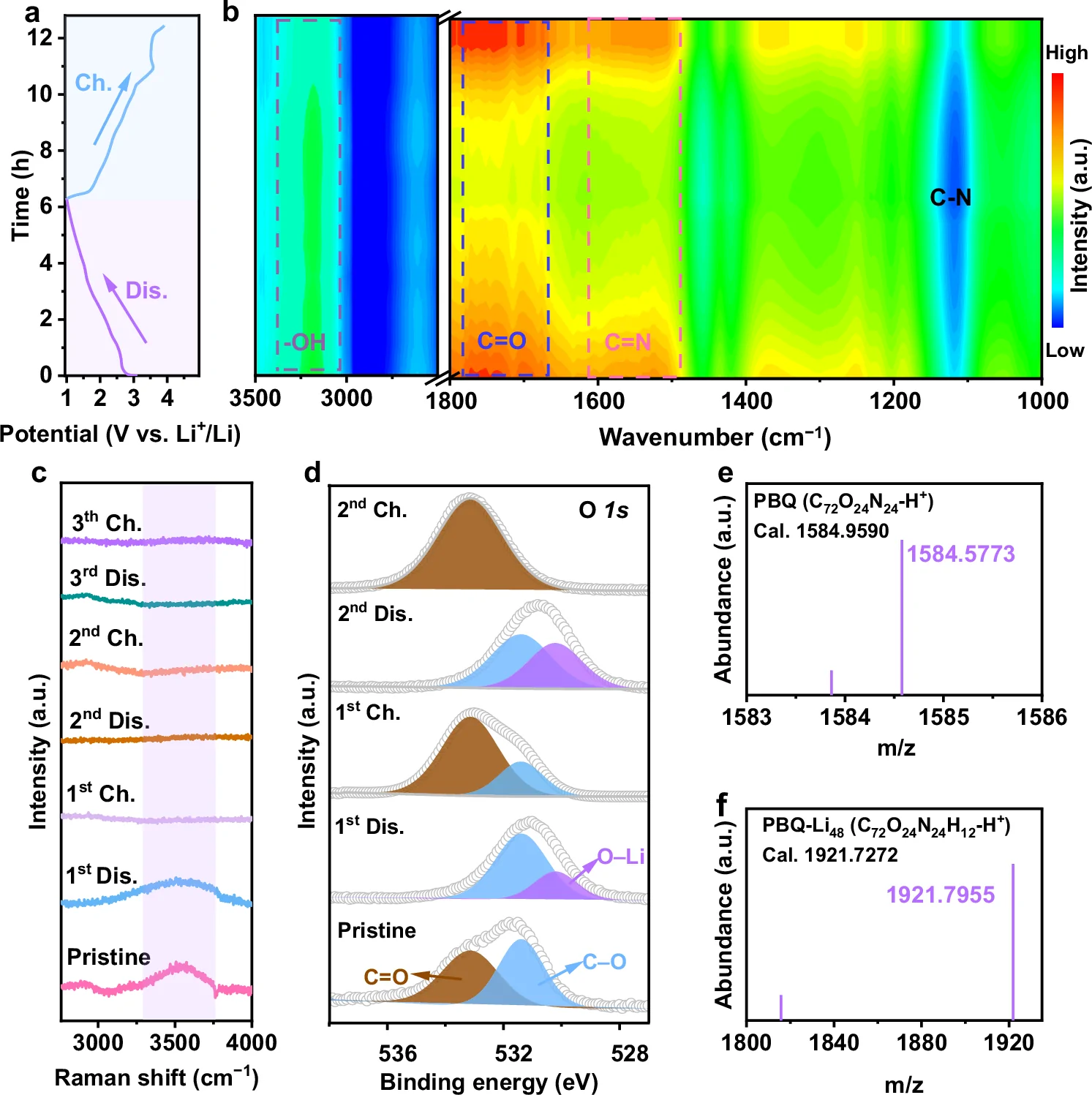
Spectroscopic characterizations revealing the charge storage mechanism of HPBQ. **a** Potential–capacity curve and **b** corresponding in situ FTIR spectra collected during the initial cycle at 0.2 A g^−1^ and 25 ± 1 °C. **c** Raman spectra and **d** O *1 s* XPS spectra of HPBQ at different discharging/charging states. All electrodes were taken at fully charged and fully discharged states from cells cycled at 0.1 A g^−1^ and 25 ± 1 °C. The cycle number is labeled in the figure. LC–HRMS analysis of HPBQ electrodes at **e** 3^rd^ charged and **f** 3^rd^ discharged states. Cells were cycled at 0.1 A g^−1^ and 25 ± 1 °C.

Various spectroscopic techniques were conducted to reveal the charge storage mechanism of HPBQ. Figure 5a, b presents the in situ FTIR results during the initial cycle. The characteristic peaks of C = O (1680 cm^−1^) and C = N (1533 cm^−1^) gradually weakened and almost disappeared upon discharge, then intensified during the charging process. This confirms these groups act as redox-active centers. Intriguingly, the intensity of the –OH peak at 3216 cm^−1^ remained largely unaltered during discharge but weakened in the later charging stage, suggesting partial dehydrogenation of hydroxyl groups occurred at this stage. Ex situ FTIR spectroscopy revealed consistent results (Supplementary Fig. S38). Similar findings were obtained from in situ Raman spectroscopy analysis (Supplementary Fig. S39). The vibrational intensities of the C = O and C = N bonds decreased during discharge and increased during charge, further confirming their role as redox centers. To track the transformation of –OH, the Raman spectra of the HPBQ electrode after multi-cycles were collected (Fig. 5c). The characteristic –OH peak at 3478 cm^−1^ exhibited progressive attenuation upon initial charging and completely vanished by the third cycle, indicating the complete dehydrogenation and irreversible structural transformation from HPBQ to PBQ.

Ex situ XPS analysis further corroborates this mechanism. The O *1 s* XPS spectrum of pristine HPBQ (Fig. 5d) shows two peaks at 533.1 eV (C = O) and 531.4 eV (C–O)^13^. Notably, the C–O peak largely diminished at the 1^st^ charge state and was absent by the 2^nd^ charge state, confirming irreversible conversion of hydroxyl groups to carbonyl moieties (i.e., from HPBQ to PBQ). Concurrently, a new peak assigned to C–O–Li emerged at the fully discharged state and vanished upon recharging, indicating reversible lithiation/delithiation at carbonyl sites^60^. Similarly, N *1s* spectra (Supplementary Fig. S40a) reveal disappearance of C = N (398.8 eV) during discharging state and its reappearances upon charging, accompanied by reciprocal changes in the C–N–Li peak (397.5 eV), confirming reversible lithiation/delithiation of C = N. This behavior is consistent with Li *1 s* spectral evolution (Supplementary Fig. S40b), which corroborates the FTIR and Raman results regarding redox-active groups and irreversible HPBQ-to-PBQ transformation.

To further validate this irreversible transformation, LC–HRMS analysis was performed on the fully charged/discharged electrodes at the 3^rd^ cycle. The fully charged sample exhibited a distinct peak at *m/z *= 1584.5773, consistent with the theoretical m/z for protonated PBQ [(C_72_N_24_O_24_-H^+^)^−^, *m/z* = 1584.9590] in positive mode (Fig. 5e). Moreover, the discharged sample showed a distinct peak at m/z = 1921.7955, attributed to lithiation specie PBQ–48Li [(C_72_N_24_O_24_Li_48_-H^+^)^−^, *m/z* = 1921.7272] in negative mode (Fig. 5f). Furthermore, inductively coupled plasma optical emission spectrometry (ICP-OES) was conducted to determine the number of transferred lithium ions at the 1^st^ discharged state (corresponding to a 36-electron reaction per macrocyclic unit) and the 3^rd^ discharged state (corresponding to a 48-electron reaction per macrocyclic unit) (Supplementary Table S6). The experimentally measured numbers of intercalated lithium ions are 33.14 and 42.73, respectively, which agree well with the theoretical values of 36 and 48. These results unambiguously demonstrate the irreversible structural evolution from HPBQ to PBQ, which ensures the proposed reversible 48-electron-per-macrocyclic-unit lithium storage mechanism.

**Fig. 6:**
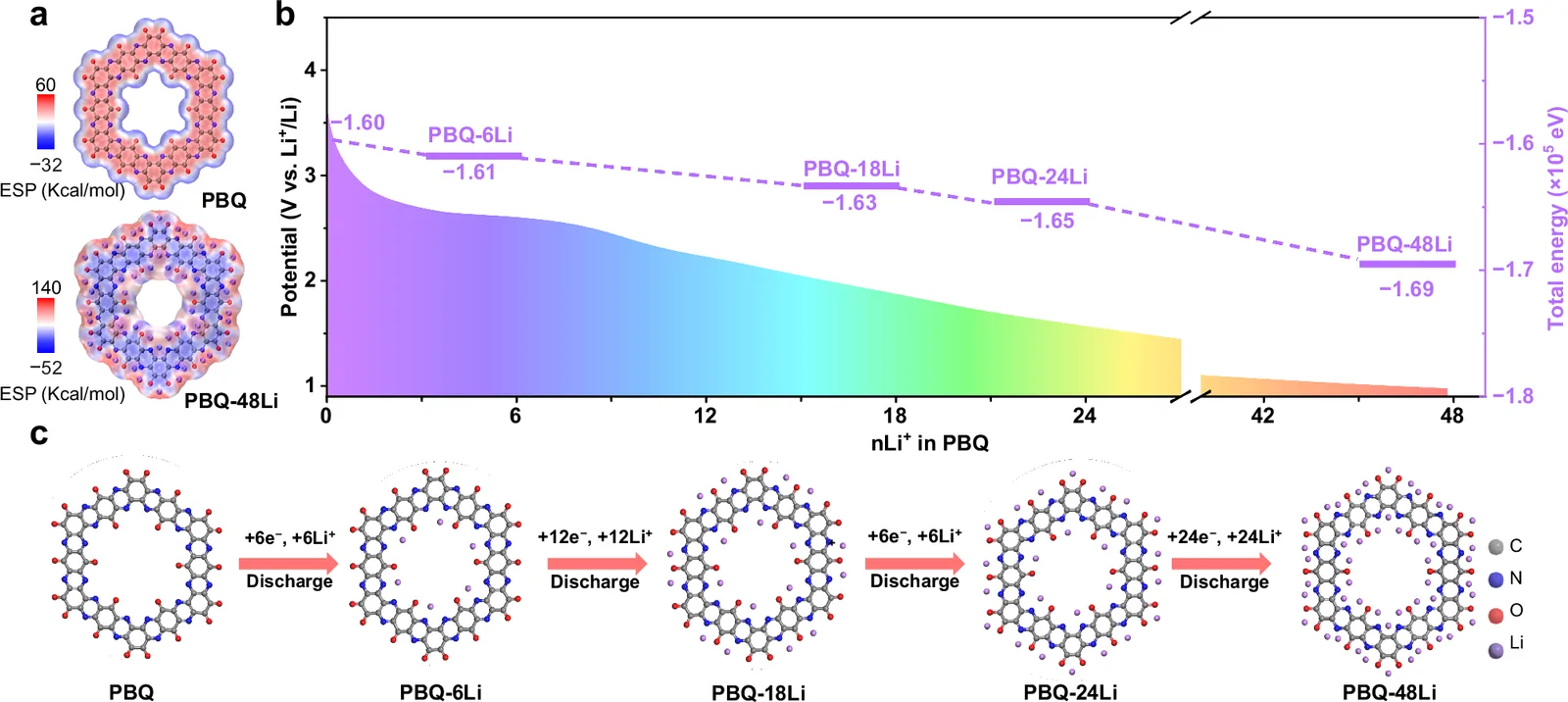
DFT calculations on PBQ. **a** Mapped ESP diagram of the PBQ/PBQ–48Li on the van der Waals surface. **b** Proposed lithiation pathway of PBQ obtained through simulation. The left axis displays the redox potential versus Li/Li^+^. The right axis presents the total energy (eV)—defined as the sum of electronic and zero-point energies—for various lithiation PBQ structures; the most stable configuration at each lithiation stage is highlighted by a green line. **c** Structural evolution of PBQ during the lithiation process.

To clarify the redox mechanism of PBQ, systematic theoretical calculations were performed. DFT calculations for PBQ (Fig. 6a and Supplementary Data 2) show maximum negative charge density (blue regions) around C = O and C = N groups, corresponding to 48 accessible Li^+^ intercalation sites per macrocyclic unit. This yields a theoretical capacity of 812 mAh g^−1^, consistent with the experimental capacity of HPBQ (806 mAh g^−1^). Furthermore, analysis of the electrostatic potential (ESP) reveals three distinct clusters of negative values, indicating varying activity of among the active sites (Supplementary Fig. S41), in the order of Site A (− 31.6 kcal/mol) >Site B (− 30.6 kcal/mol) > Site C (− 26.6 kcal/mol). This trend provides definitive evidence for a multistep lithiation sequence in PBQ^61^. Specifically, the initial stage involves the insertion of 6 Li^+^ ions into the most active Site A, with each ion establishing an O–Li–N coordination. Subsequently, 12 Li^+^ ions insert into Site B, also forming an O–Li–N configuration. Subsequently, 6 Li^+^ ions are accommodated at Site C, where they are stabilized by an O–Li–O coordination. The high spatial density of these active sites induces steric hindrance, thereby restricting each extremum point to a single Li^+^ ions. Ultimately, the remaining active sites undergo deep lithiation, accommodating a further 24 Li^+^ ions. The calculated total energy changes (Fig. 6b) and theoretical reduction potential variations (Supplementary Fig. S42) throughout this process corresponds to four intermediate stages: HPBQ-6Li, HPBQ-18Li, HPBQ-24Li, and HPBQ-48Li, with concomitant structural evolution illustrated in Fig. 6c and Supplementary Data 3.

## Discussion

This work presents a macrocyclic molecular engineering strategy that successfully reconciles the long-standing trade-off between structural stability and redox efficiency in organic positive electrode materials. By synergistically integrating dense redox-active moieties (C = O, C = N, –OH) within a rigid π-conjugated hexagonal monocyclic framework, the rational designed HPBQ achieved a synergy of high reversible capacity (806 mAh g^−1^, corresponding to 99.2% active site utilization), high specific energy (1507 Wh kg^−1^ based on HPBQ mass), and good cycle stability (81% retention after 1000 cycles at 5 A g^−1^). The deprotonation-induced irreversible HPBQ-to-PBQ transformation during initial cycling enables a sustained 48-electron redox chemistry per macrocyclic unit as well as high-capacity delivery, while the rigid π-conjugated skeleton effectively suppresses dissolution, ensuring long-term cycling stability. Our strategy of embedding COF-inspired structural robustness into a small-molecule design overcomes the intrinsic limitations of conventional organic electrodes, establishing a blueprint for resource-efficient, high-energy-density batteries critical for sustainable electrification.

## Methods

### Materials

All reagents, including tetrachloro-p-benzoquinone (TCB, Macklin, 98.0%), potassium phthalimide (PPL, Macklin, 98.0%), acetonitrile (ACN, Macklin, 99.8%), N, N-dimethylformamide (DMF, Aladdin, 99.5%), hydrazine hydrate (Macklin, 80.0 wt%), disodium rhodizonate (Na_2_C_6_O_6_, Aladdin, 95.0%), acetic acid (AC, Macklin, 99.9%), methanol (Macklin, 99.9%), acetone (Sinopharm, AR), toluene (Sinopharm, AR), 2,3-dichloronaphthalene-1,4-dione (DPD, Macklin, 98.0%), potassium phthalimide (PPL, Macklin, 98.0%), cyclohexanehexone (C_6_O_6_, Aladdin), dimethyl sulfoxide-d6 (DMSO-d6, Aladdin, 99.9%), lithium hexafluorophosphate (LiPF_6_, DoDoChem, 99.9%), ethylene carbonate (EC, Adamas, 99.9%), diethyl carbonate (DEC, Adamas, 99.0%), lithium bis((trifluoromethyl)sulfonyl)azanide (LiTFSI, DoDoChem, 99.9%), lithium bis((trifluoromethyl)sulfonyl)azanide 1,3-dioxolane (DOL, Adamas, 99.9%), 1,2-dimethoxyethane (DME, Adamas, 99.0%), poly(vinylidene fluoride) (PVDF, Solef 5130, Mw ≈ 7.0 × 10^5 ^g/mol) binder, graphene oxide/carbon nanotube (G/C, 1:1 mass ratio), acetylene black (AB, Canrd), Ketjen black (KB, Sinero), Super P (SP, TIMCAL), were commercially available and directly used without further purification. Coin cell components (spacer:15.8 ×  1.0 mm (diameter × thickness), spring:15.4 × 1.2 × 0.3 mm (outer diameter × inner diameter × thickness)), lithium foil (99.9%, 15.6 × 0.45 mm (diameter × thickness)), sodium foil (14 ×  0.45 mm (diameter × thickness)), were purchased from Canrd. Glass microfiber membrane (thickness: 675 μm, lateral dimension: 15 mm, pore size: 2.7 μm, one separator for one battery) was supplied by Whatman. Al@C foil (thickness: 15 μm) was obtained from Shenzhen Jingliang Copper Industry Co., Ltd.

### Synthesis of tetra(phthalimido)-benzoquinone (TPB)

The preparation of TPB was conducted following a modified procedure based on a previous report^62^. Under a nitrogen atmosphere, 12.3 g of TCB and 37.2 g of PPL were added to 250 mL of ACN. The mixture was heated to 80 °C and stirred for 24 h. After cooling to room temperature, the reaction products were filtered and washed multiple times with DMF and deionized water at 100 °C. The obtained solid was then suspended in 150 mL of boiling ethanol and vacuum filtered. Finally, the product was dried in a vacuum oven at 100 °C for 12 h, yielding 30 g of TPB (yield 85.0%) as a brown-yellow powder.

### Synthesis of 2,3,5,6-tetraaminobenzoquinone (TABQ)

The synthesized TPB was transferred into a 500 mL round bottom flask, into which 200 mL of hydrazine hydrate was added. After being kept at 65 °C for 2 h, purple TABQ was obtained (yield 66%). ^1^H NMR (400 MHz, DMSO-d6): δ = 4.54 ppm (s, 1H); ^13^C NMR (101 MHz, DMSO-d6): δ = 178.52, 121.45 ppm; LC–HRMS (m/z): [M]^+^ calcd. for C_6_H_8_N_4_O_2_, 169.0612; found, 169.0875.

### Synthesis of integrates a π-conjugated core with densely packed electroactive moieties (C = O, –OH, C = N) (HPBQ)

In a three-mouth flask, TABQ (84.08 mg, 0.5 mmol) and Na_2_C_6_O_6_ (107.02 mg, 0.5 mmol) were added into acetic acid (70 mL) and deionized water (30 mL), followed by evacuating and refilling the flask with Ar for three times and refluxing for 48 h. After cooling to room temperature, the product was successively washed with deionized water, methanol, and acetone until the washings showed no color change. It was then refluxed in methanol for 24 h and filtered hot. Subsequently, the solid was refluxed in toluene for 24 h and filtered hot again. Following freeze-drying, the material was dried under vacuum at 180 °C for 24 h to afford a black solid (yield 76%). m.p. = ~167 °C (Supplementary Fig. S43). ^13^C NMR (101 MHz, Magic Angle Spinning): δ = 174.46, 169.02, 146.03, 144.32, 131.41, 129.96 ppm; LC–HRMS (m/z): [M]^+^ calcd. for C_72_H_12_N_24_O_24_, 1597.0529; found, 1597.0787.

### Synthesis of 2-(1,4-dioxo-3-(1-oxoisoindolin-2-yl)−1,4-dihydronaphthalen-2-yl) isoindoline-1,3-dione (DOD)

The preparation of DND was conducted following a modified procedure based on a previous report^32^. Under a nitrogen atmosphere, 22.5 g of DPD and 46.2 g of PPL were added to 400 mL of acetonitrile. The mixture was heated to 80 °C and stirred for 24 h. After cooling to room temperature, the reaction products were filtered and washed multiple times with ethanol and deionized water at 100 °C. Finally, the product was dried in a vacuum oven at 100 °C for 12 h, yielding 42 g of DOD (yield 91.3%) as a yellow powder.

### Synthesis of 2,3-diaminonaphthalene-1,4-dione (DND)

The synthesized DOD was transferred into a 500 mL round bottom flask, into which 300 mL of hydrazine hydrate was added. After being kept at 65 °C for 2 h, purple DND was obtained (yield 72%). ^1^H NMR (400 MHz, DMSO-d6): δ 7.73 ppm (m, 1H), 7.57 ppm (m, 1H), 5.42 ppm (s, 2H). LC–HRMS (*m/z*): [M]^+^ calcd. for C_10_H_8_N_2_O_2_, 189.0678; found, 189.0901.

### Synthesis of the 7,8-dihydroxybenzo[i]benzo[6,7]quinoxalino[2,3-a]phenazine-5,10,15,18-tetraone (QPT)

In a three-mouth flask, DND (376 mg, 2 mmol) and Na_2_C_6_O_6_ (214 mg, 1 mmol) were added into acetic acid (140 mL) and water (60 mL), followed by evacuating and refilling the flask with Ar for three times and refluxing for 24 h. After cooling to room temperature, the solid was washed with water, methanol, acetone and then dried under vacuum to give a dark solid (yield 86%). ^1^H NMR (400 MHz, DMSO-d6): δ 9.61 ppm (s, 1H), 8.34 ppm (m, 2H), 8.03 ppm (m, 2H). LC–HRMS (m/z): [M]^+^ calcd. for C_26_H_10_N_4_O_6_, 475.0698; found, 475.3275.

### Synthesis of the BQ-COF

C_6_O_6_ (250 mg) and TABQ (200 mg) were placed in a three-necked round-bottom flask under an argon atmosphere. A mixture of acetic acid and deionized water (150 mL, *v*/*v* = 1:1) was slowly added to the flask, followed by vigorous stirring for 30 min at room temperature. The reaction system was then heated to 120 °C and maintained at this temperature for 3 days under continuous stirring. The resulting solid product was collected by centrifugation and sequentially washed with deionized water, methanol, and acetone to remove unreacted precursors and residual solvents. The obtained sample was further purified via Soxhlet extraction using deionized water and methanol. Finally, the purified material was vacuum-dried at 80 °C for 12 h to yield the target BQ-COF. ^13^C NMR (101 MHz, Magic angle spinning): δ = 178.26, 142.51, 135.33 ppm.

### Characterizations

FTIR spectra were acquired with a BRUKER TENSOR II (FTS6000) spectrometer via the KBr pellet technique, covering the 400 and 4000 cm^–1^ region. Raman spectra were recorded using a Raman microscope (Renishaw plc (invia-reflex)) with a 488 nm laser. The morphologies of samples were investigated using field emission scanning electron microscopy (SEM, TESCAN MIRA3) along with energy-dispersive X-ray spectroscopy (EDX) (the voltage is set to 15 kV). Transmission electron microscopy (TEM) imaging was performed on a Talos F200i microscope operated at an accelerating voltage of 200 kV. X-ray diffraction (XRD) patterns were conducted by the Bruker D8 Advance with Cu Kα (λ = 0.154 nm) radiation. Data were collected over a 2*θ* range of 5–50° at a scan rate of 2° min^–1^ with a step size of 0.02°. UV-visible absorption spectra were recorded from Shimadzu UV-1900i. Thermo gravimetric analysis (TGA) was carried out on a TGA/DSC3+ thermal analysis system (STA 700, Japan) from room temperature to 800 °C at a heating rate of 10 °C min^−1^ in N_2_ atmosphere. Melting points were determined by differential scanning calorimetry (DSC) using a NETZSCH DSC204F1 instrument. Liquid chromatography high-resolution mass spectrometry (LC–HRMS) analysis results were acquired from Thermo Fisher Orbitrap Exploris 120. HPBQ was dissolved in methanol at an ultra-trace concentration (below 0.5 ppm) for the analysis. X-ray photoelectron spectroscopy (XPS) measurements were conducted on an X-ray photoelectron spectrometer (Thermo Fischer, ESCALAB Xi + ) under a vacuum of 8 ×  10^–10^ Pa. All of the binding energies were referenced to the C 1 s peak at 284.6 eV. The ^1^H and ^13^C nuclear magnetic resonance (NMR) were conducted on a 400 MHz NMR spectrometer (Bruker Advance III HD). The solid-state ^13^C NMR with Inova 400 MHz Spectrometer (Bruker Avance Neo). Inductively coupled plasma optical emission spectroscopy (ICP-OES) was conducted on an Agilent 720ES spectrometer (USA). Elemental composition (C, H, O, N) was measured on an Elementar Vario EL III elemental analyser. For ex situ FTIR, XPS, and SEM measurements, the cells were cycled to a specific state of charge at 0.1 A g^–1^, and then disassembled in a Ar-filled glovebox with [O_2_], [H_2_O] ≤1 ppm to retrieve the used electrodes. Before each test, the cycled electrodes were cleaned with the electrolyte solvent in the same glovebox. In situ spectroscopic measurements, including FTIR, UV-Vis, and Raman spectroscopy, employed a consistent two-electrode configuration with the organic material (HPBQ or otherwise specified) as the positive electrode and lithium metal as the negative electrode. Cells were assembled in an argon-filled glovebox using 2 M LiTFSI in DOL/DME (1:1 v/v) as the electrolyte. Specifically, in situ FTIR utilized a cell with a monocrystal silicon prism as the bottom window, on which the working electrode was mounted. In situ UV-Vis and Raman experiments were carried out in customized cuvette cells, sealed with 3 ml and 0.1 ml of electrolyte, respectively.

### Electrochemical measurements

The electrochemical properties of the organic electrodes were evaluated using a 2032-type coin cell, with a lithium foil or sodium foil as the counter electrode, a glass microfiber membrane as the separator. Three electrolyte formulations were evaluated, including 2 M LiPF_6_ EC/DEC (1:1 by vol.), 2 M LiTFSI EC/DEC (1:1 by vol.), and a 2 M LiTFSI-DOL/DME (1:1 by vol.), with the 2 M LiTFSI-DOL/DME (1:1, by vol.) formulation as the optimal electrolyte. Working electrodes were prepared by mixing 60 wt% organic materials (HPBQ, QPT, or BQ-COF), 30 wt% conductive carbon (G/C, AB, KB, or SP), and 10 wt% poly(vinylidene fluoride) binder in N-methyl-2-pyrrolidinone (NMP) to form a slurry, which was then cast onto Al@C foil by doctor blading. The electrode was dried overnight at 80 °C in a vacuum oven, and punched into 10 mm-diameter electrode discs using a manual punch (Shenzhen Kejing Star Technology Co., Ltd., MSK-T10). For most electrochemical tests, the areal mass loading of active materials was controlled within 0.8–1.2 mg cm^−2^. In addition, HPBQ electrodes with mass loadings varying from 1 to 10 mg cm^−2^ were also prepared to investigate loading-dependent electrochemical behavior. A fixed dosage of 100 μL of electrolyte was used in each coin cell. All cells were assembled in an Ar-filled glove box with [O_2_], [H_2_O] ≤1 ppm.

Galvanostatic charge–discharge (GCD) tests on HPBQ electrodes were first conducted on a Neware battery tester to screen suitable electrochemical windows, including 1.0–3.9 V, 1.0–3.5 V, 1.0–4.2 V, and 0.5–3.9 V. To simultaneously achieve high capacity and stable cycling, the 1.0–3.9 V window was ultimately selected for electrochemical evaluation in LIBs. The differential capacitance (dQ/dV) profiles of the HPBQ and QPT electrodes were derived from their galvanostatic charge/discharge profiles (at 0.05 A g^−1^), representing the rate of charge variation with voltage. The Coulombic efficiency (CE) is calculated as the ratio of discharge capacity divided by the charge capacity in the preceding charge cycle. Cyclic voltammetry (CV) measurements were performed with an Admiral Instruments electrochemical workstation within a potential of 1.0–3.9 V and a scan rate of 0.2 mV s^−1^.

Single‑layer pouch cells were assembled using an HPBQ positive electrode (Al@C current collector, areal loading 1 mg cm^−2^, dimensions 47 × 77 mm), a lithium‑metal negative electrode (purity > 99.99 %, thickness 100 µm, dimensions 47 × 77 mm) with a negative‑to‑positive capacity ratio (N/P) of 17:1, a glass‑fibre separator (Whatman, 47 × 77 mm), and 1.5 g of 2 M LiTFSI-DOL/DME (1:1, by vol.) electrolyte. For each electrochemical measurement, two identical Li | |HPBQ pouch cells were assembled, and the data presented in the manuscript figures were obtained from one representative cell. All electrochemical tests were performed at 25 ± 1 °C. The coin‑cell data presented in the manuscript figures were obtained from a single representative cell selected from three independent replicate tests.

The theoretical specific capacity (*Q*) was calculated using Eq. (1):

1 $$Q=\frac{{nF}}{3.6{M}_{W}}$$

where *n* is the number of transferred electron, *F* is Faraday’s constant (96,485.33 C/mol), and ${M}_{W}$ is molecular mass (1596 g mol^−1^ for HPBQ). Based on a 48-electron transfer per macrocyclic unit for HPBQ, this yields a theoretical capacity of 812 mAh g^−1^.

The gravimetric specific energy (*E*_D_, Wh kg^−1^) of HPBQ was determined using the following equation:

2 $${E}_{D}=\frac{{CV}}{m}$$

where *C* is the measured capacity (Ah), *V* is average discharge potential (V), and *m* is mass of HPBQ (kg). Based on the maximum capacity of HPBQ (806 mAh g^−1^) and average operating potential (1.87 V) at 0.05 A g^−1^, this yields a gravitational specific energy of 1507 Wh kg^−1^.

### Computational method

Geometry optimizations, frontier orbitals, and molecular electrostatic potentials were performed using B3LYP-D3(BJ) hybrid density functional with the 6–31 G(d) basis sets implemented by Gaussian 16 program package^63^. The frontier orbitals and molecular electrostatic potentials were visualized by Multiwfn and visual molecular dynamics^64–66^.

To calculate the reduction potential of the PBQ, all molecules were incorporated to account for the solvent effect using PCM model with the dielectric constant (ε) of 7.155 for DOL/DME (ε_DOL_ = 7.13 and ε_DME_ = 7.18)^67^. Note that, to reduce the calculation cost, an average ε value was employed for the 1:1 DOL/DME mixture solvent without solvation cavity modification. Frequency calculations were executed at the B3LYP-D3 hybrid density functional with the def2tzvp basis sets at a temperature of 298 K and a pressure of 1 atm.

The reduction potential of each step was calculated by the following equations:

3 $${{{{\rm{Li}}}}}_{{{x}}-{{{\rm{n}}}}}{{{\rm{PBQ}}}}+n{{{\rm{Li}}}}\to {{{{\rm{Li}}}}}_{{{x}}}{{{\rm{PBQ}}}}$$

4 $${E}_{x}=-\Delta {G}_{x}/{nF}=-[G({{{{\rm{Li}}}}}_{x}{{{\rm{PBQ}}}})-G({{{{\rm{Li}}}}}_{{x}-{{{\rm{n}}}}}{{{\rm{PBQ}}}})-{{{{\rm{n}}}}G}({{{\rm{Li}}}})]/F$$

where *E*_*x*_ is theoretical reduction potential (V *vs*. Li^+^/Li) of the *x*th step, *G* is the Gibbs free energy of the specified molecule in the bracket [Ha, *G*(Li) = −7.5215 Ha], Δ*G* is the Gibbs free energy gap between products and reactants (J mol^−1^, 1 Ha = 2,625,500 J mol^−1^), *n* is the transferred electron number of the reaction, and *F* is the Faraday constant (96,485 C mol^−1^).

## Supplementary information

Supporting Information

Description of Additional Supplementary Files

Supplementary Data 1-3

Transparent Peer Review file

## Source data

Source Data

## Data Availability

All data that support the findings of this study are presented in the manuscript and Supplementary Information. Any other data that support the findings of this study are also available from the corresponding authors on request. Source data are provided with this paper.
