# Supplementary material for: Macrocyclic molecular engineering enables high-energy and durable organic electrode for lithium-ion batteries
Source: Nat Commun. 2026 Aug 29;17:10312. doi: 10.1038/s41467-026-76984-5 (PMC13620139; doi:10.1038/s41467-026-76984-5)
Supplement: Supplementary file 1 [file 41467_2026_76984_MOESM1_ESM.pdf]

Supplementary Information

Macrocyclic Molecular Engineering Enables High-Energy and Durable Organic Electrode for Lithium-Ion Batteries

Huiling Peng[1,†], Yaheng Geng[1,†], Yu Han[1,†], Jin Xiao[2], Zehao Yu[1], Zichao Yan[1], and Zhiqiang Zhu[1,*]

[1] State Key Laboratory of Chemo and Biosensing, College of Chemistry and Chemical Engineering, Hunan University, Changsha 410082, China

[2] School of Science, Hunan University of Technology, Zhuzhou 412007, China

[†]These authors contributed equally to this work

Email: zqzhu@hnu.edu.cn

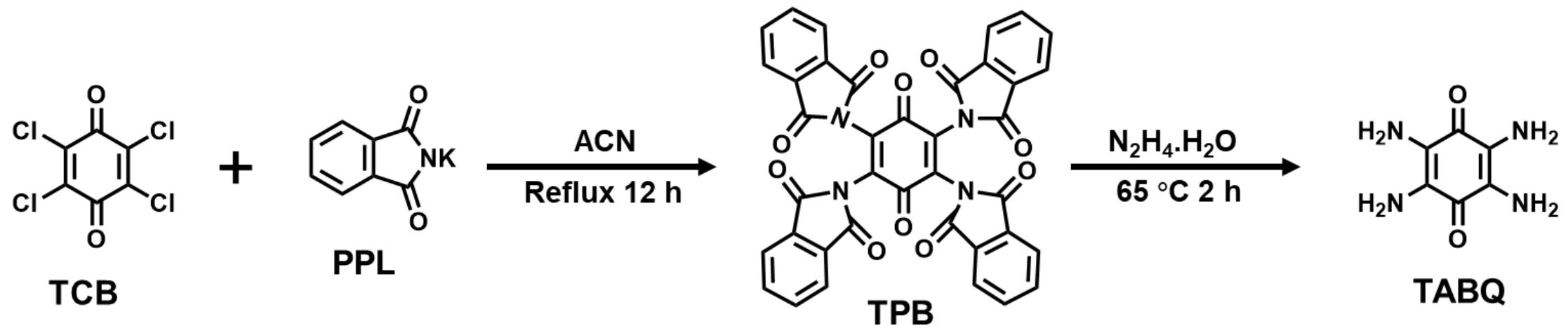

Supplementary Fig. S1 Synthetic routes of TABQ.

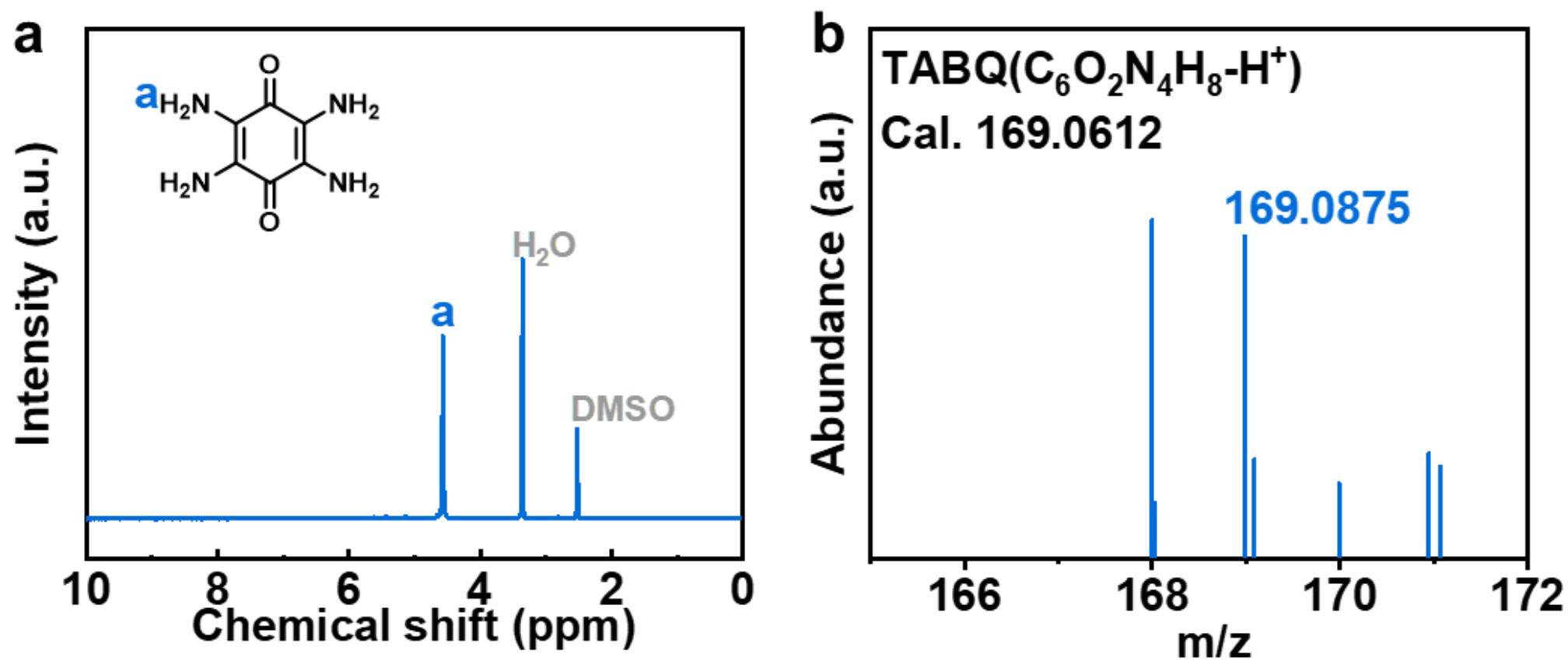

Supplementary Fig. S2 Characterizations of TABQ. **a** ^{1}H NMR and **b** LC-HRMS of TABQ.

The ^{1}H NMR spectrum of TAPQ exhibits a single signal at 4.5 ppm (Supplementary Fig. S2a), corresponding to the amino protons ($–NH_2$), indicating that these protons reside in a uniform chemical environment. Furthermore, LC-HRMS analysis (Supplementary Fig. S2b) reveals a prominent peak at m/z=169.0612 in positive ion mode, which is consistent with the calculated m/z value for protonated TABQ ($[C_6H_8N_4O_2+H]^+$, theoretical m/z = 169.0875), thereby confirming the successful synthesis of TABQ.[1]

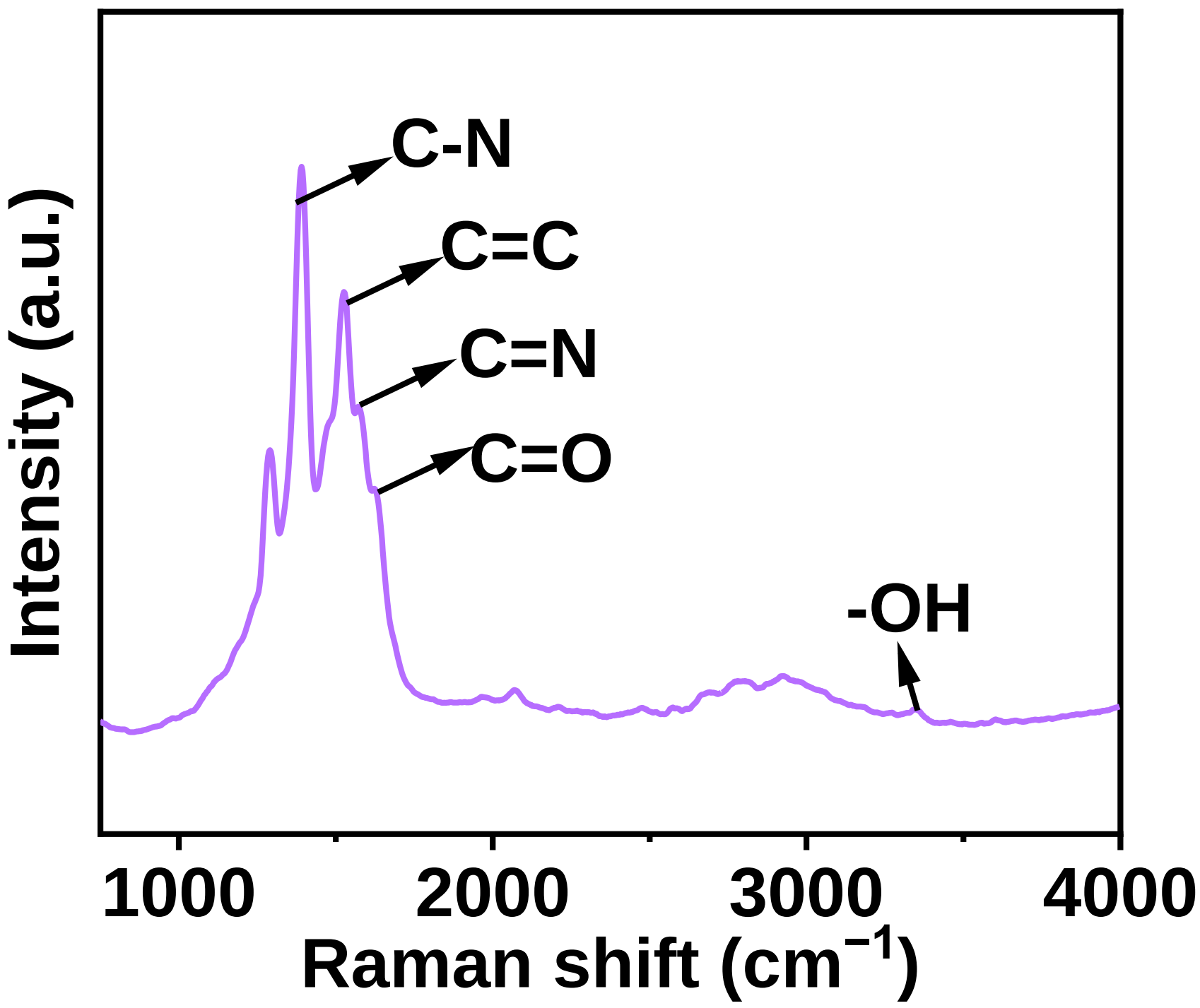

Supplementary Fig. S3 Raman spectrum of HPBQ.

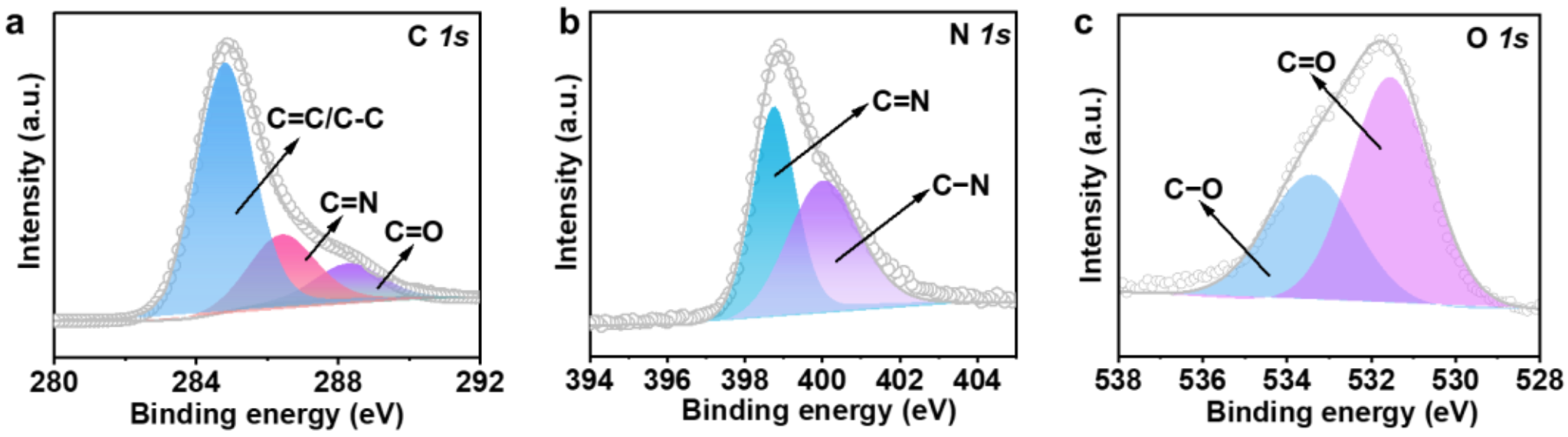

Supplementary Fig. S4 XPS characterization of HPBQ. **a** C *1s*, **b** N *1s*, and **c** O *1s* XPS spectra of HPBQ.

The high-resolution C *1s* spectrum of HPBQ was deconvolved into three characteristic peaks at binding energies of 288.3 eV, 286.5 eV, and 284.8 eV, which are assigned to carbonyl (C=O), imine (C=N), and carbon atoms in the benzene ring skeleton (C=C), respectively (Supplementary Fig. S4a). In the N *1s* spectrum, two predominant peaks located at 398.7 eV and 400.1 eV correspond to C=N and C-N bonds, respectively (Supplementary Fig. S4b). The O *1s* spectrum exhibits two peaks at 531.6 eV and 533.4 eV, which can be attributed to C=O and C–O groups, respectively (Supplementary Fig. S4c).

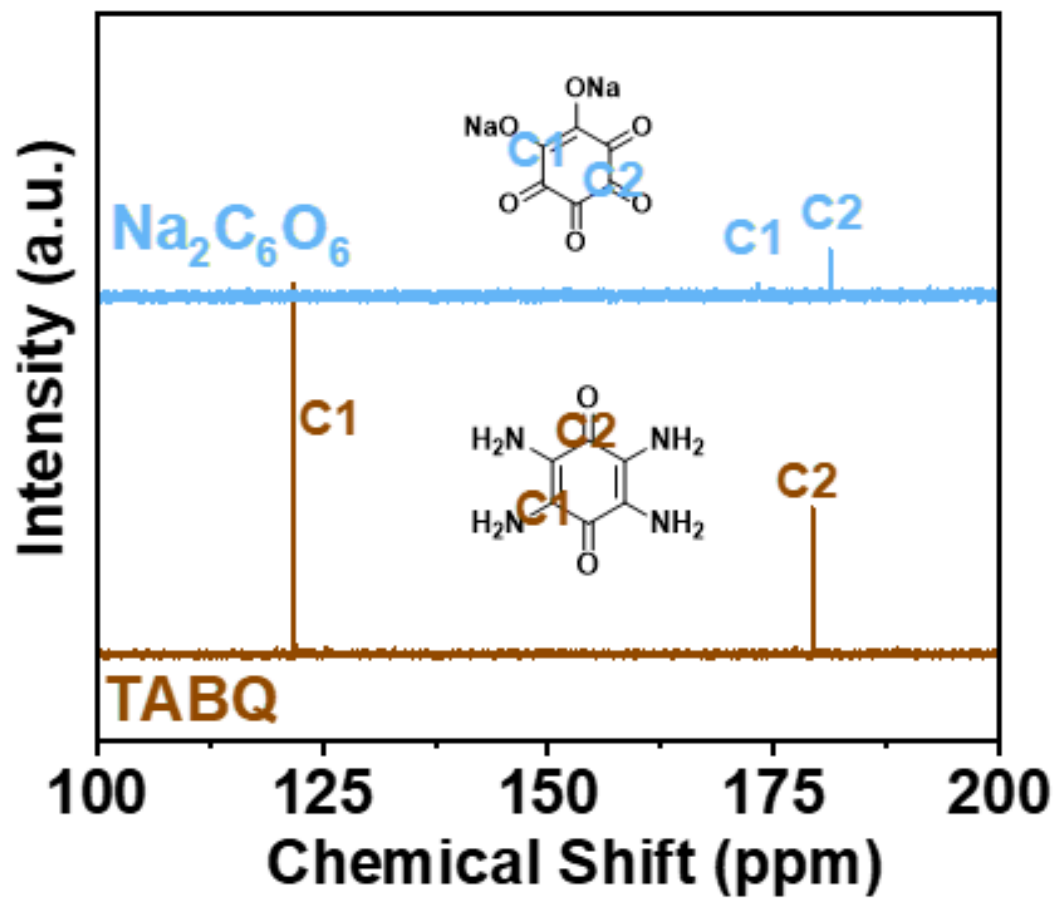

Supplementary Fig. S5 ^{13}C NMR spectra of TABQ and $Na_2C_6O_6$.

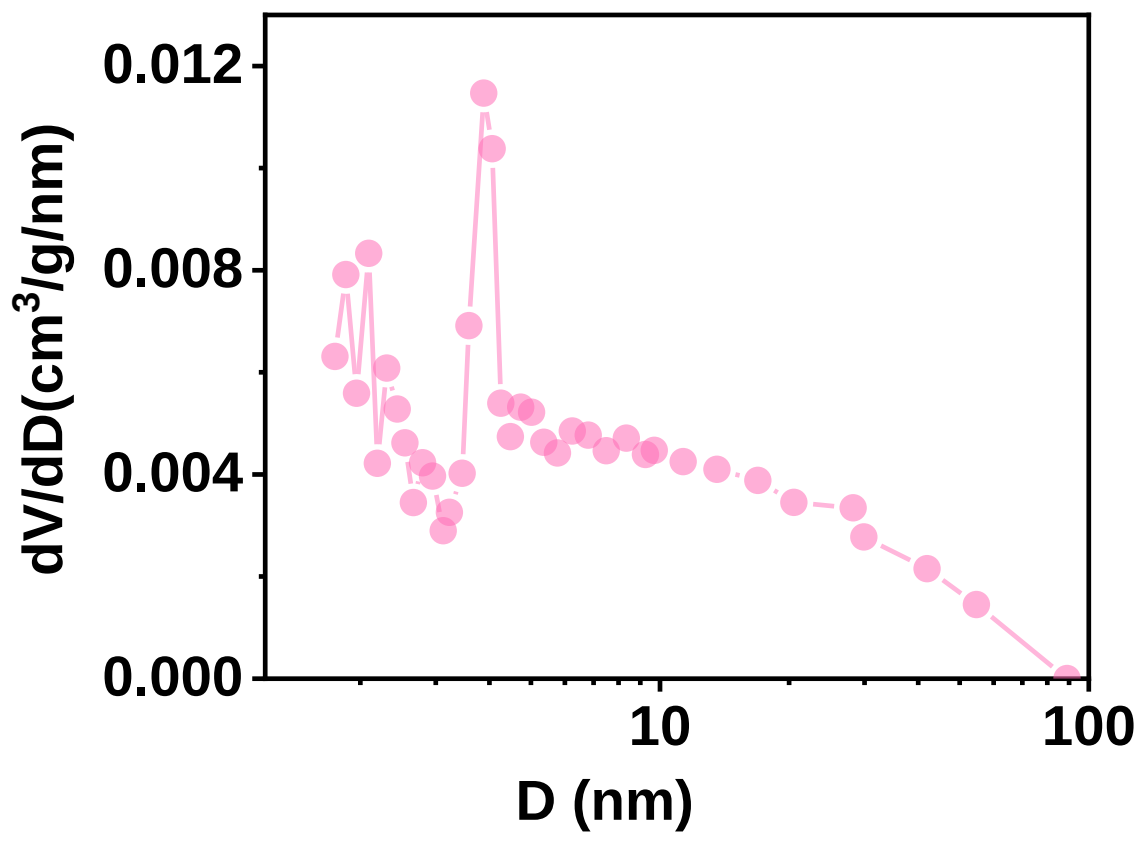

Supplementary Fig. S6 Barrett-Joyner-Halenda (BJH) method analysis of pore size of HPBQ. The result reveals that the pore structure of HPBQ is dominated by mesopores, with negligible contribution from macropores.

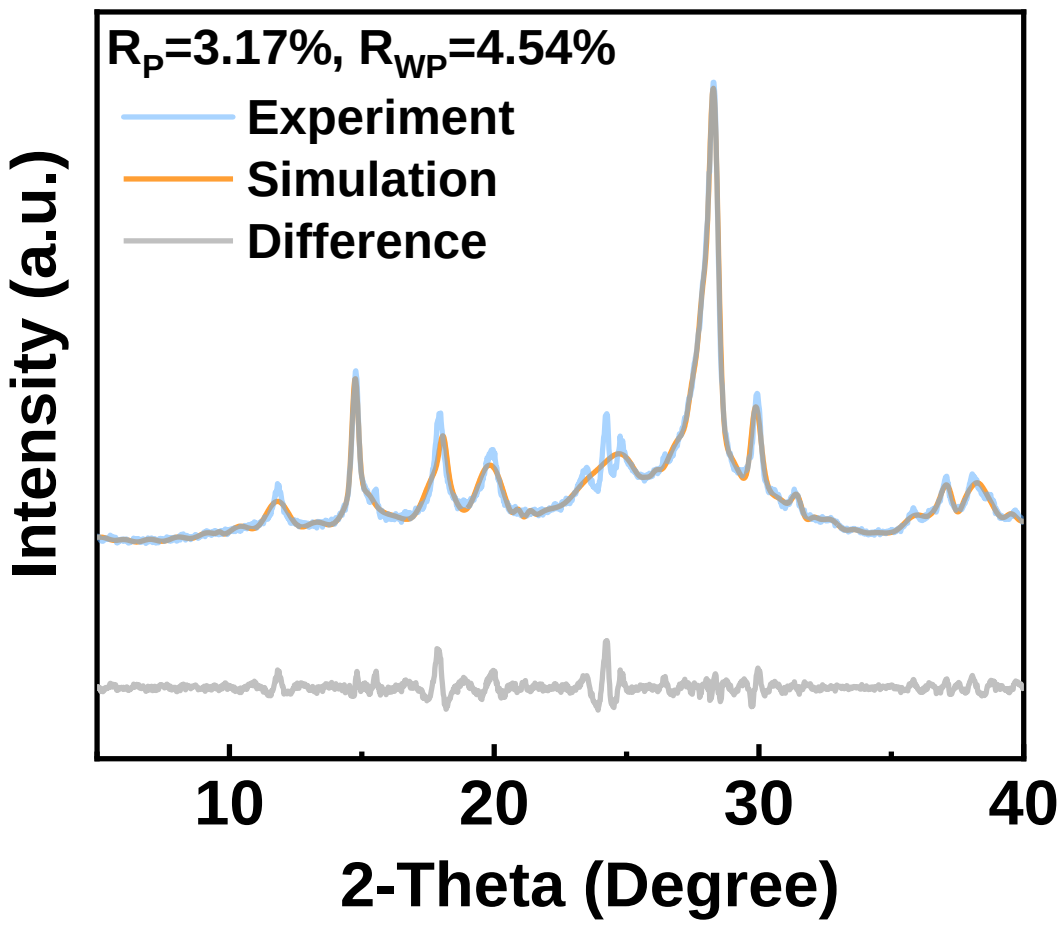

Supplementary Fig. S7 Pawley refinement of the PXRD pattern for HPBQ. The plot shows the observed (blue line), calculated (orange line), and difference (gray line).

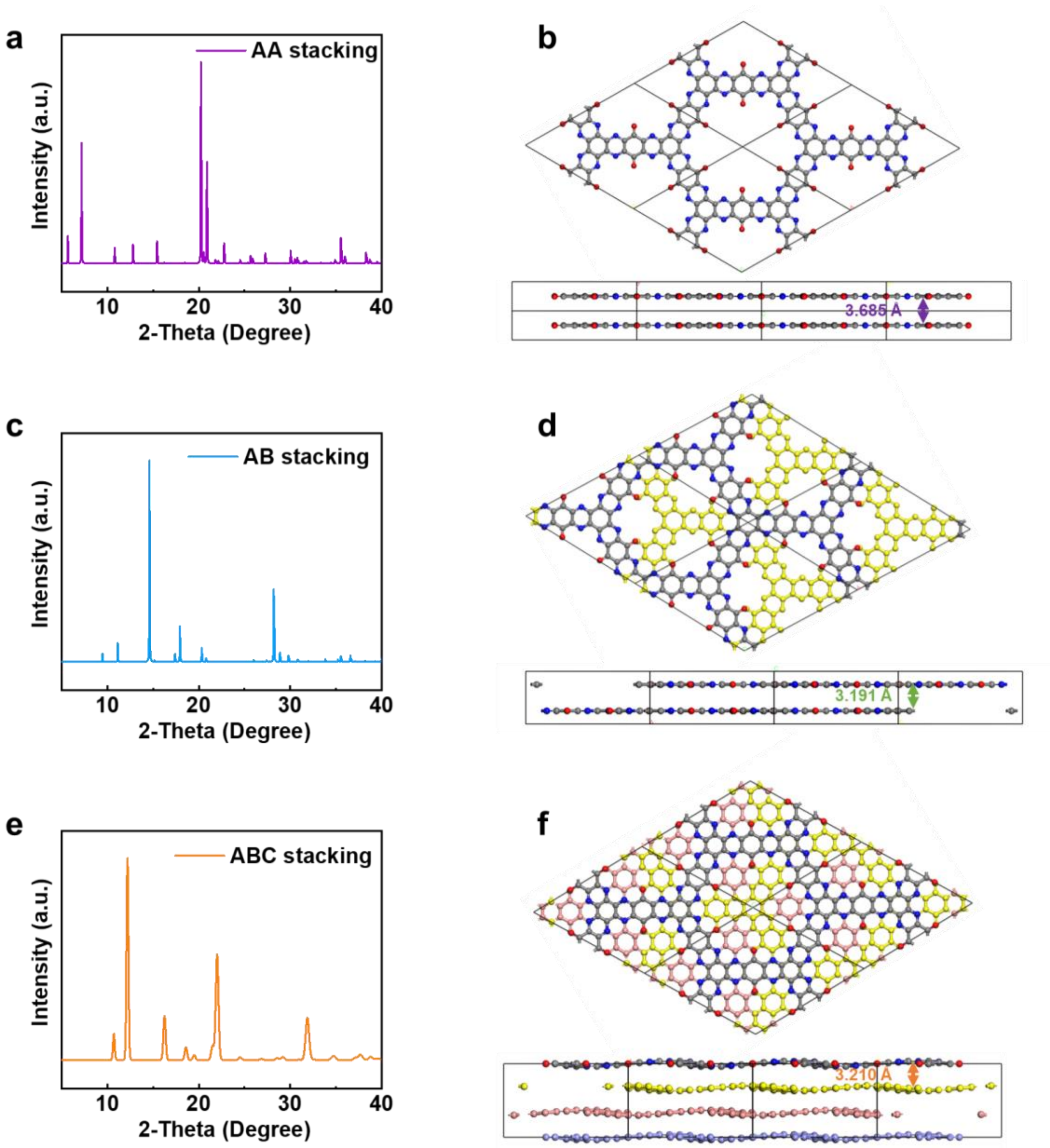

Supplementary Fig. S8 Simulated patterns of three stacking modes (AA, AB, and ABC) of a structurally analogous BQ-COF.[2] **a** Simulated XRD pattern of the AA stacking mode for BQ-COF, **b** Schematic of the simulated AA stacking for BQ-COF, **c** Schematic of the simulated AB stacking for BQ-COF, **d** Structural model of the AB stacking mode for BQ-COF. **e** Schematic of the simulated ABC stacking for BQ-COF, **f** Structural model of the ABC stacking mode for BQ-COF.

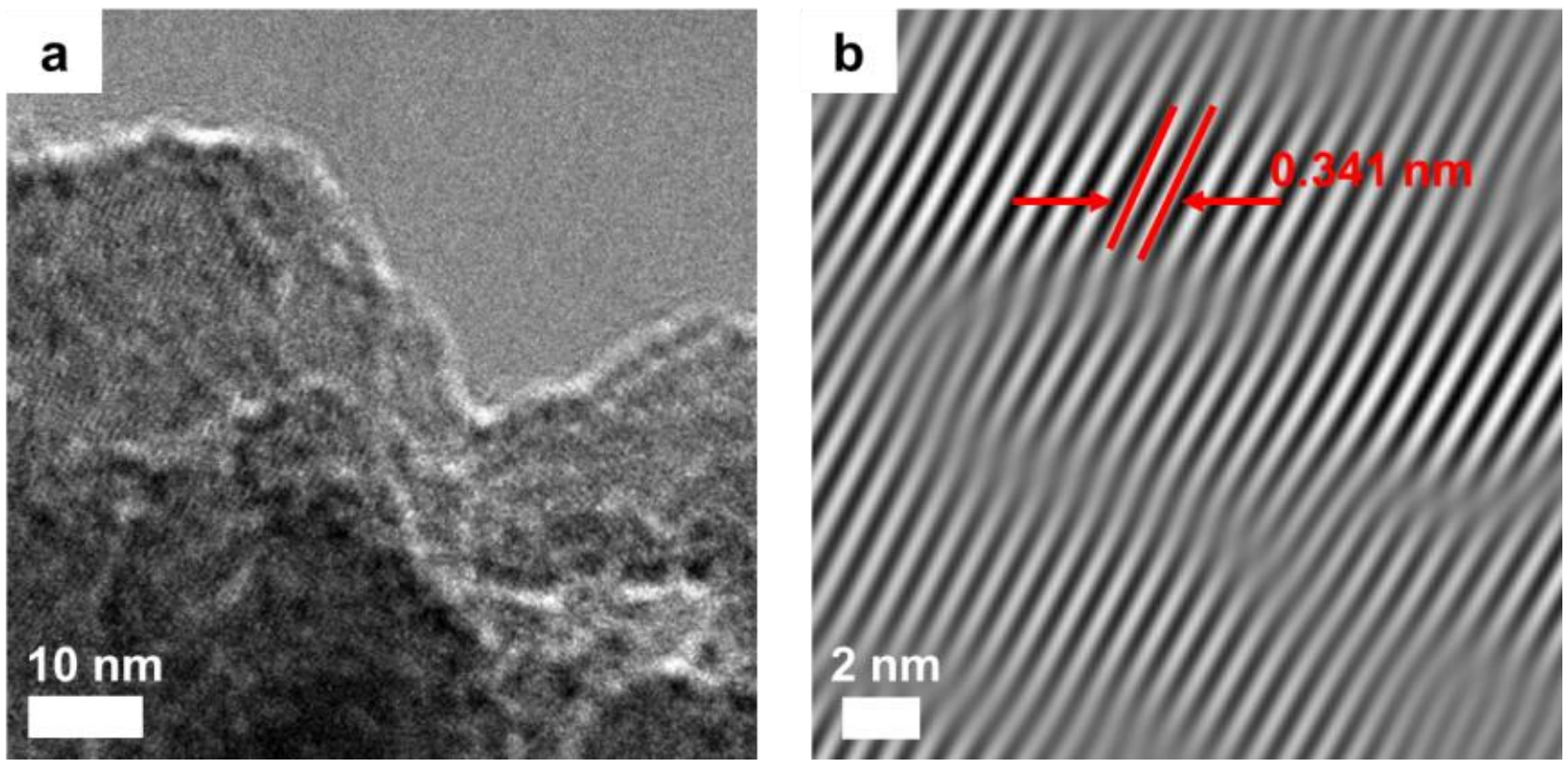

Supplementary Fig. S9 HRTEM imaging and lattice spacing analysis of the HPBQ. **a** HRTEM image of HPBQ and **b** lattice spacing extracted using fast Fourier filtering (FFT).

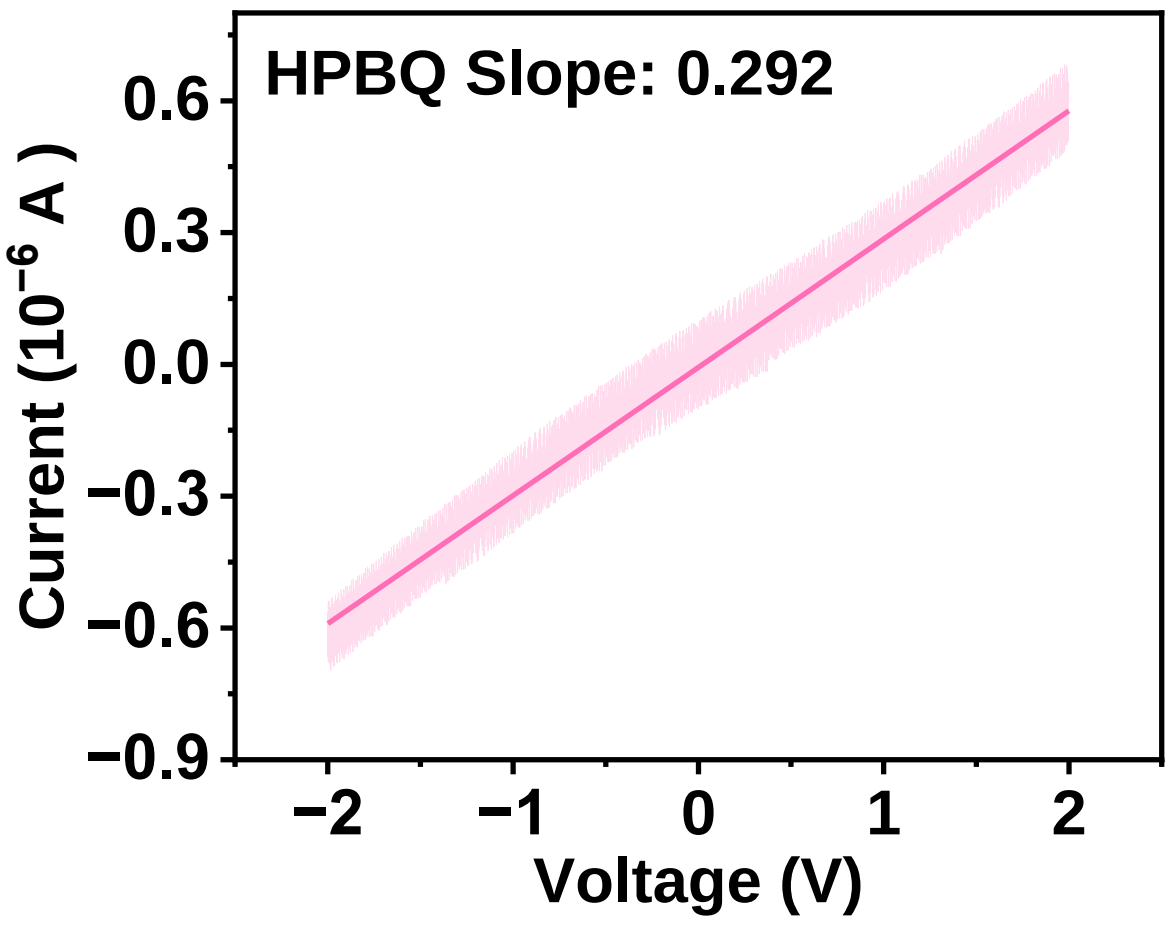

Supplementary Fig. S10 I-V characterization curve of HPBQ. The electrical conductivity of HPBQ was determined by the direct current (DC) polarization method. A pellet of the material was sandwiched between two stainless steel chips and characterized using a Squidstat Plus electrochemical workstation. The transient current through the pellet was measured as a function of voltage. The resistivity (ρ) and conductivity (σ) were calculated from the resulting I-V curve using the following formula:[2]

$$\rho = \frac{V}{I} \times \frac{S}{L} \quad (1)$$

$$\sigma = \frac{1}{\rho} = \frac{I}{V} \times \frac{L}{S} \quad (2)$$

where the V is the applied voltage, I is the measured current density, S is the area of the pellet (0.0000785 m^2), and L is the thickness of the pellet (0.000785 m). The calculated electrical conductivity for HPBQ is 7.03×10^{-7} S cm^{-1}.

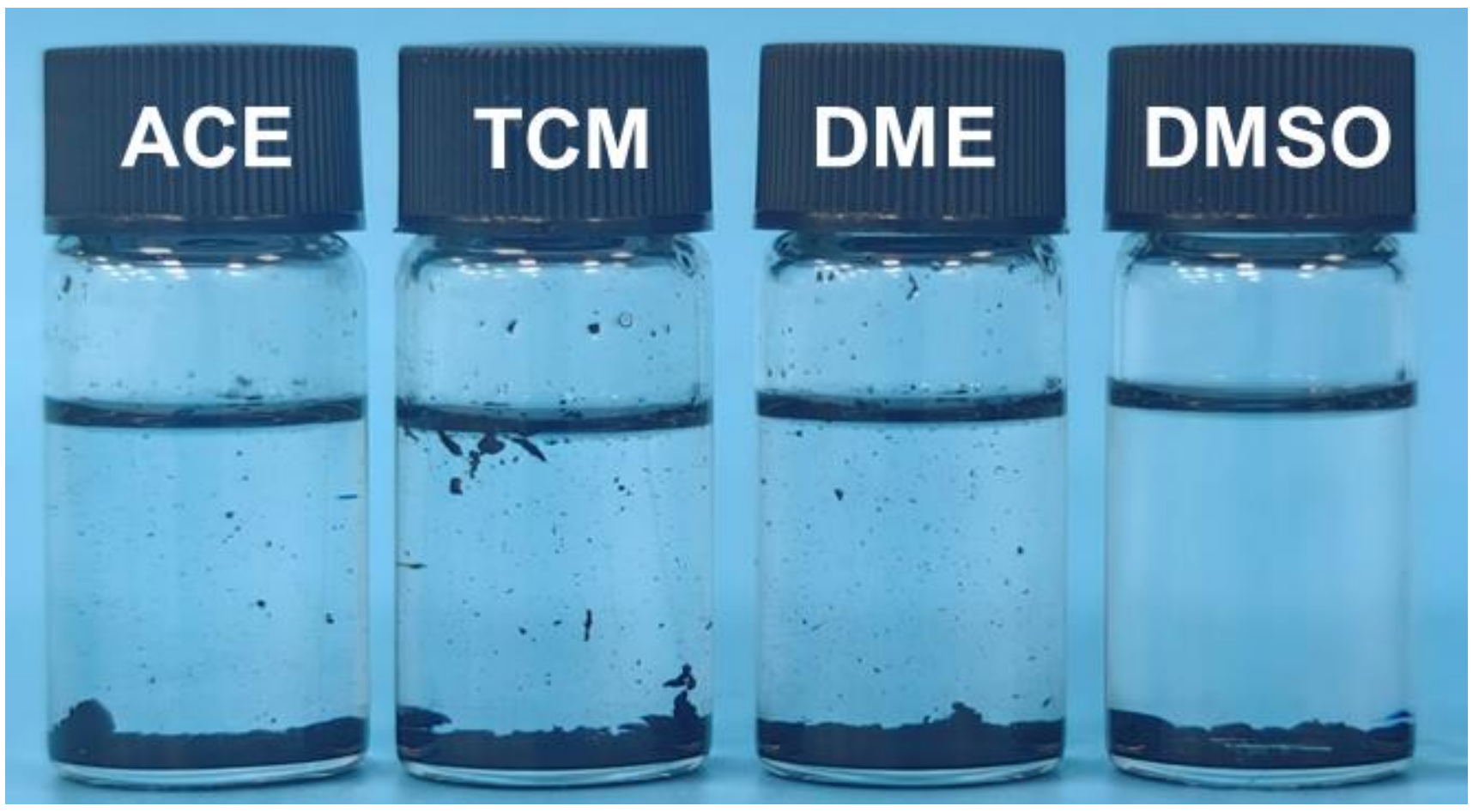

Supplementary Fig. S11 Solubility assessment of HPBQ in various organic solvents. The solubility was assessed by adding 20 mg of HPBQ powder to 3 mL of solvent, including acetone (ACE), trichloromethane (TCM), 1,2-dimethoxyethane (DME), and dimethyl sulfoxide (DMSO), and allowing the mixture to stand for 2 days. HPBQ demonstrates negligible solubility in all tested solvents.

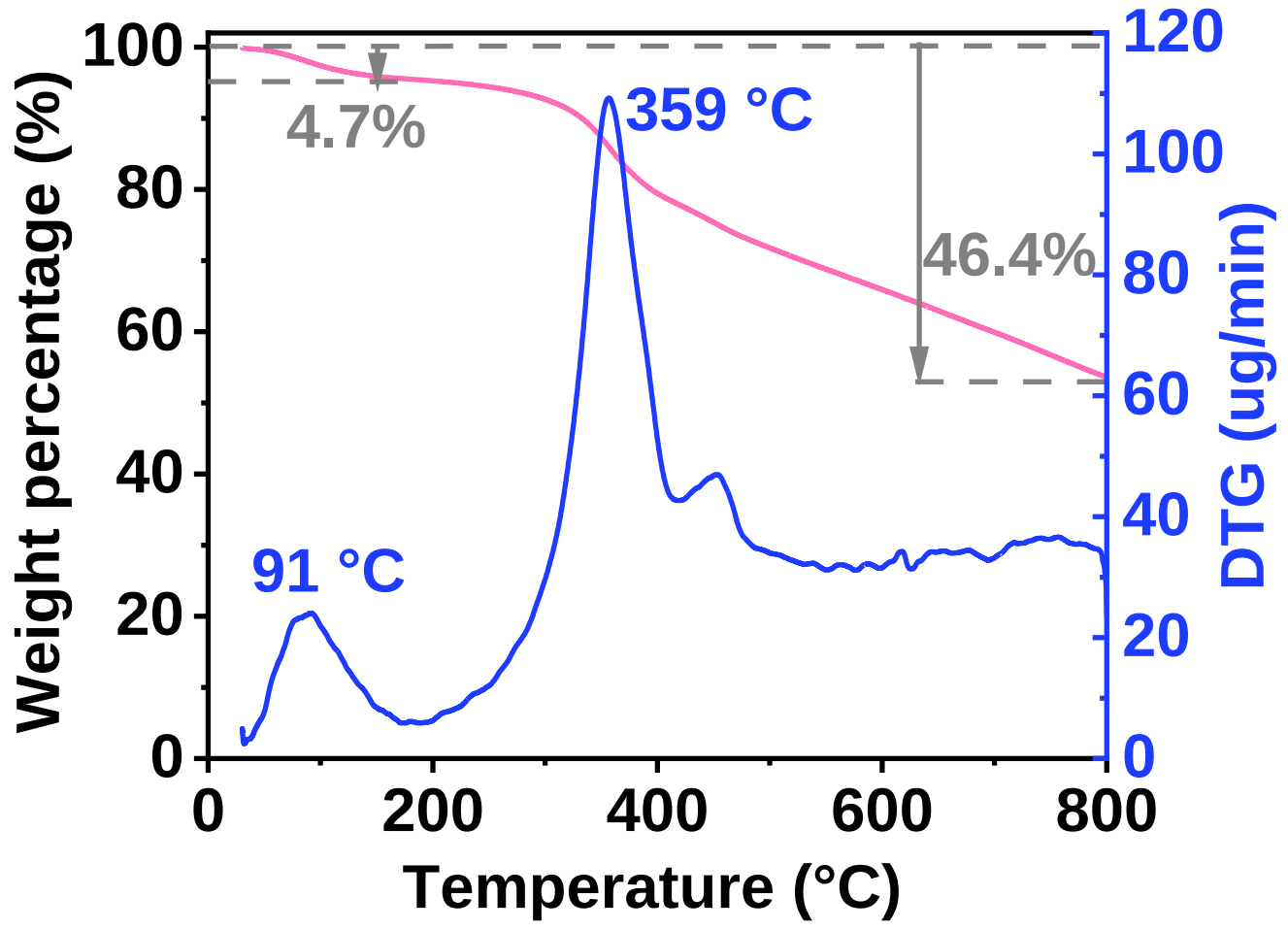

Supplementary Fig. 12 TG curve of HPBQ (atmosphere: argon). HPBQ exhibits two distinct weight loss steps. A weight loss of 4.9% occurs at 91 ℃, primarily attributed to the loss of adsorbed and bound water from the pores. Subsequently, the mass remains almost constant until 359 ℃, demonstrating excellent thermal stability. Beyond this temperature, the mass gradually decreases due to molecular decomposition.

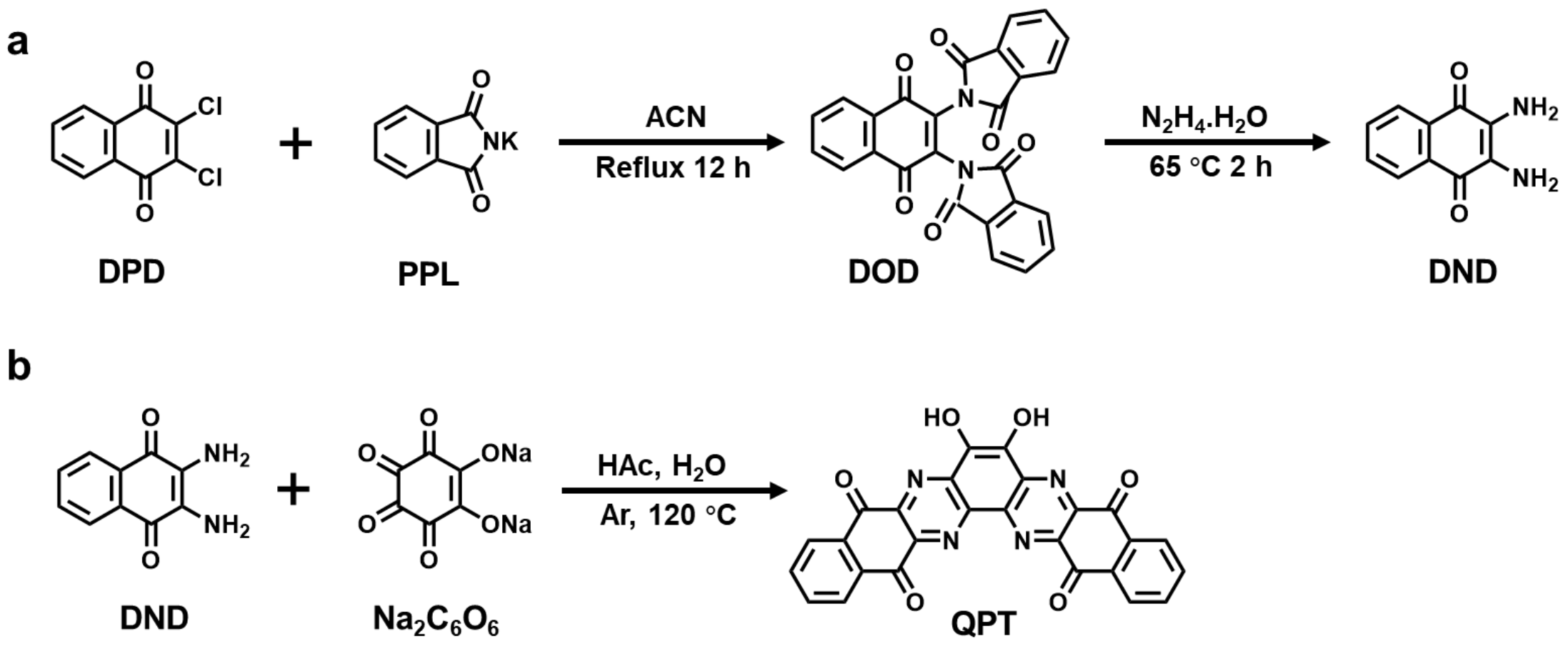

Supplementary Fig. S13 Synthetic routes of **a** DND and **b** QPT.

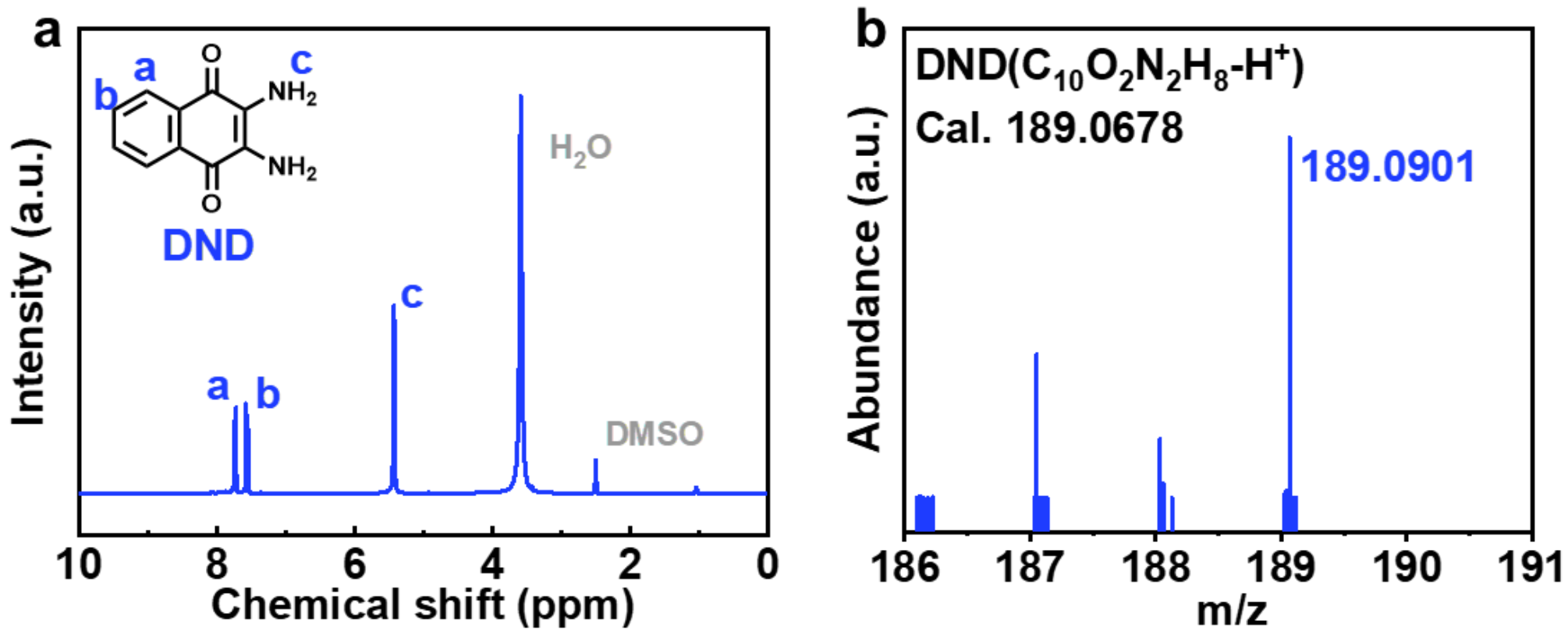

Supplementary Fig. S14 Characterizations of DND. **a** ^{1}H NMR and **b** LC-HRMS of DND

As shown in Supplementary Fig. S14a, the ^{1}H NMR spectrum exhibits three distinct sets of signals at 7.8, 7.6, and 5.5 ppm, with an integrated area ratio of 1:1:2. These signals can be attributed to the protons on the benzene ring and the amino group, respectively, and this ratio is consistent with the expected structure of DND, providing preliminary confirmation of its successful synthesis. Furthermore, LC-HRMS analysis (Supplementary Fig. S14b) reveals a prominent peak at m/z=189.0678 in positive ion mode, which is consistent with the calculated m/z value for protonated DND ($[C_{10}H_8N_2O_2+H]^+$, theoretical m/z =189.0901), thereby confirming the successful synthesis of DND.[3]

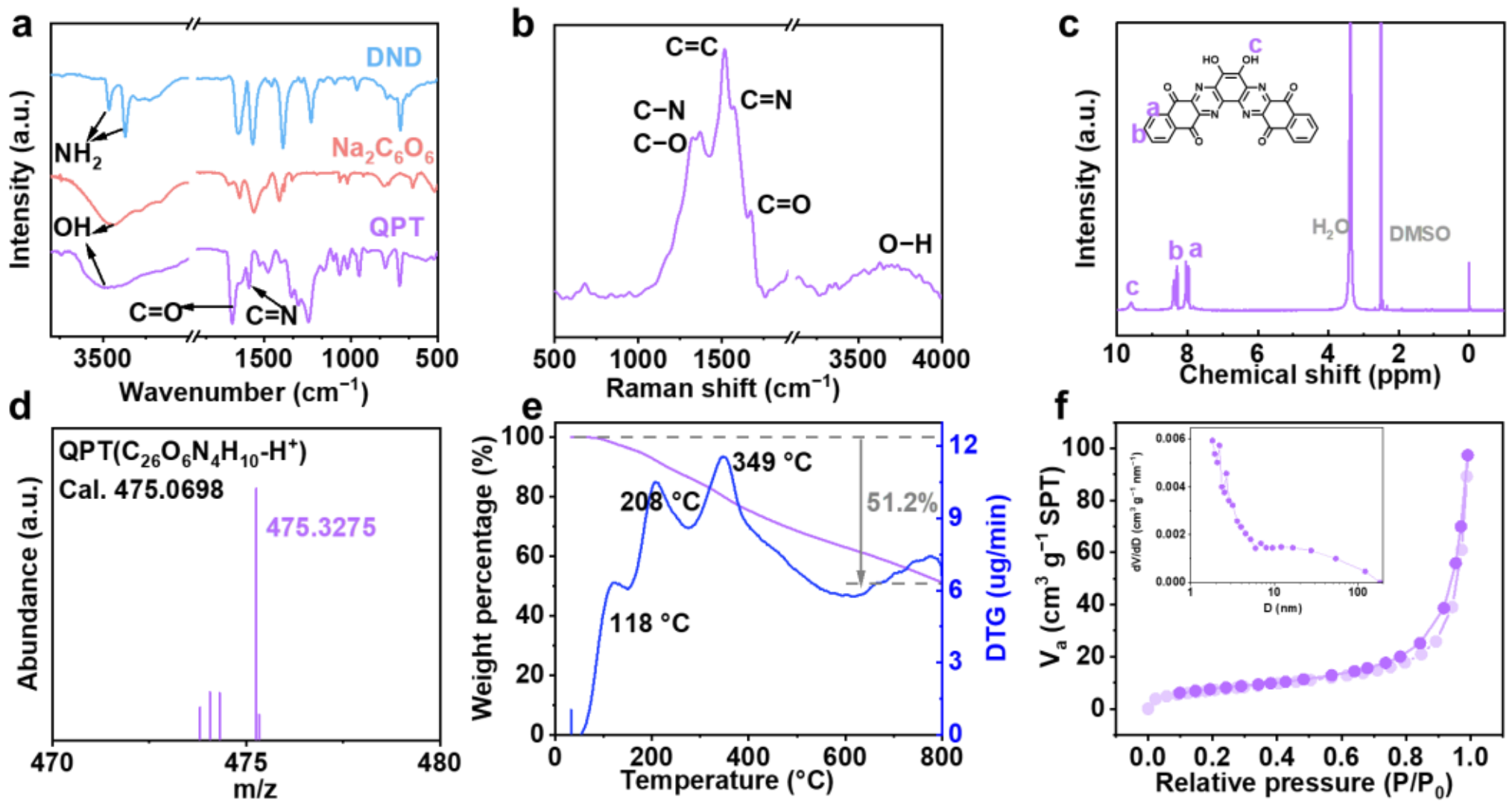

Supplementary Fig. S15 Comprehensive characterizations of QPT. **a** FTIR spectra of DND, $Na_2C_6O_6$, and QPT. **b** Raman spectrum, **c** ^{1}H NMR spectrum, **d** LC-HRMS, **e** TG curve, **f** N_2 adsorption and desorption isotherm (inset is the pore size distribution) of QPT.

A series of characterizations were performed to elucidate the chemical structure, thermal stability, and morphology of QPT. FTIR spectra (Supplementary Fig. S15a) revealed the complete consumption of amino groups, as evidenced by the disappearance of the characteristic diamine peak of TABQ at 3373 cm^{-1}. Notably, QPT exhibited distinct vibrational bands corresponding to hydroxyl (–OH, 3483 cm^{-1}) and carbonyl (C=O, 1680 cm^{-1}) functionalities, alongside newly emerged peaks assignable to C=N (1582 cm^{-1}) and C–N (1338 cm^{-1}) bonds, which collectively corroborated the proposed molecular structure of QPT. This structural assignment was further confirmed by Raman spectroscopy (Supplementary Fig. S15b), where prominent scattering bands for O–H (3813 cm^{-1}), C=N (1588 cm^{-1}), C–N (1312 cm^{-1}), and C=O (1676 cm^{-1}) were observed. ^{1}H NMR analysis (Supplementary Fig. S15c) provided additional structural validation: While the precursor DND displayed characteristic signals for C–NH_2 at 5.6 ppm and aromatic protons (Ar–H) at 7.8–7.9 ppm, these amine protons were absent in QPT. Instead, QPT exhibited Ar–H resonances at 7.9–8.1 ppm and a hydroxyl proton peak at 9.5 ppm, fully consistent with its designed structure. LC–HRMS,

(Supplementary Fig. S15d) shows a peak at m/z=475.3275 ($[M–H]^+$), matching the calculated mass of QPT (M =475.0698). The collective analysis of these spectroscopic changes confirms the successful synthesis of QPT. TGA (Supplementary Figure S15e) shows that QPT has multiple distinct weight loss points, with weight loss starting at 118 ℃, which may be attributed to the loss of intramolecular crystalline water. Subsequently, weight loss points appear at 208 ℃ and 349 ℃, indicating that QPT has relatively poor thermal stability. The specific surface area of QPT was determined to be 27.1 m^2 g^{-1} (Supplementary Figure S15f). Pore size distribution analysis reveals the presence of mesopores (1.8 nm) and micropores (2.2 nm), which arise from the voids formed inside or between particles due to the stacking, or aggregation of QPT in the solid state.

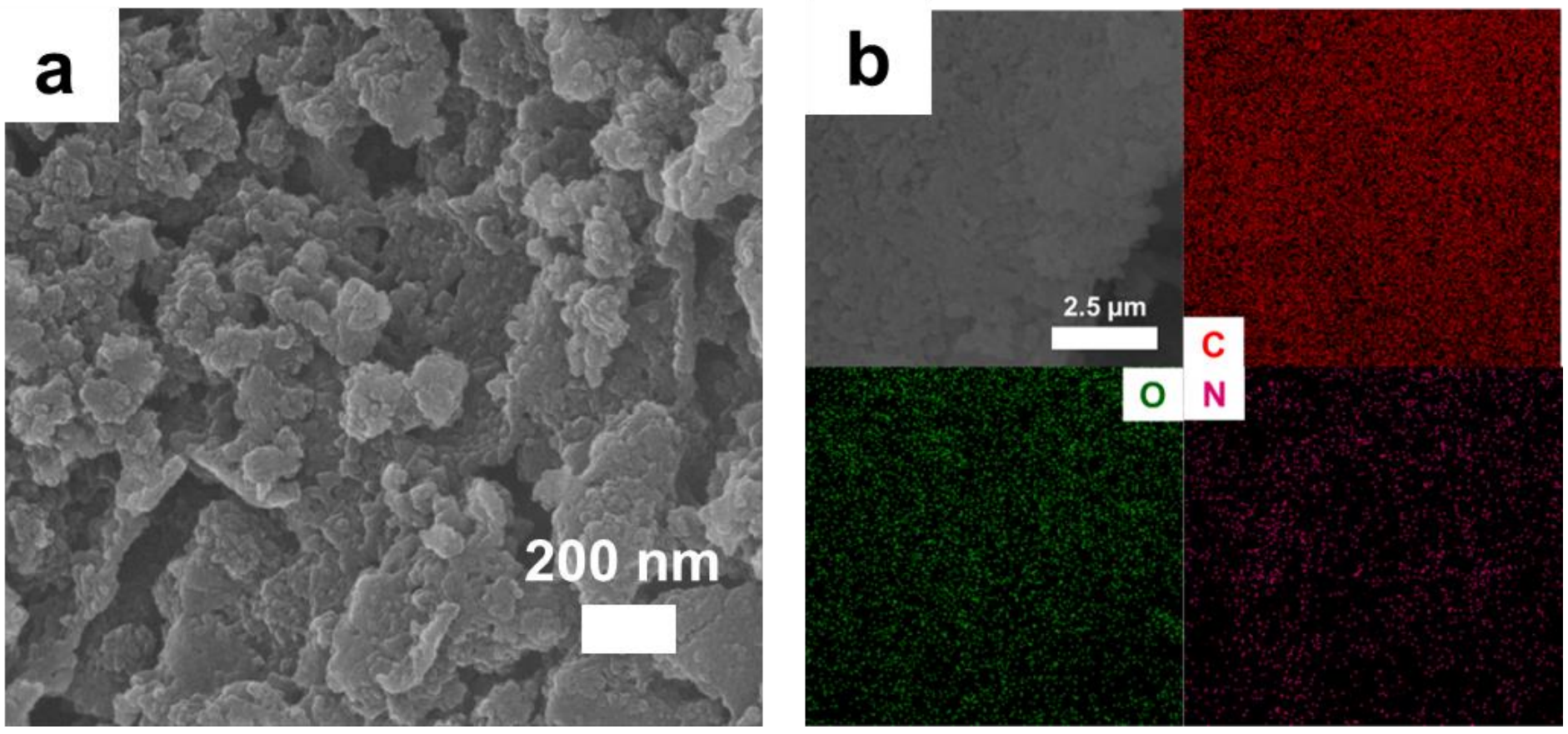

Supplementary Fig. S16 Morphology and elemental composition of QPT. **a** SEM image and **b** EDS elemental mapping of QPT. Scanning electron microscopy revealed uniformly distributed nanoparticles (Supplementary Fig. S16a), while EDS elemental mapping (Supplementary Fig. S16b) confirmed the predominant composition of C, N, and O elements.

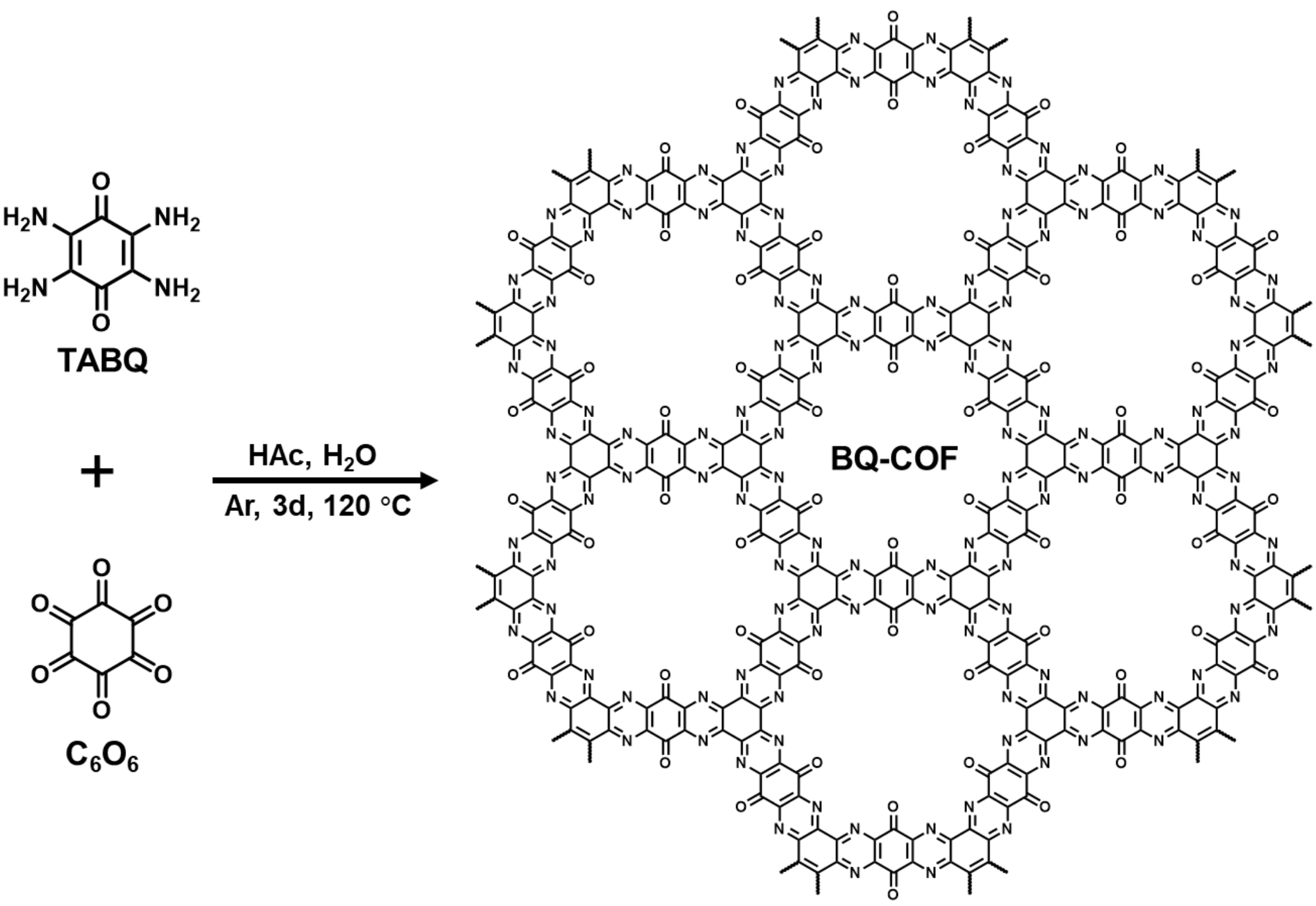

Supplementary Fig. S17 Synthetic route of BQ-COF.

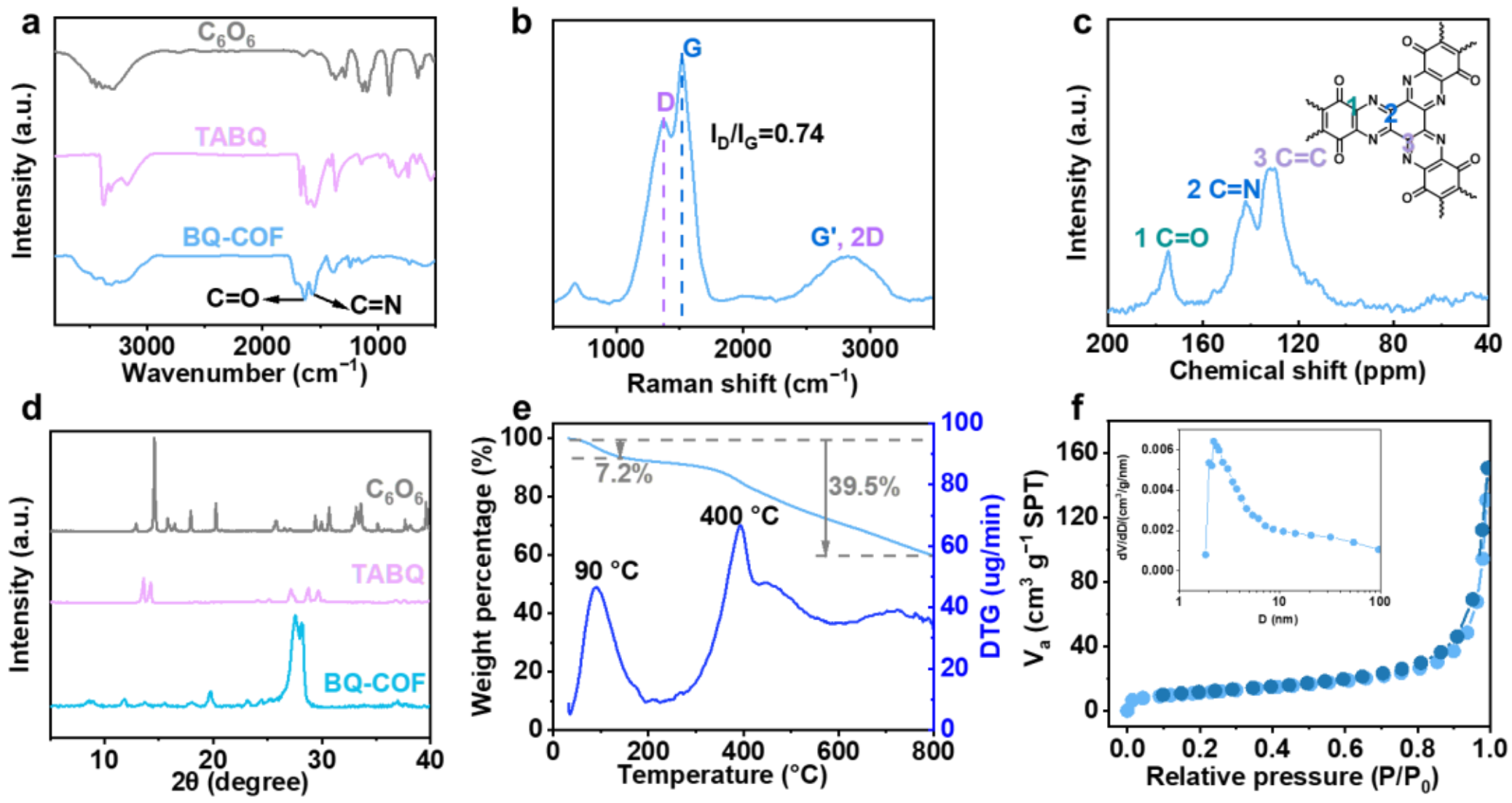

Supplementary Fig. S18 Comprehensive characterizations of BQ-COF. **a** FT-IR spectra of BQ-COF, TABQ, and C_6O_6. **b** Raman spectrum and **c** solid-state ^{13}C NMR spectrum of BQ-COF, **d** PXRD pattern of BQ-COF, TABQ, and C_6O_6, **e** TG curve, and **f** N_2 adsorption and desorption isotherm (inset is the pore size distribution.) of BQ-COF.

The FTIR spectrum of BQ-COF (Supplementary Fig. S18a) displays two distinct absorption bands at 1627 cm^{-1} and 1545 cm^{-1}, assignable to the stretching vibrations of carbonyl (C=O) and imine (C=N) groups, respectively. Raman spectroscopy reveals that BQ-COF exhibits distinct D and G bands, with an I_D/I_G ratio of 0.84 (Supplementary Fig. S18b). According to a previous report,[4] BQ-COF is a two-dimensional structure with a moderate level of defects. Solid-state ^{13}C NMR spectroscopy (Supplementary Fig. S18c) further confirms the structure, with all observed peaks corresponding to labeled functional groups in the proposed framework. Specifically, resonances at approximately 170 ppm and 143 ppm are attributed to the carbonyl carbon and the imine linkage formed via Schiff base condensation, respectively. The crystallinity of BQ-COF was examined by PXRD (Supplementary Fig. S18d).[5] The observed pattern shows a strong peak at 27.7 °, which is assignable to the interlayer π–π stacking. TGA (Supplementary Fig. S18e) shows that BQ-COF has two distinct weight loss points. The weight loss at 90 ℃ is primarily due to the loss of

crystalline water and solvent within the molecule, with a weight loss rate of 7.2%. No further weight loss is observed before 400 ℃, indicating that BQ-COF possesses excellent thermal stability. The BET surface area of BQ-COF was measured to be 40.4 m^2 g^{-1} (Supplementary Fig. S18f), and pore size distribution analysis indicates that the pore structure of this material is predominantly microporous (~2.2 nm). This relative low surface area likes arise from (1) the inherent limitations of its two-dimensional layered structure, along with the influence of the interlayer stacking pattern on pore connectivity; and (2) the limited reversibility of the Schiff base reaction, which can increase the amorphous components during crystallization under different synthesis conditions.[6] Therefore, in previously reported studies, the specific surface area of a given COF varies with synthesis conditions. In fact, the specific surface area of BQ-COF obtained in this study falls within the range of those reported in the literature.[4]

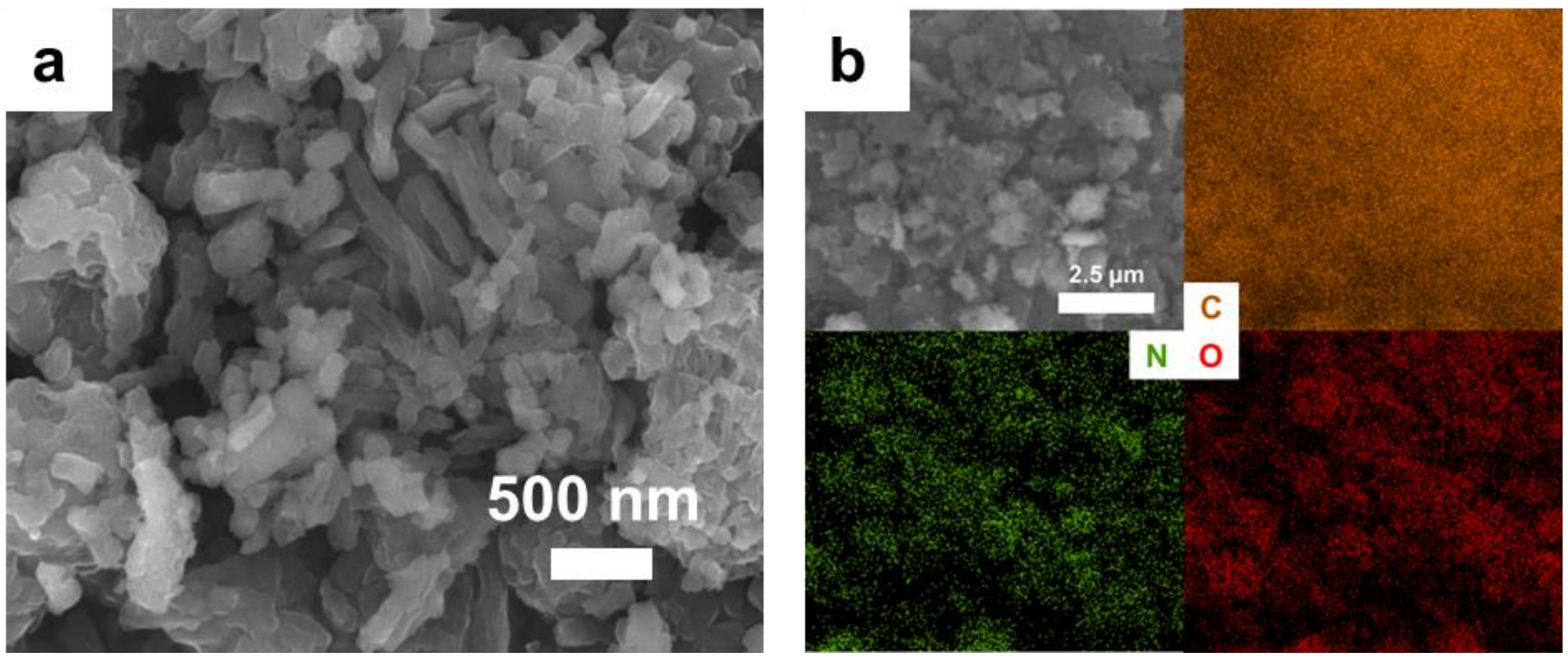

Supplementary Fig. S19 Morphology and elemental distribution of BQ-COF. **a** SEM image and **b** EDS elemental mapping of BQ-COF. SEM image reveals nanorod-like morphologies (Supplementary Fig. S19a), while EDS (Supplementary Fig. S19b) elemental mapping demonstrates spatially uniform distribution of C, N, and O throughout the BQ-COF structure.

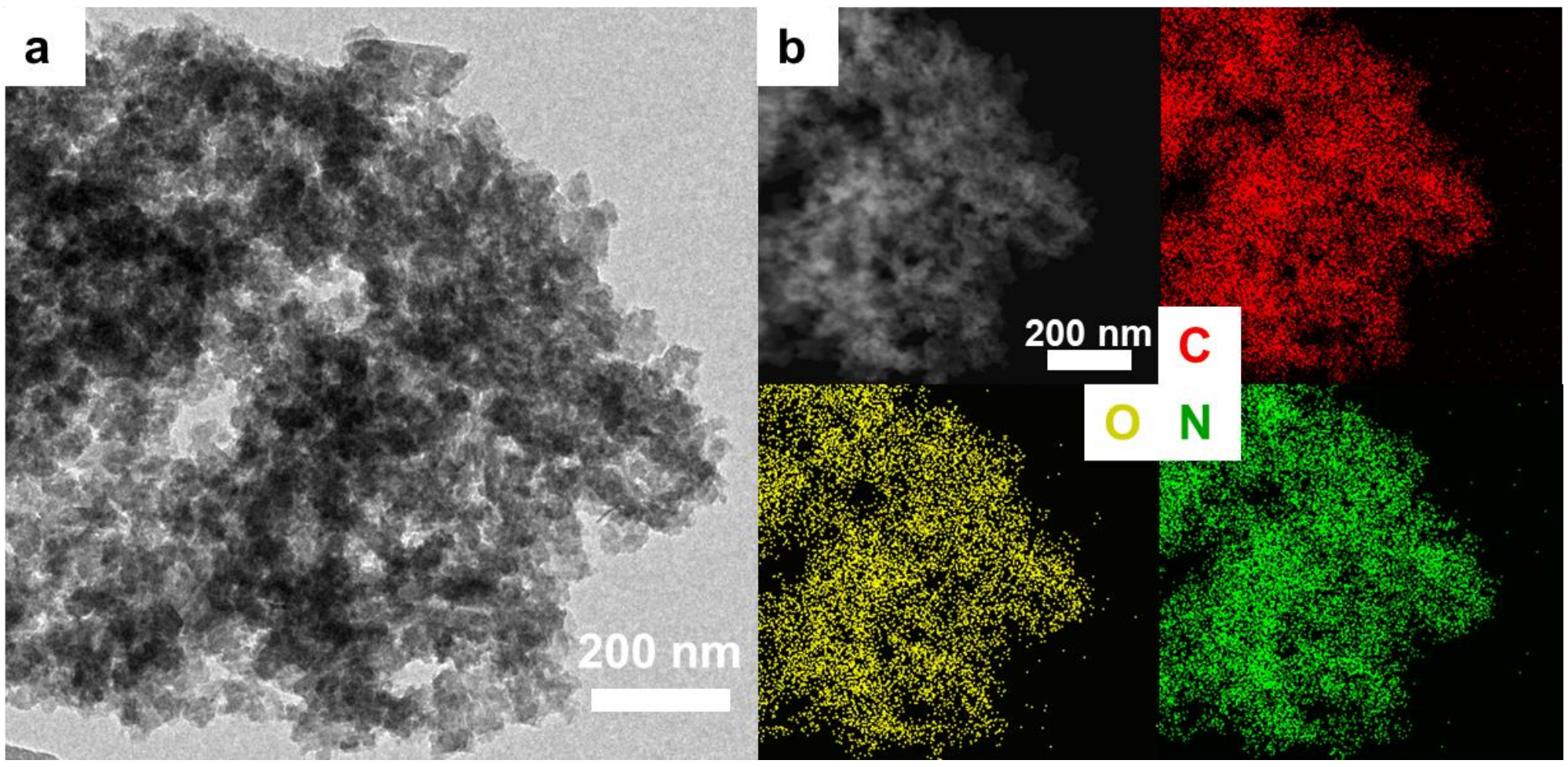

Supplementary Fig. S20 Morphology and elemental mapping of HPBQ. **a** TEM image and **b** EDS elemental mapping of BQ-COF.

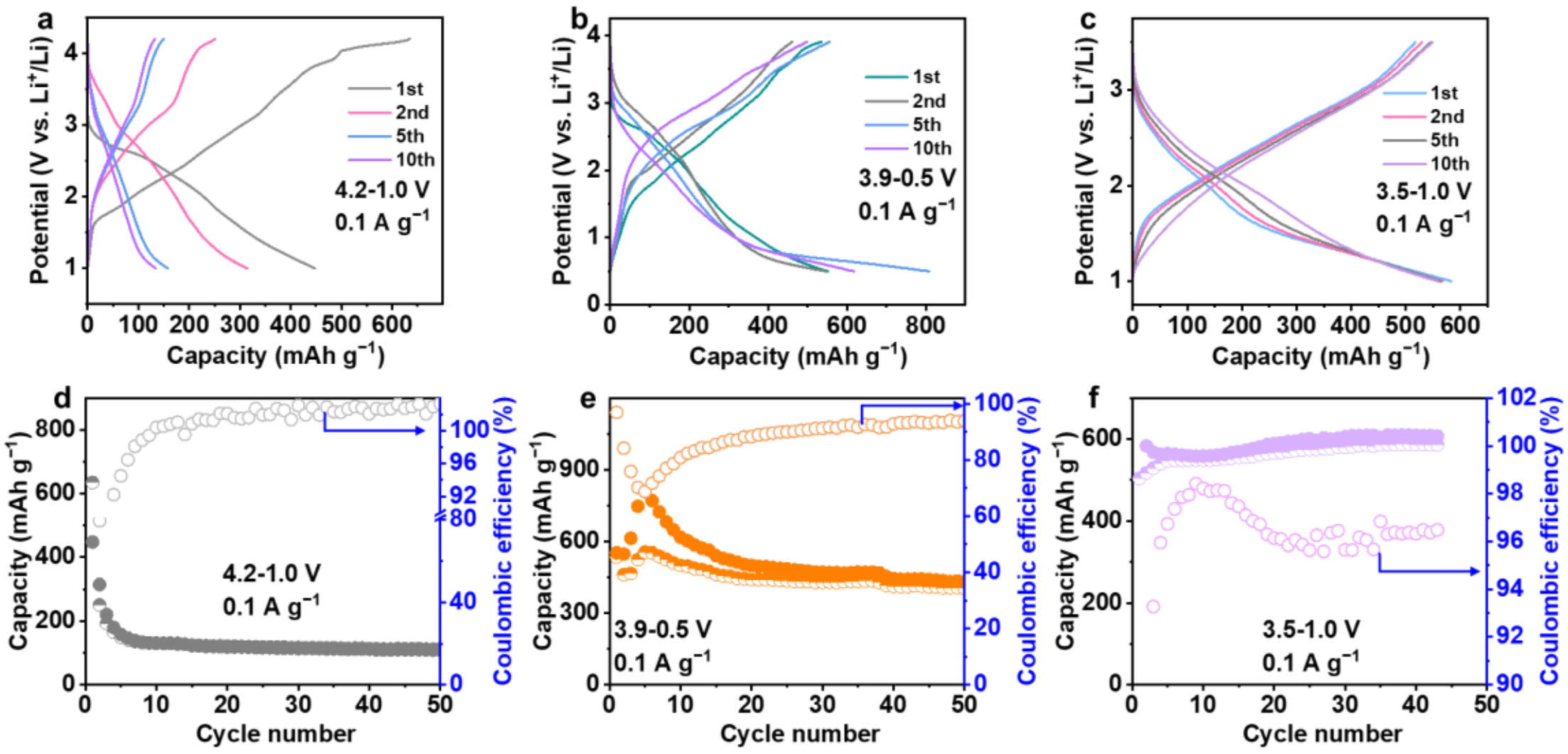

Supplementary Fig. S21 Electrochemical performance of HPBQ across different voltage windows. **a-c** Galvanostatic charge/discharge (GCD) profiles and **d-f** corresponding cycling stability of HPBQ evaluated in different voltage ranges: **a,d** 4.2–1.0 V, **b,e** 3.9–0.5 V, and **c,f** 3.5–1.0 V.

Within the voltage window of 4.2-1.0 V, HPBQ exhibits an initial discharge capacity of 448 mAh g^{-1} and an initial charge capacity of 637 mAh g^{-1} (Supplementary Fig. S21a), with an additional charging plateau appearing at 4.0 V compared to that observed in the 3.9-1.0 V window (Fig. 2a). This plateau should be attributed to the electrolyte decomposition, which is detrimental for cycle stability.[7] Consequently, only 17% of the initial capacity is retained after 50 cycles at 0.1 A g^{-1} (Supplementary Fig. S21d).

When the voltage window was changed to 3.9–0.5 V, HPBQ delivered an initial discharge capacity of 807 mAh g^{-1} and an initial charge capacity of 554 mAh g^{-1} (Supplementary Fig. S21b). A long tail was observed in the initial discharge curve, which should be attributed to the irreversible decomposition of the DOL/DME solvent and LiTFSI salt at low potentials.[8] The capacity retention after 50 cycles was only 54% (Supplementary Fig. S21e), likely due to the decomposition of the electrolyte that consumes lithium ions and contaminates the electrode interface,[9] and/or to the excessively low discharge voltage that may lead to irreversible structural degradation of HPBQ induced by deep lithiation.[10]

Notably, HPBQ demonstrates excellent cycling stability within the 3.5–1.0 V voltage window, but the reversible capacity is only 585 mAh g^{-1} (Supplementary Fig. S21c,f). This is because the electrochemical processes occurring within this voltage range do not involve the hydroxy-carbonyl redox conversion, which is essential for achieving fully utilization of the active sites in HPBQ. Therefore, the 3.9–1.0 V window was ultimately selected as the operating voltage range, as it enables both near-complete active sites utilization and good cycling stability (Fig. 2a).

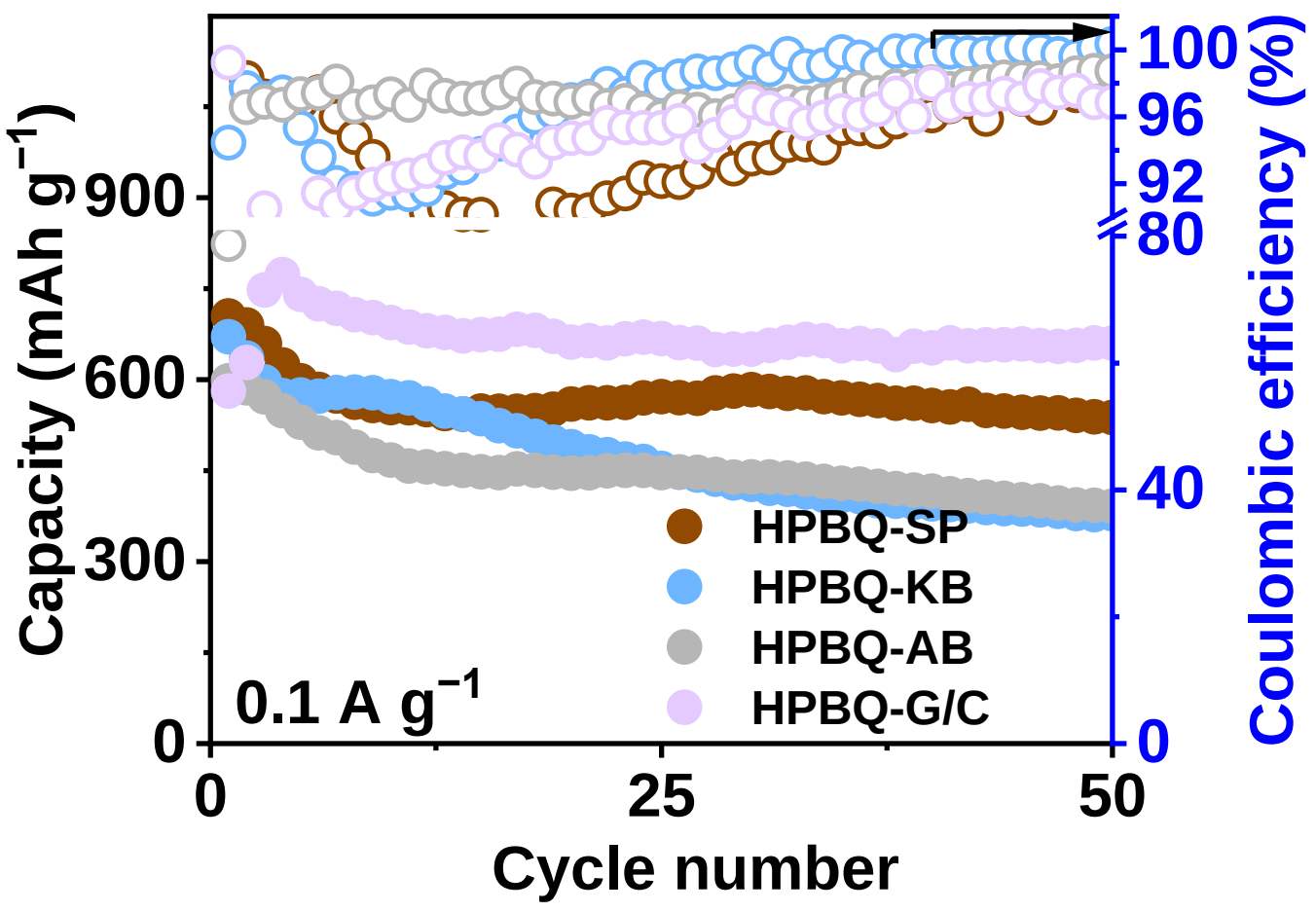

Supplementary Fig. S22 Cycling performance of HPBQ with different types of conductive additives at 0.1 A g^{-1}.

To select suitable conductive carbon, we evaluated the cycling stability of HPBQ with different carbon materials, including a mixture of graphene oxide and carbon nanotube (1:1 mass ratio, G/C), acetylene black (AB), Ketjen black (KB), and Super P (SP). As shown in Supplementary Fig. S22, after 50 cycles, the capacity retention rates of HPBQ electrodes using SP, KB, AB, and G/C were 78%, 58%, 66%, and 86%, respectively. The superior stability of the G/C-based electrode can be attributed to the synergistic effect of GO and CNT in constructing a highly conductive network, as well as the strong π-π interactions formed with HPBQ molecules. These features effectively suppress the dissolution of the active material in the electrolyte, thereby enhancing cycling stability.

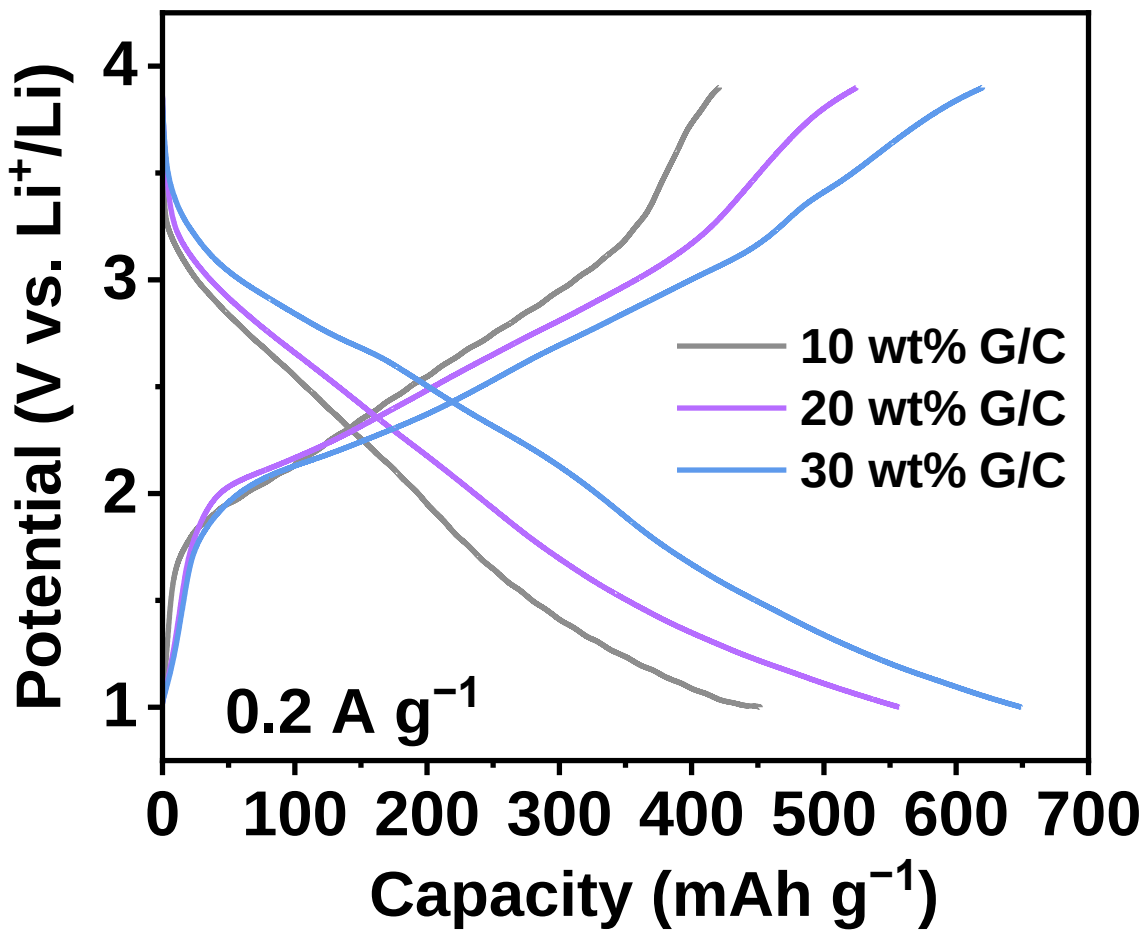

Supplementary Fig. S23 GCD profiles of HPBQ with varying carbon contents (G/C as the conductive carbon) at 0.2 A g^{-1}.

Regarding the influence of carbon content, we found that appropriately increasing the amount of conductive carbon enhances the reversible capacity of the HPBQ electrode. This improvement is primarily attributed to the formation of a more efficient electron-conducting network at higher carbon ratios, which effectively reduces the charge-transfer resistance of the electrode and promotes the full utilization of the active material, thereby increasing the reversible capacity. However, an excessively high carbon ratio would reduce the overall energy density of the electrode. Therefore, we optimized the carbon content to 30 wt.%, which ensures high electronic conductivity while maintaining a reasonable electrode energy density.

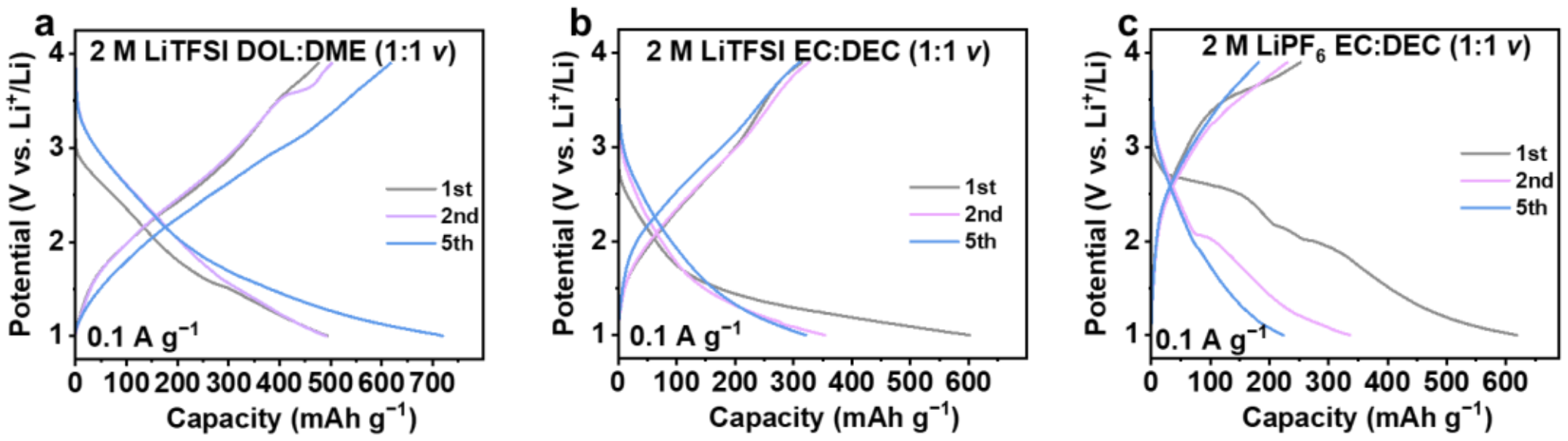

Supplementary Fig. S24 GCD profiles of HPBQ employing **a** 2 M LiTFSI (DOL/DME), **b** 2 M LiTFSI (EC/DEC) and **c** 2 M $LiPF_6$ (EC/DEC) electrolytes at 0.1 A g^{-1}.

As shown in Supplementary Fig. S24a, the HPBQ electrode in the 2 M LiTFSI DOL/DME achieved a stable capacity of 723 mAh g^{-1} after five cycles with an average voltage of approximately 1.85 V. In contrast, HPBQ in the 2 M LiTFSI EC/DEC suffered from a capacity decay from 603 at the 1st cycle to 331 mAh g^{-1} at the 5th cycles, accompanied by a drop in average discharging voltage to ~1.55 V (Supplementary Fig. S24b). This performance degradation is possibly attributed to active material dissolution and Al corrosion induced by LiTFSI in carbonates[11]. In addition, using $LiPF_6$ in DOL/DME lead to electrolyte gelation,[12] while $LiPF_6$ in EC/DEC gave a high initial capacity (621 mAh g^{-1}) and elevated discharge voltage (2.0 V) (Supplementary Fig. S24c). This enhanced performance may be attributed to the formation of a conductive inorganic-rich electrode-electrolyte interphase derived from the decomposition of $LiPF_6$.[13] However, the cycle stability of this system is unsatisfactory, with the capacity fading to 238 mAh g^{-1} after just five cycles. Based on this systematic screening, 2 M LiTFSI in DOL/DME was ultimately selected as the electrolyte for subsequent investigations.

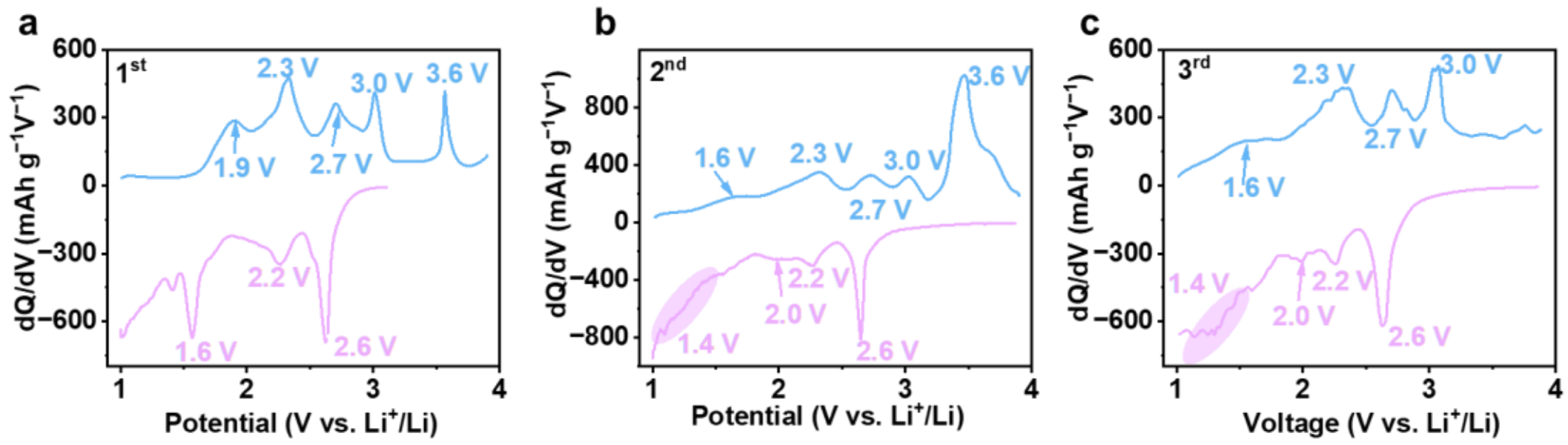

Supplementary Fig. S25 Differential capacity (dQ/dV) profiles of HPBQ during the **a** 1st , **b** 2nd, and **c** 3rd cycles. The results reveal a distinct additional peak at approximately 3.6 V during charging in the first two cycles, which disappears in the third cycle. Furthermore, HPBQ exhibits four pairs of reversible redox peaks at ~2.6 V/~3.0 V, ~2.2 V/~2.7 V, ~2.0 V/~2.3 V, and ~1.4 V/~1.6 V, suggesting a multi-stage lithium intercalation process.

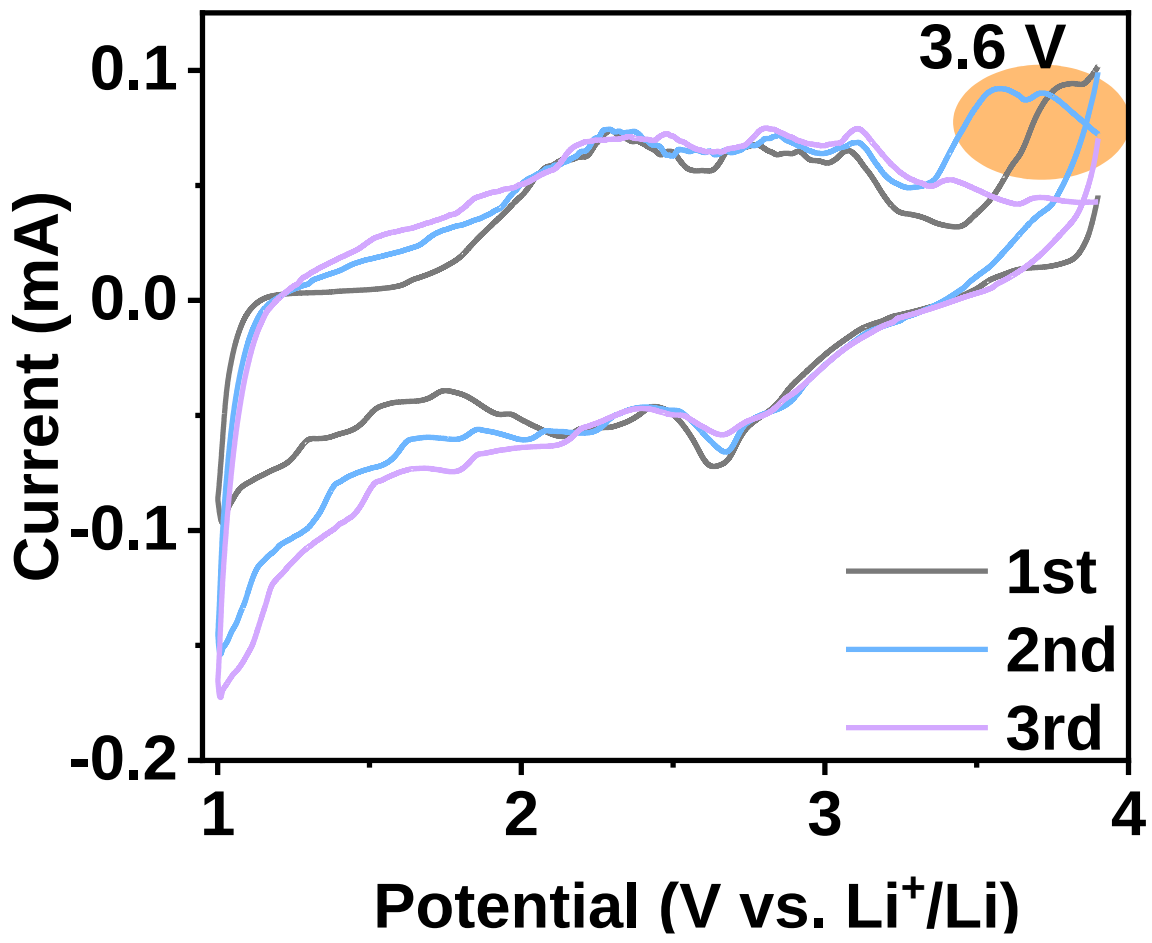

Supplementary Fig. S26 CV curves of the HPBQ electrode at a scan rate of 0.2 mV s^{-1}. During the initial two charging cycles, a distinct additional oxidation peak emerges at approximately 3.5 V. The area of this peak increases in the subsequent scan, and eventually disappears by the third cycle, which is consistent with the dQ/dV curve results (Supplementary Fig. S26). This peak corresponds to the conversion of hydroxyl groups to carbonyl groups.[14,15] Furthermore, multiple pairs of reversible redox peaks are observed, indicating a multi-stage and continuous Li^+ insertion/extraction process in the HPBQ electrode.

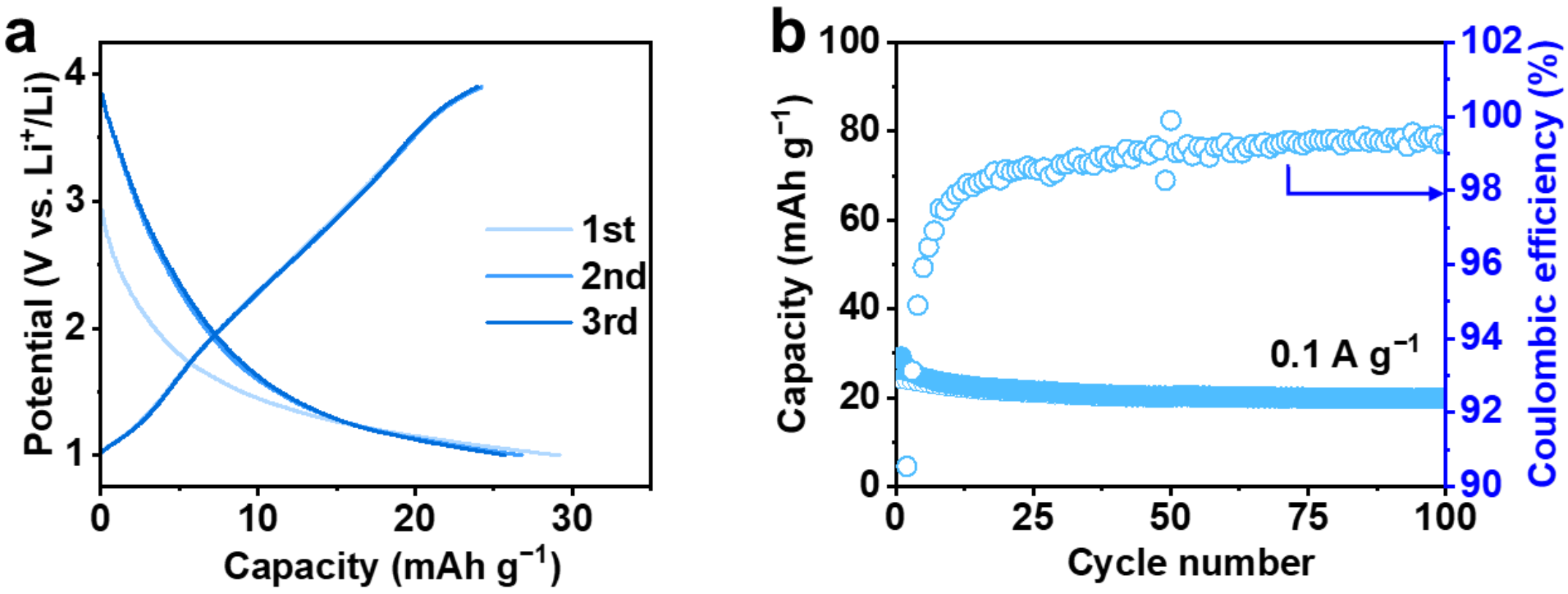

Supplementary Fig. S27 Electrochemical performance of the G/C electrode (composed of 90% G/C mixture and 10% PVDF). **a** GCD profiles and **b** cycling performance of the G/C electrode. The specific capacity is only 24 mAh g^{-1}. According, for an electrode composed of 60% HPBQ, 30% G/C, and 10% PVDF, the specific capacity contribution from G/C in only 7 mAh g^{-1}, which is negligible when compared to the reversible capacity of the HPBQ electrode.

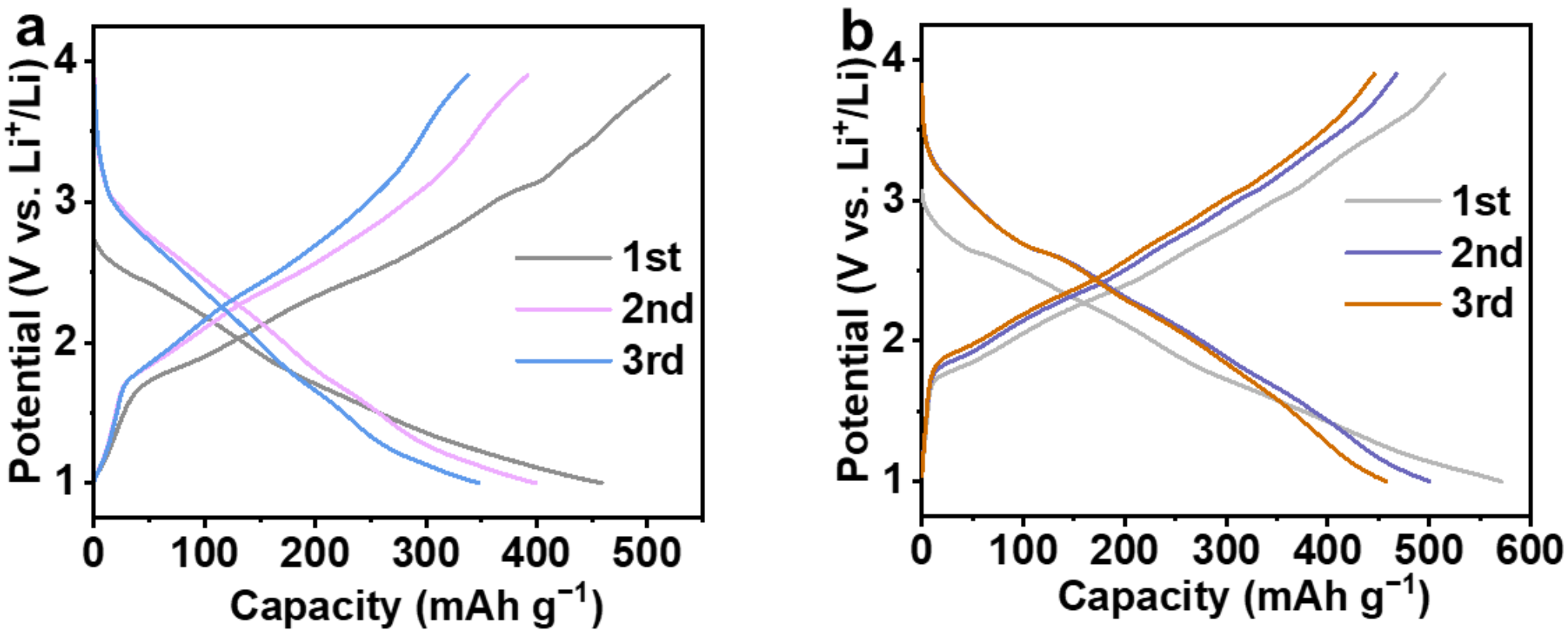

Supplementary Fig. S28 GCD profiles of the **a** QPT and **b** BQ-COF electrodes at 0.05 A g^{-1}.

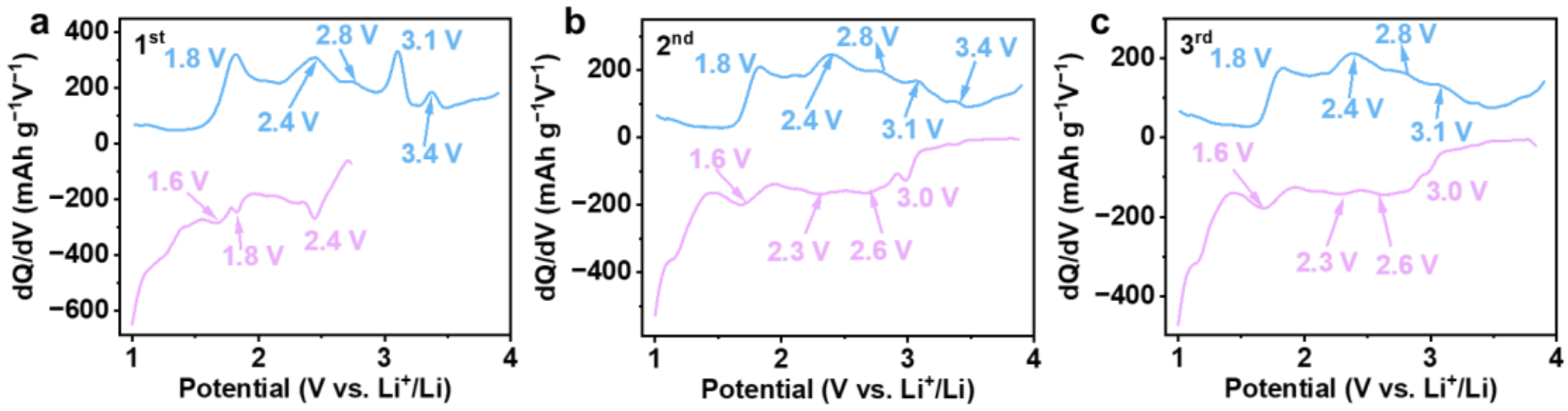

Supplementary Fig. S29 Differential capacity (dQ/dV) profiles of QPT during the **a** 1st , **b** 2nd, and **c** 3rd cycles. The results reveal a distinct extra peak at approximately 3.4 V during the charging process in the initial two cycles. However, this peak disappeared in the third cycle. Furthermore, QPT exhibits four pairs of reversible redox peaks at ~3.0 V/~3.1 V, ~2.6 V/~2.8 V, ~2.3 V/~2.4 V, and ~1.6 V/~1.8 V, indicating a multi-stage Li^+ intercalation process.

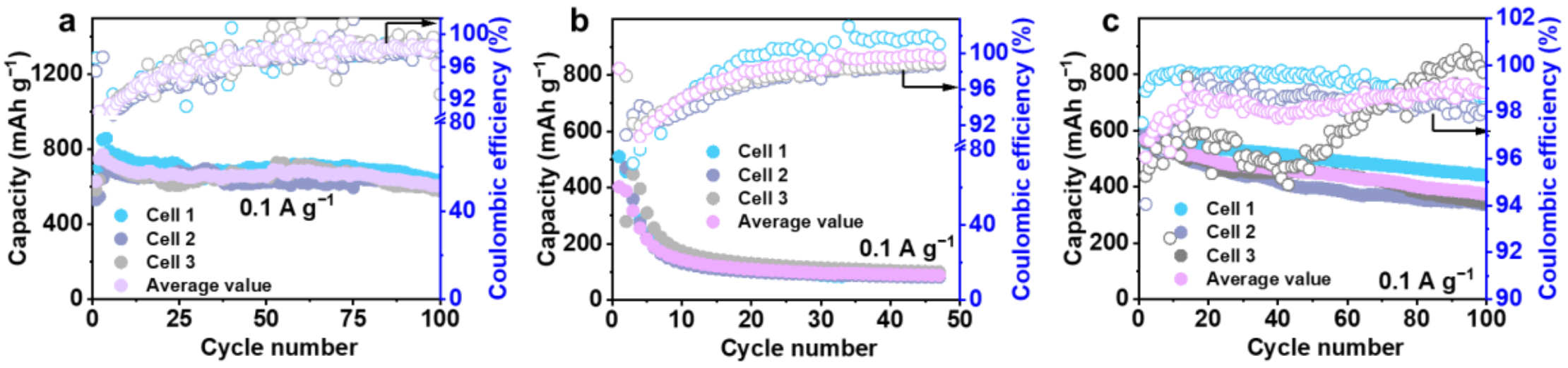

Supplementary Fig. S30 Cycling performance of **a** HPBQ, **b** QPT, and **c** BQ-COF at 0.1 A g^{-1}. Each compound was tested by three different cells.

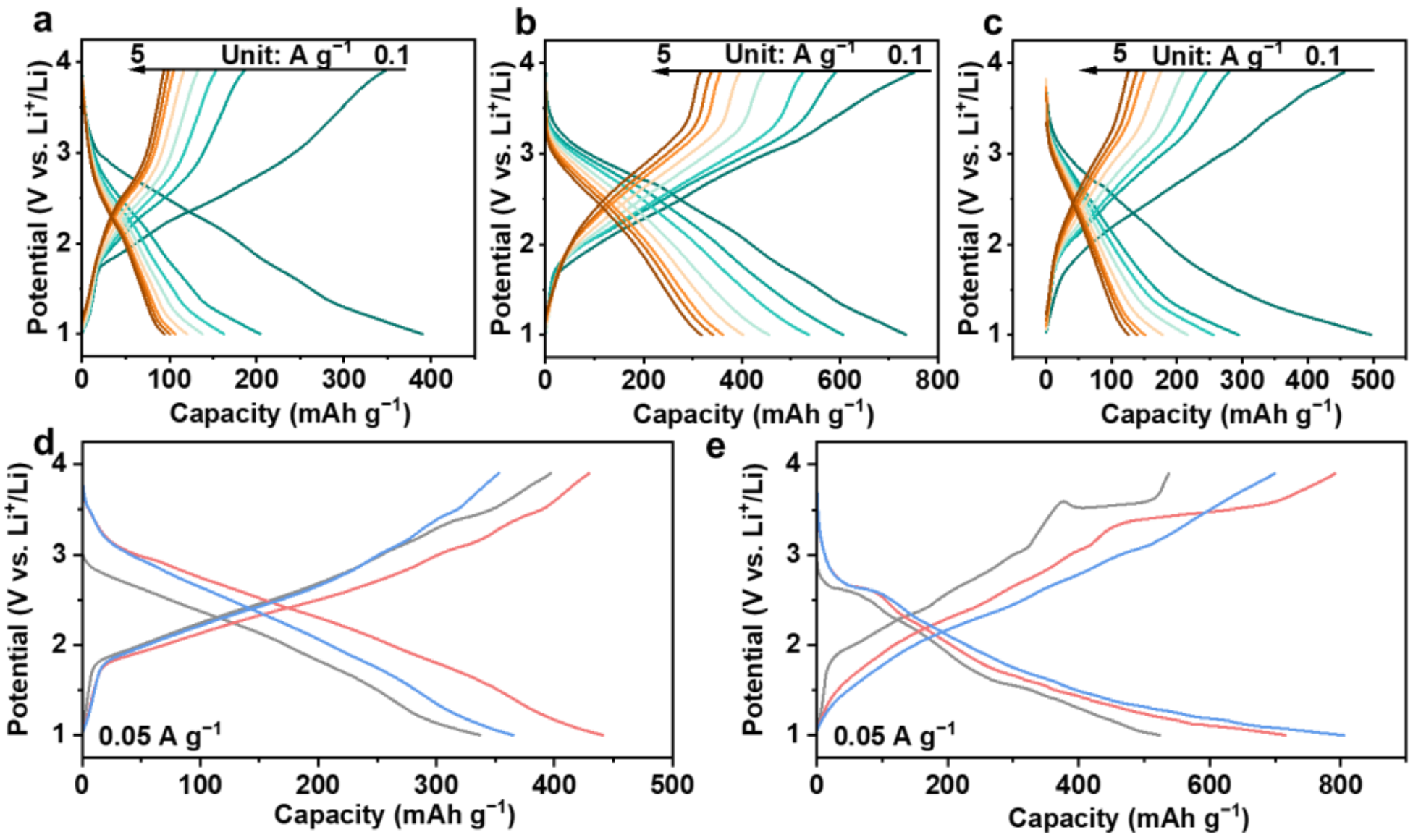

Supplementary Fig. S31 GCD profiles of **a** QPT, **b** HPBQ, and **c** BQ-COF at different current densities. GCD curves of **d** QPT and **e** HPBQ during the three activation cycles at 0.05 A g^{-1}.

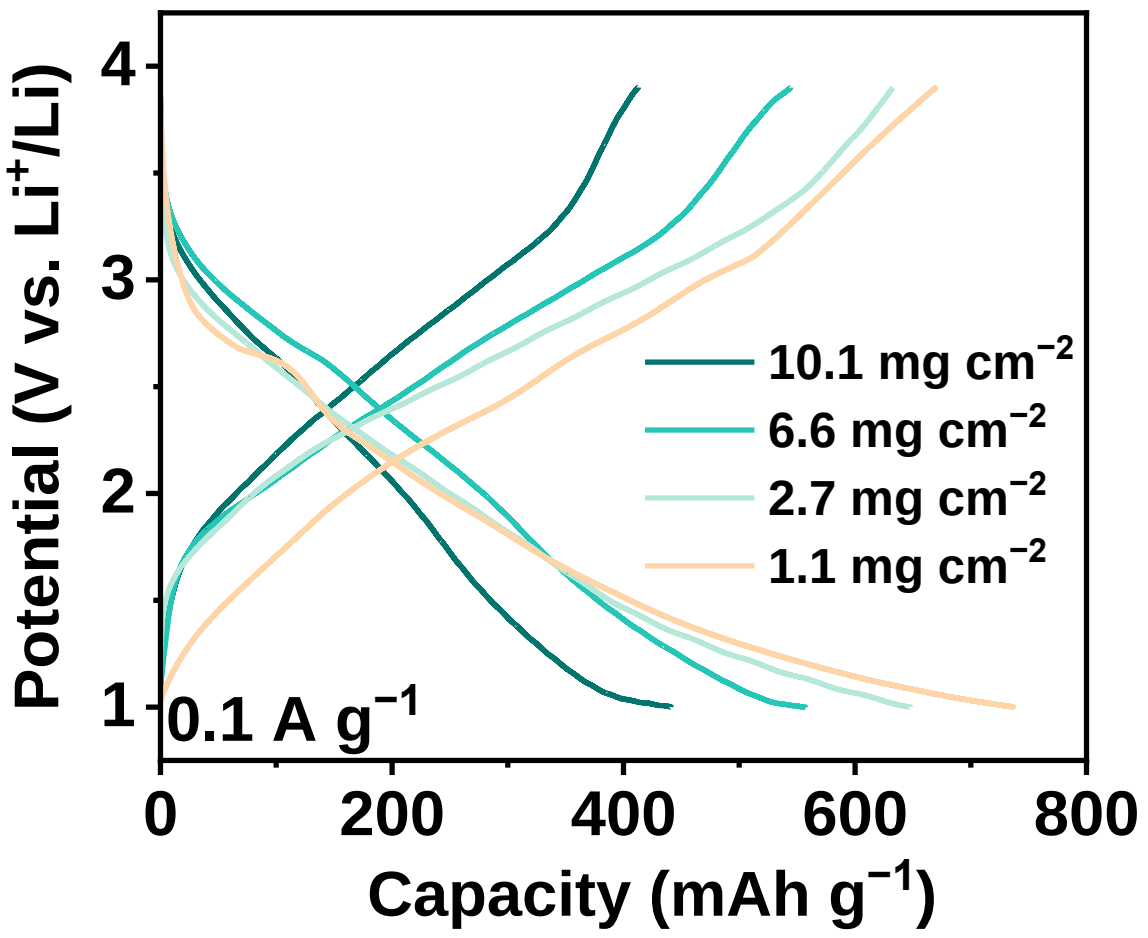

Supplementary Fig. S32 GCD profiles of HPBQ with varying electrode loadings at 0.1 A g^{-1}. For HPBQ electrodes with active material loadings of 1.1, 2.7, 6.6, and 10 mg cm^{-2}, the corresponding specific capacities were 737, 647, 556, and 441 mAh g^{-1}, respectively, showing a clear decreasing trend with increasing loading. This decline is mainly due to the increased electrode thickness, which aggravates the diffusion resistance of Li ions within the solid electrode, leading to larger kinetic polarization.[16] As a result, active material located in the inner regions of the electrode, particularly far from the current collector and electrolyte, cannot be fully activated or utilized.

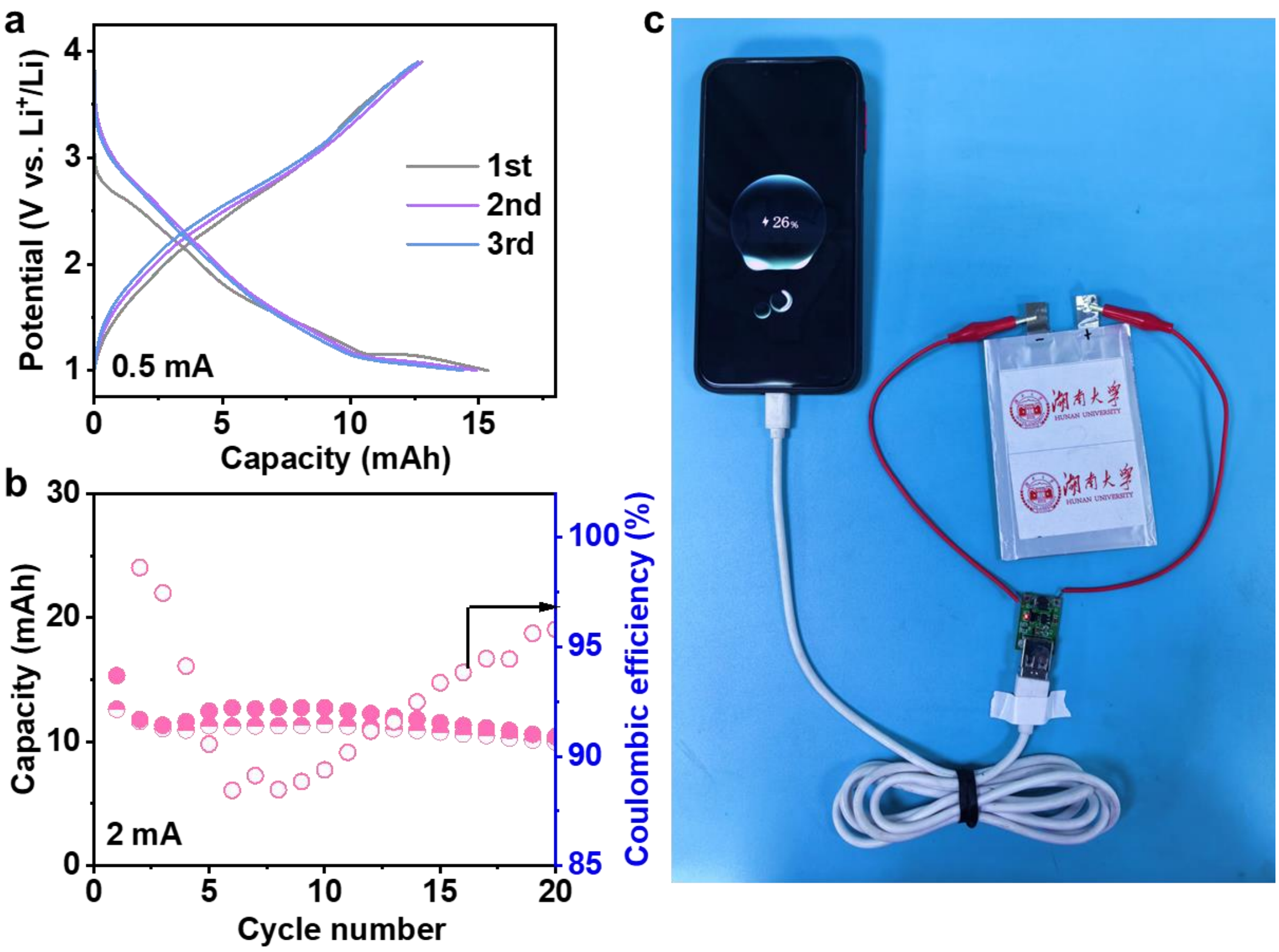

Supplementary Fig. S33 Electrochemical performance of Li||HPBQ pouch cell. **a** GCD curves of a Li||HPBQ pouch cell at a current of 0.5 mA. **b** Cycling stability of the Li||HPBQ pouch cell at a current of 2 mA. **c** Photograph of the pouch cell powering a mobile phone.

To evaluate the feasibility of HPBQ as an electrode material in practical battery devices, a pouch cell was assembled with a HPBQ positive electrode (4.7 × 7.7 cm, mass loading 1 mg cm^{-2}), a lithium-metal negative electrode (same dimensions, 75 μm thick), and a glass-fibre separator (same dimensions). The HPBQ-based pouch cell delivered a reversible capacity of 15 mAh at 0.5 mA and maintained stable cycling over 20 cycles at 2 mA. Meanwhile, the assembled pouch cell can also power mobile phones. These results further demonstrate the feasibility of HPBQ for practical applications.

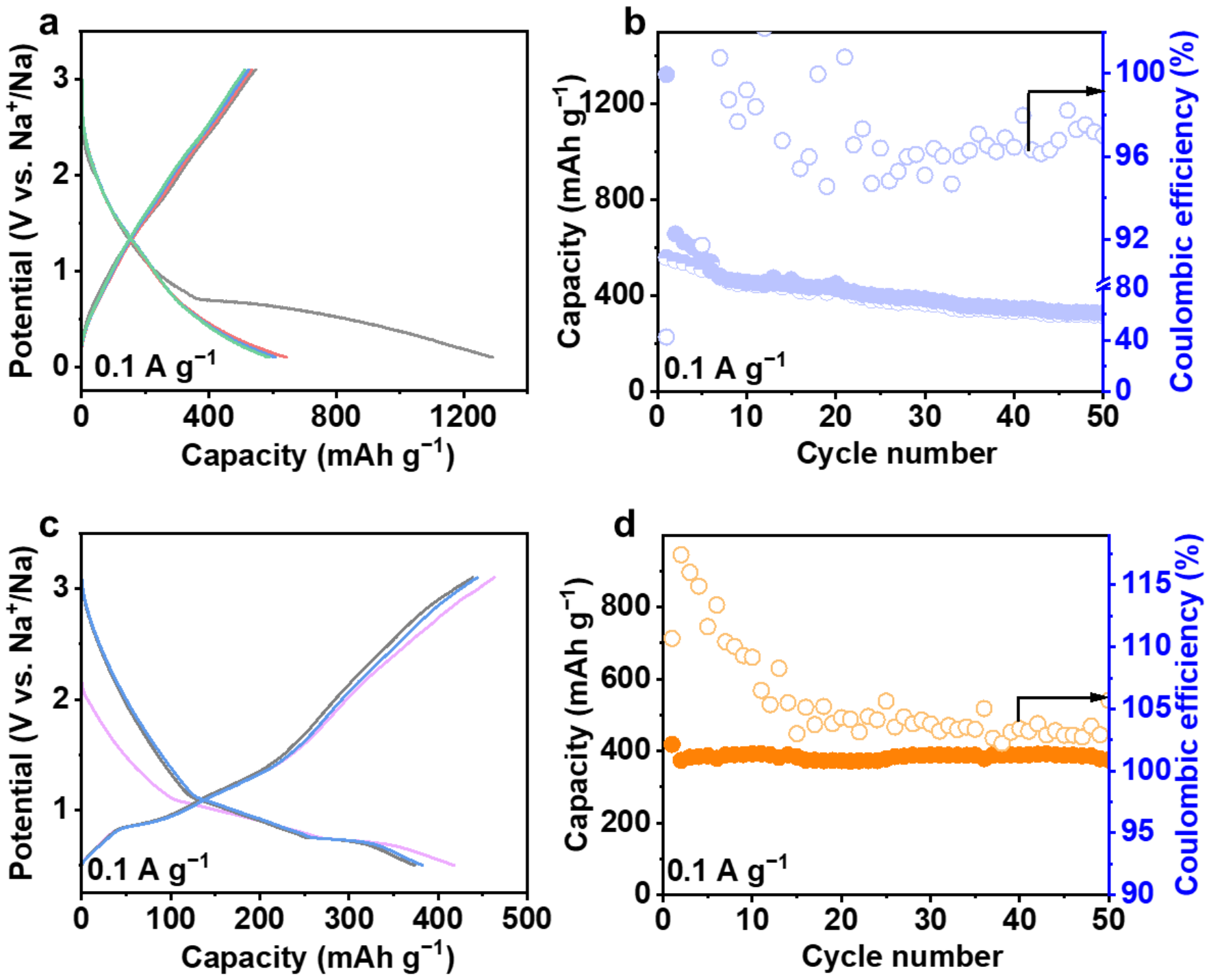

Supplementary Fig. S34 Electrochemical performance of HPBQ in sodium-ion batteries. **a** GCD profiles and **b** cycling stability within a voltage range of 3.1-0.1 V at 0.1 A g^{-1}; **c** GCD profiles and **d** cycling stability within a voltage range of 3.1-0.5 V at 0.1 A g^{-1}

The tests were conducted in a 2 M NaTFSI in DOL/DME electrolyte. Within a voltage range of 3.1–0.1 V (vs. Na^+/Na), HPBQ delivered an initial capacity of 1289 mAh g^{-1} and a low initial Coulombic efficiency of 50%, primarily attributed to the formation of a solid electrolyte interphase (SEI) derived by the decomposition of electrolyte within such a low voltage range.[17] The material achieved a reversible capacity of 639 mAh g^{-1} in this system, but retained only 54% of its capacity after 50 cycles, indicating that this voltage window is unfavorable for long-term cycling stability. Accordingly, the voltage window was optimized to 3.1–0.5 V. Under this condition, HPBQ exhibited an initial discharge capacity of 418 mAh g^{-1} and maintained a high capacity retention of 90% after 50 cycles.

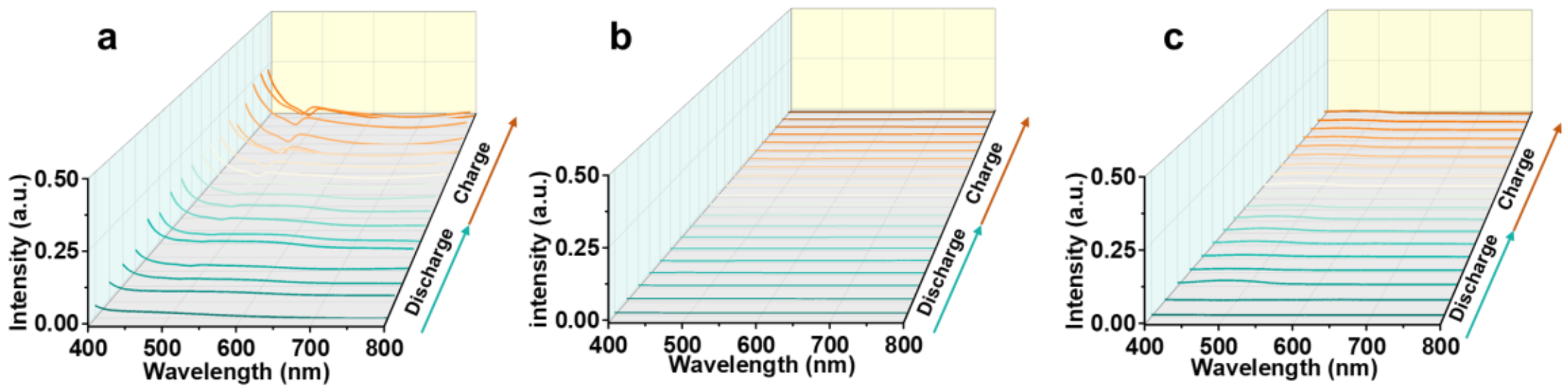

Supplementary Fig. S35 In situ UV–Vis spectroscopy monitoring the discharging/charge processes of **a** QPT, **b** HPBQ, and **c** BQ-COF electrodes. All cells tested at 0.3 A g^{-1} under 25 ±1 ℃.

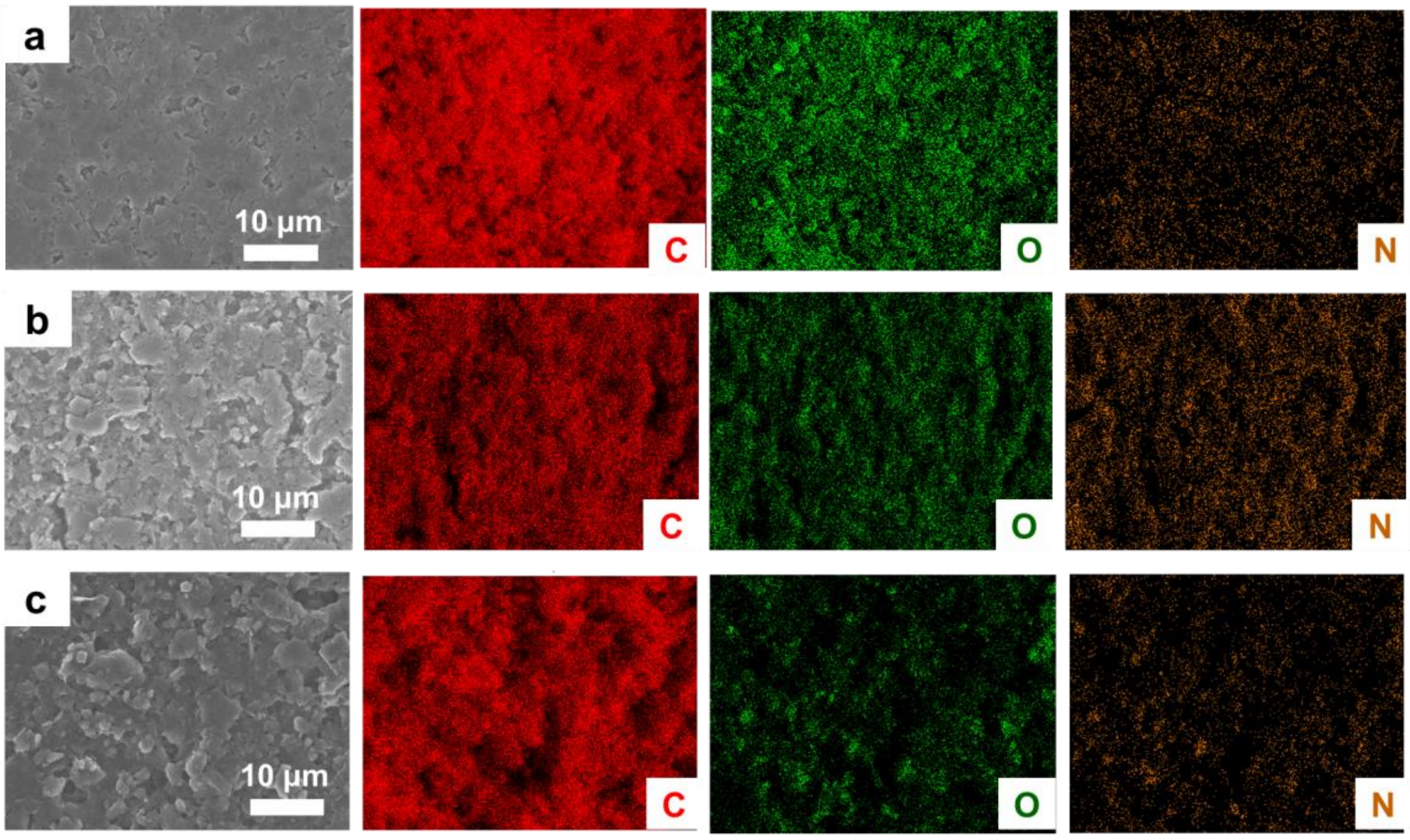

Supplementary Fig. S36 SEM images and corresponding EDS elemental mapping of the pristine **a** QPT electrode, **b** HPBQ electrode, and **c** BQ-COF electrode.

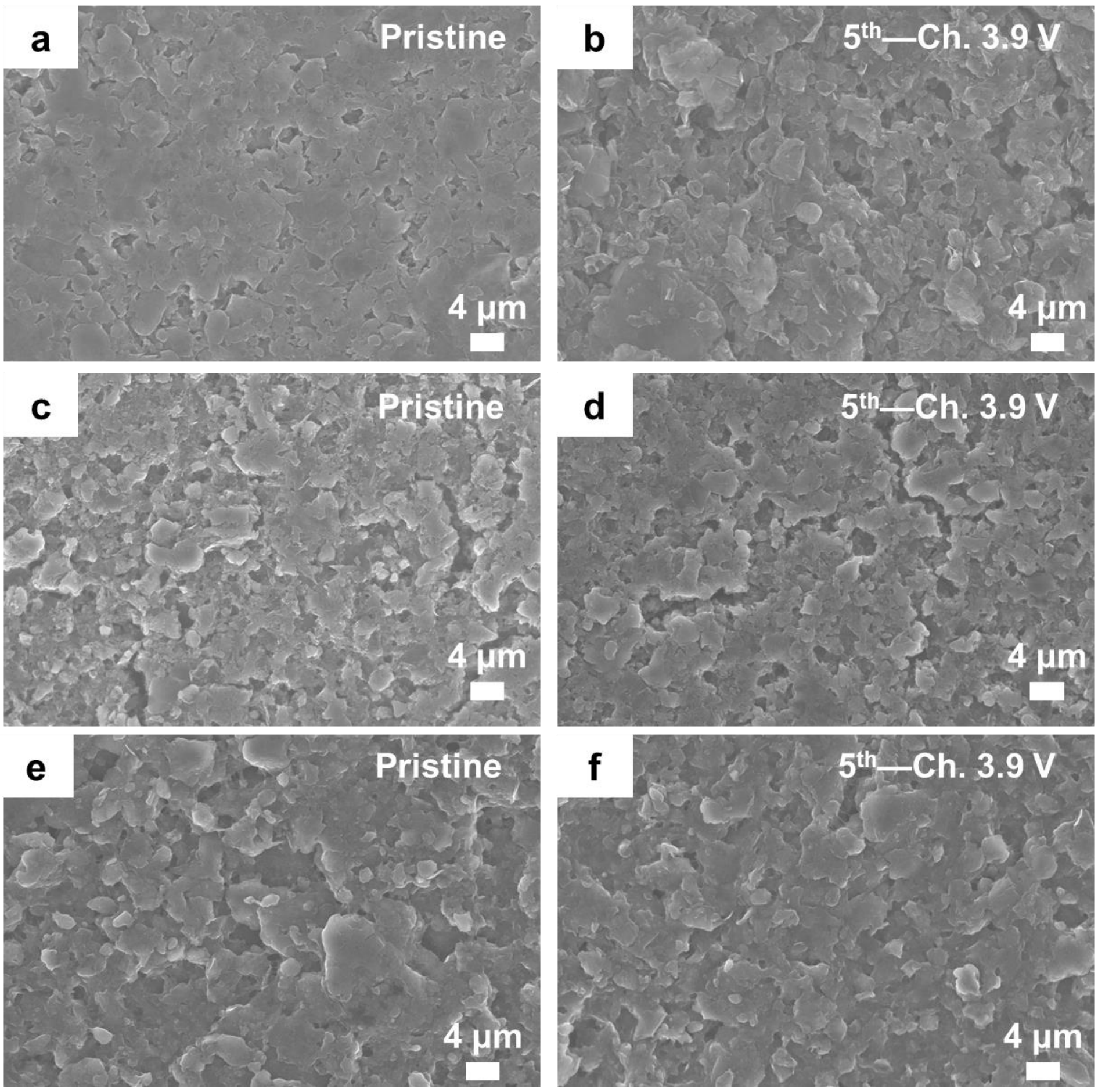

Supplementary Fig. S37 SEM images of **a,b** QPT, **c,d** HPBQ, and **e,f** BQ-COF electrodes in their **a,c,e** initial state and **b,d,f** after 5 cycles.

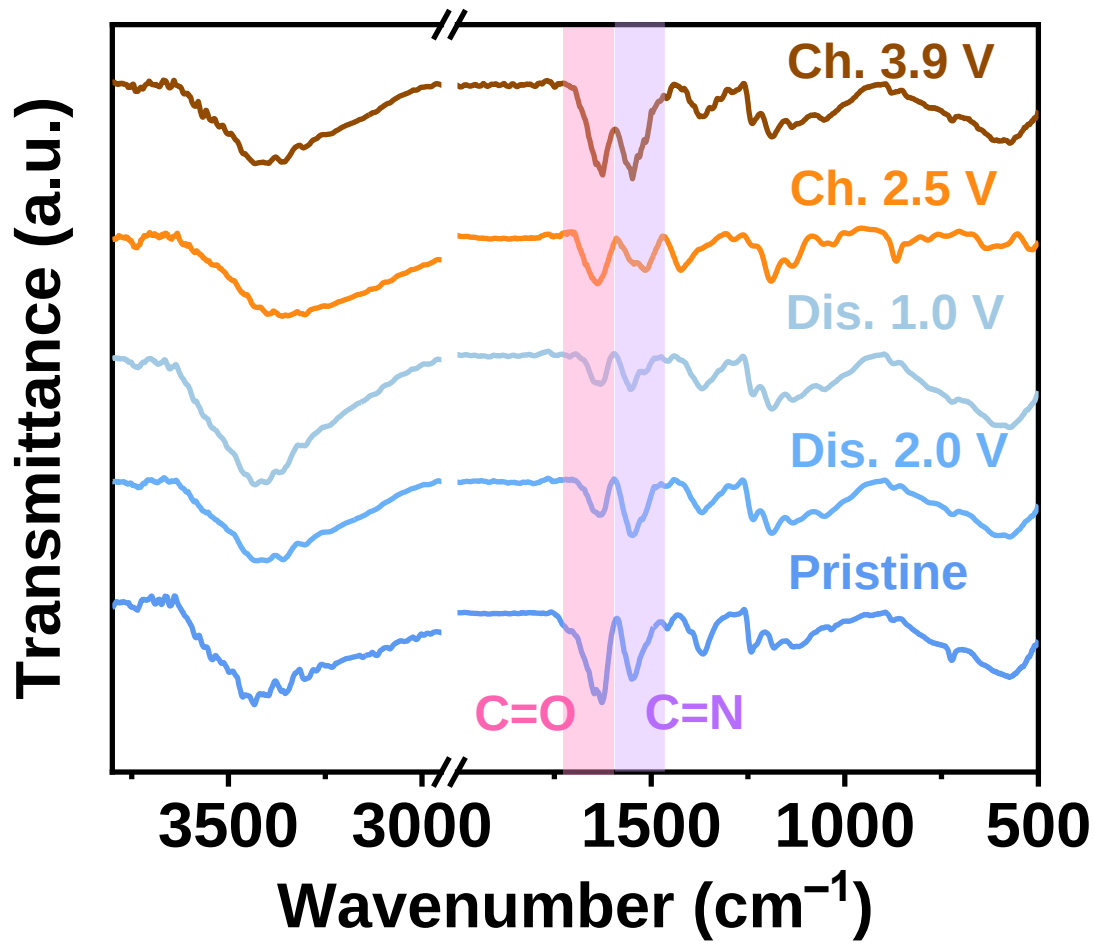

Supplementary Fig. S38 Ex situ FTIR spectra of HPBQ. Each cell was cycled at 0.1 A g^{-1} for 1 cycles at 25 ±1 ℃ under ambient pressure, and terminated at designated states of charge/discharge before disassembly. During the discharge process to 1.0 V, the characteristic peaks at 1680 cm^{-1} (C=O) and 1533 cm^{-1} (C=N) gradually weaken and nearly disappear, and then recover during the subsequent charging process, indicating that C=O and C=N serve as the redox-active centers of the material. In addition, the intensity of the –OH stretching vibration at 3402 cm^{-1} remains almost unchanged during discharge, but decreases in the later stage of charging, suggesting that partial dehydrogenation of hydroxyl groups occurs during this phase.

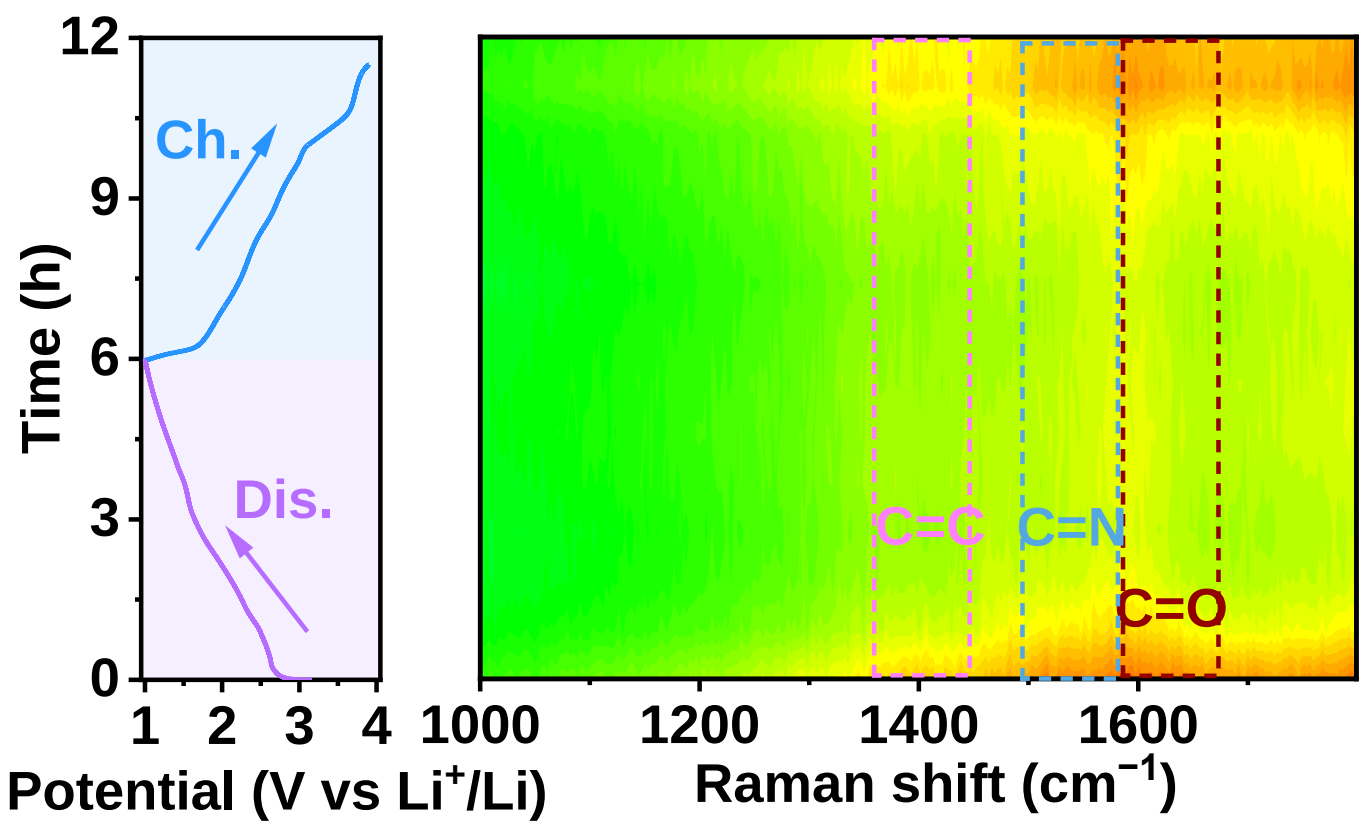

Supplementary Fig. S39 In situ Raman spectra of the HPBQ electrode during the initial cycle. The cell was tested at 0.2 A g^{-1} under 25 ±1 ℃

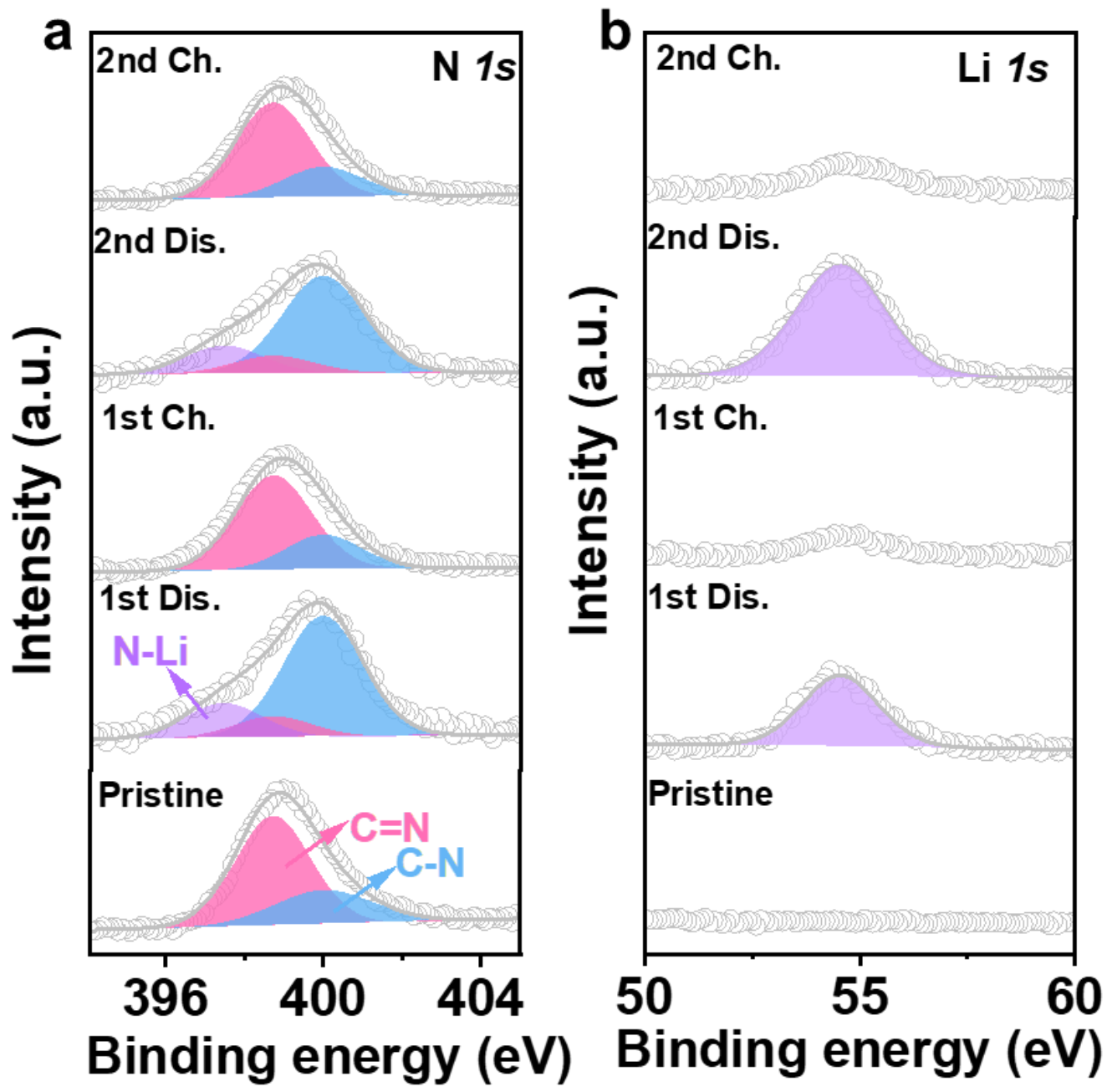

Supplementary Fig. S40 XPS spectra of **a** N *1s* **b** Li *1s* of HPBQ electrodes at different discharging/charging states. All electrodes were taken at fully charged and fully discharged states from cells cycled at 0.1 A g^{-1} and 25 ±1 °C. The cycle number is labeled in the figure.

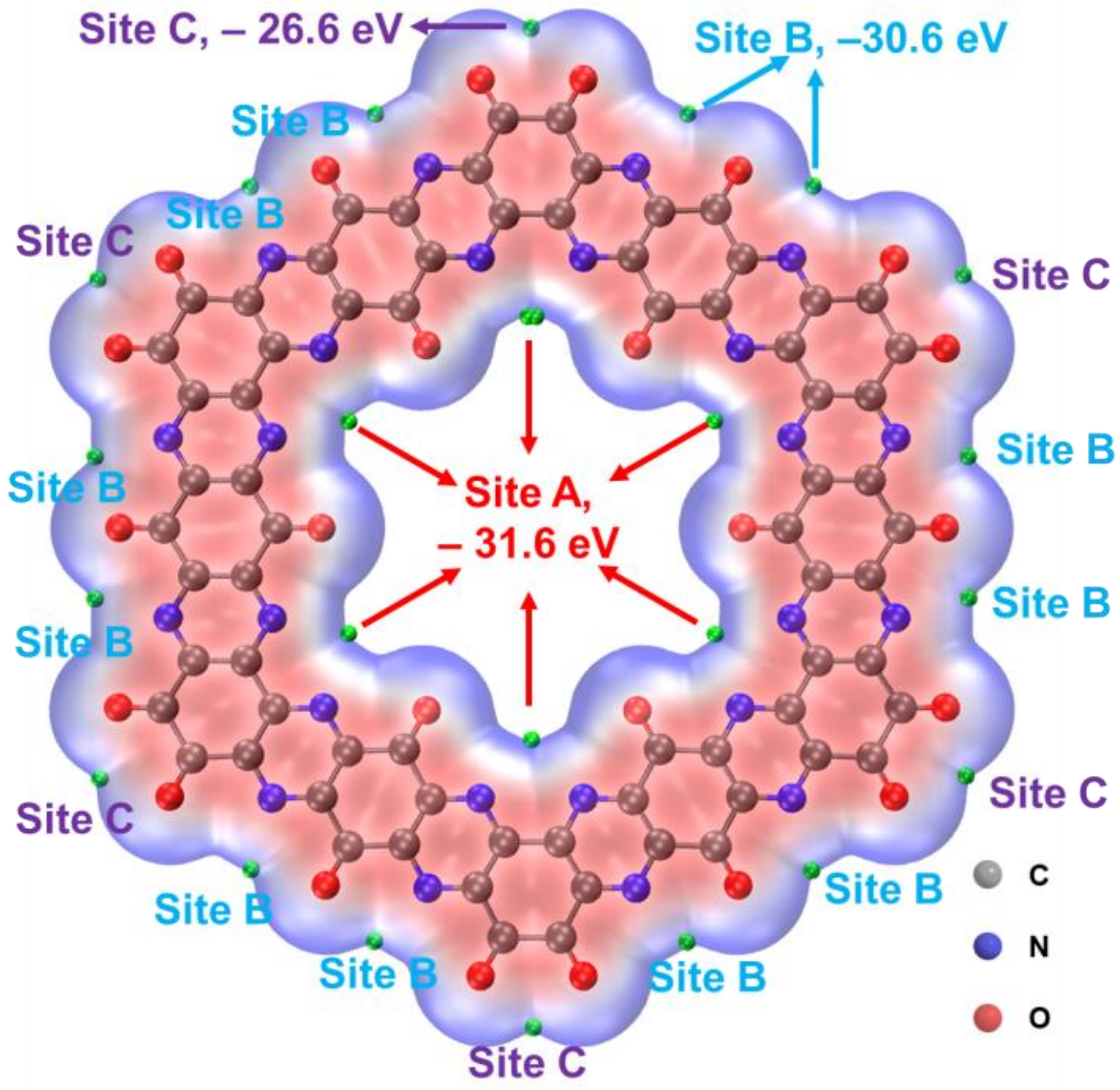

Supplementary Fig. S41 Electrostatic potential (ESP) surface mapping and analysis of the PBQ molecule. The minimum ESP values (most negative) are localized around the oxygen and nitrogen atoms, identifying the C=O and C=N groups as the primary redox-active sites. Analysis of the 48 active sites reveals three distinct categories based on electronegativity: Site A exhibits the most negative value (−31.6 eV), followed by Site B (−30.6 eV), and then Site C (−26.6 eV). This distribution of ESP minima suggests a multi-step, multi-electron redox mechanism during lithiation.

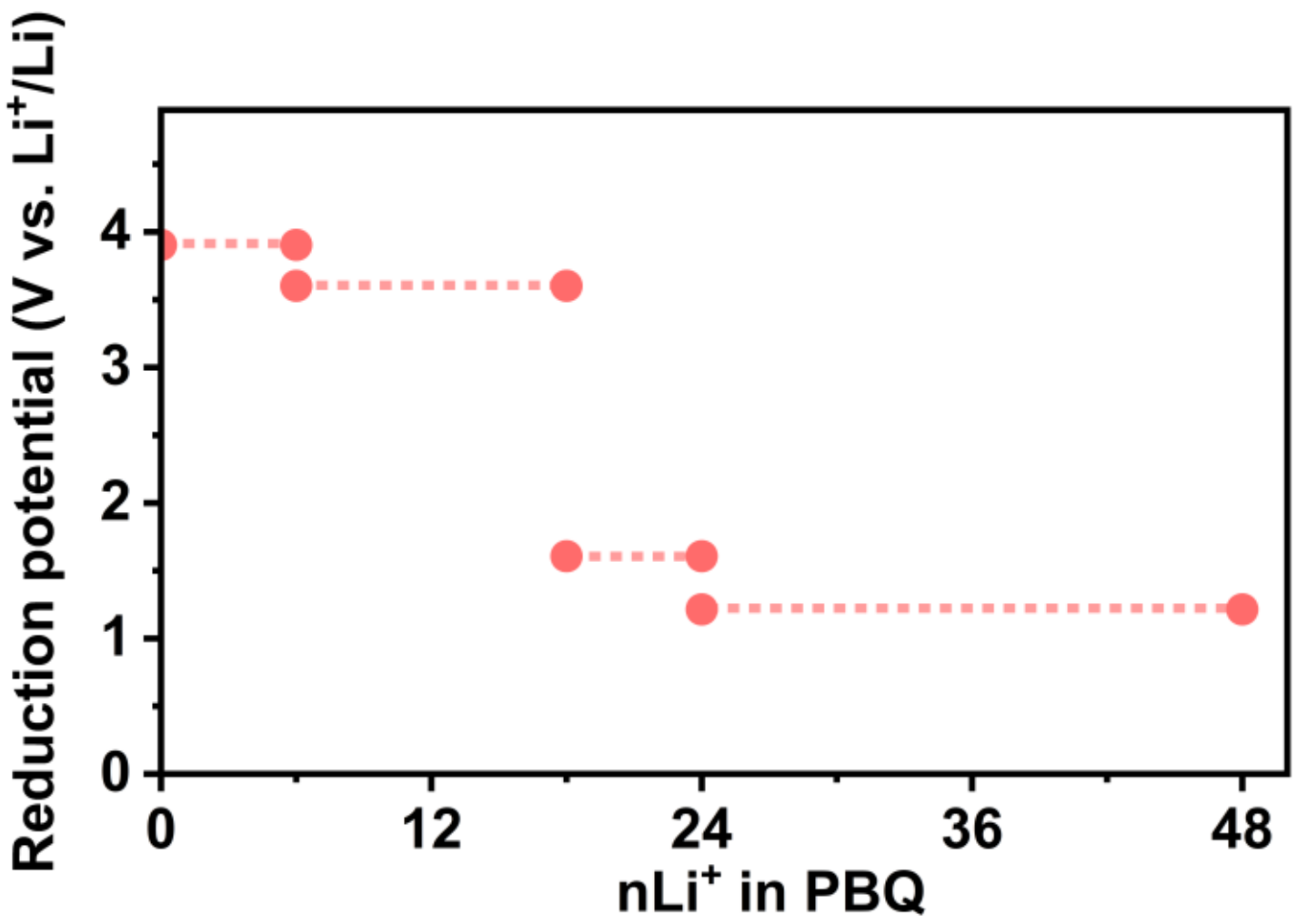

Supplementary Fig. S42 Calculated reduction potential for each lithitation step of PBQ. The material exhibits two distinct redox potential levels (above 3 V and below 2 V). This behavior is attributed to the stronger binding affinity when nitrogen/oxygen atoms coordinate with one lithium atom during the first two lithiation stages, whereas the binding strength decreases significantly when each nitrogen/oxygen atom binds to two lithium atoms in the latter two stages, leading to a sharp decline in the redox voltage in the subsequent stages.[2]

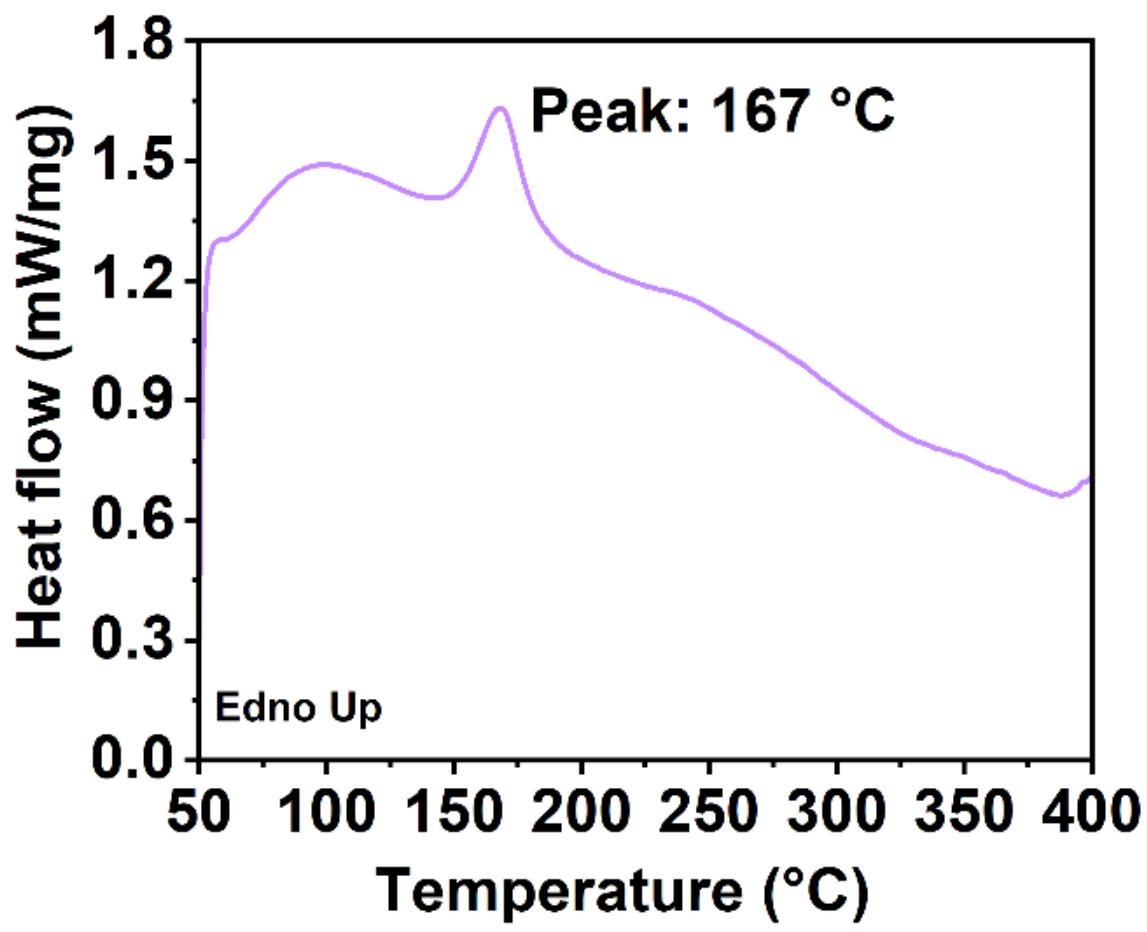

Supplementary Fig. S43 Differential scanning calorimetry (DSC) thermogram of HPBQ recorded under Ar atmosphere at a heating rate of 10 ℃/min.

The DSC thermogram showed a broad endothermic shoulder in the range of 50–100 ℃, which should be attributed to the removal of adsorbed water and bound water in pores. In addition, an pronounced endothermic peak appeared between 150 and 180 ℃, with a peak temperature of approximately 167 ℃, which we assign to the melting of HPBQ. Notably, the full width at half maximum (FWHM) of this melting endotherm is approximately 15 ℃, further suggesting the presence of impurities.

Supplementary Table S1. Elemental analysis of HPBQ. The experimental results are obtained based on two different types of equipment (C, H, and N recorded by Vario Micro Cube Elemental Analyzer; O detected by Vario EL cube Elemental Analyzer)

	Mass fraction of C (%)	Mass fraction of N (%)	Mass fraction of O (%)	Mass fraction of H (%)
Calculated for HPBQ	54.15	21.05	24.04	0.76
EA-1[a] results	53.97	21.12	24.11	0.80
EA-2 results	47.68	23.73	26.83	1.78
EA-3 results	49.89	22.22	26.23	1.66

[a] EA-1, EA-2, and EA-3 represent elemental analyses of three different independently synthesized batches.

Supplementary Table S2. Theoretical calculated lattice parameters of crystalline phase of HPBQ.

a (nm)	b (nm)	c (nm)	α (°)	β (°)	γ (°)	V (nm^3)
0.833	0.679	0.991	86.28	47.90	72.34	0.4023

Supplementary Table S3. Elemental analysis of QPT. The experimental results are obtained based on two different types of equipment (C, H, and N recorded by Vario Micro Cube Elemental Analyzer; O detected by Vario EL cube Elemental Analyzer). The experimentally determined contents of C, N, O, and H were in close agreement with the theoretically calculated proportions, indicating the successful synthesis of the target product PQT.

	Mass fraction of C (%)	Mass fraction of N (%)	Mass fraction of O (%)	Mass fraction of H (%)
Calculated for QPT	65.83	11.81	20.24	2.12
EA results	66.27	10.85	20.85	2.06

Supplementary Table S4. Elemental analysis of BQ-COF. The experimental results are obtained based on two different types of equipment (C, H, and N recorded by Vario Micro Cube Elemental Analyzer; O detected by Vario EL cube Elemental Analyzer). The contents of C, N, and O were 53.69%, 21.19%, and 22.85%, respectively. The excess oxygen content can be attributed to edge groups or small molecules (such as H_2O, CO_2, and CH_3OH) adsorbed within the pores.[4]

	Mass fraction of C (%)	Mass fraction of N (%)	Mass fraction of O (%)	Mass fraction of H (%)
Calculated for BQ-COF	62.5	25	12	0
EA results	53.69	21.19	22.85	2.27

Supplementary Table S5. Comparison of capacity, energy density, and cycling performance of this work with reported organic cathodes for LIBs. All tests at 25 ± 1 °C.

Name	Potential Range (V)	Capacity (mAh g^{-1}), Utilization rate (%)	Specific energy [a] (Wh kg^{-1})	Retention rate (%), Cycle number, Current density (A g^{-1})	Ref.
HATN-AQ-COF	1.2-3.9	319, 89%	638	80%, 3000, 3.58	18
BQ1-COF	1.2-3.5	494, 64%	1111.5	81%, 1000, 1.55	19
PIGN	1.5-4.0	244, 84%	549	None	20
PIGN-G	1.5-4.0	271, 97%	610	86%, 300, 2.8	20
HTPT-COF	1.2-3.5	402, 42%	869	51%, 1000, 5	21
HTPT-COF@CNT22	1.2-3.5	507, 53%	1090	77.4%, 1000, 5	21
PGF-1	1-3.6	842,129%	1431	78.3%, 1400, 0.5	22
USTB	1.2-3.9	158, 58.3%	340	30%, 500,0.5	23
USTA-6@GO	1.2-3.9	285, 105%	613	70%, 6000, 2.5	23
PT-COF@CNT50	1.5-3.5	280, 103%	700	82%, 3000, 2	24
PT-COF	1.5-3.5	193, 71%	434	99%, 150,0.2	24
CCP	1.5-3.5	231, 30%	578	None	25
TADBPHO	1.5-3.8	364, 65%	932	71%, 500, 0.5	26
TAPT	1.3-3.5	361, 67%	884	80%, 1200, 2	27
HATAQ	1.2-3.8	426, 85%	937.2	85%, 1000, 10	28
3Q	1.2-3.9	395, 98%	711	70%, 10000, 8	2
PTO-NH_2	1.5-3.8	350, 95%	980	83%, 100, 0.05	29
3BQ	1.3-1.9	506, 98%	1113.2	70%, 300, 0.515	30
TAQ	1.6-3.2	306, 86%	765	70%, 2000, 1	31
C_6O_6[b]	1.2-4.0	902, 94%	1533	82%, 100, 0.05	32
C4Q	1.5-3.5	431, 97%	1077	90, 100, 0.1	33
DPmT	1.2-4.2	653, 97%	1188	80%, 500, 0.5	34
DQDPD	1.0-3.5	505, 97%	1111	82%, 3000, 5	35
3PMDI-△	1.8-3.2	172, 62%	408	64, 40, 0.1	36
**HPBQ**	**1.0-3.9**	**806, 99%**	**1507**	**81%, 1000, 5**	**This work**

[a] Energy density was calculated based on the mass of the organic active materials.

Supplementary Table S6. Summary of ICP-OES data for the HPBQ electrode at the 1st and 3rd discharged states.

Electrode for different discharged states	Initial electrode mass (m_1, g)	Post-discharge electrode mass (m_2, g)	ICP-OES measured Li element content (w, %)	Average Li element content (w_a, %)
1st	0.0343	0.0349	1.5309<br>1.5236<br>1.5551	1.5365
3rd	0.0317	0.0324	1.1925<br>1.2263<br>1.2076	1.2088

To quantitatively evaluate the multielectron redox processes, we analyzed the lithium intercalation levels at key discharge states, specifically, the 1st discharged state corresponding to 36-electron transfer and the 3rd discharged state corresponding to 48-electron transfer.

ICP-OES was performed on electrodes at the 1st and 3rd discharged states (Supplementary Table S6). Specifically, the initial masses (m_1) of the two electrodes before cycling were recorded. The electrodes were then assembled into coin-type cells and subjected to galvanostatic charge–discharge at a current density of 0.05 A g^{-1}. After cycling, the cells were disassembled in an argon-filled glovebox. The disassembled HPBQ electrode was rinsed with DME and then immersed in a centrifuge tube containing DME for 6 hours. Subsequently, the electrode was rinsed again with DME, followed by replacement of the DME in the centrifuge tube and another 6-hour immersion. This cleaning procedure was repeated five times to thoroughly remove the residual salt from the electrode. The electrodes were then vacuum-dried at 60°C for 24 h to fully eliminate the solvent, after which the mass of each discharged electrode (m_2) was measured. The entire electrode (including the current collector) was subjected to microwave digestion for ICP-OES analysis, and the average lithium contents were determined as the average of triplicate measurements. All measured data are summarized in Supplementary Table S6.

To calculate the actual amount of lithium ions inserted during the 1st discharge process, the

molar quantity of HPBQ in the pristine electrode, n_{HPBQ}, was first determined according the following equation:

$$n_{HPBQ} = \frac{m_{HPBQ}}{M_{HPBQ}} = \frac{(m_1 - m_0) \times 60\%}{M_{HPBQ}}$$

where m_1 represents the total mass of the electrode before cycling (0.0343 g), m_0 denotes the mass of the titanium mesh current collector (0.0282 g), 60% represents the active material content in the HPBQ electrode, and M_{HPBQ} represents the molar mass of HPBQ (1596 g/mol). Accordingly, n_{HPBQ} in this electrode was calculated to be 2.29×10^{-6} mol.

The actual molar quantity of lithium-ion inserted in this the HPBQ electrode during the 1st discharge process (n'_{1st-Li}) can be calculated as:

$$n'_{1st-Li} = \frac{m_2 \times w_a}{M_{Li}}$$

where m_2 is the total mass of the electrode after the 1st discharge process (0.0343 g), w_a is the measured average Li element content in the discharged HPBQ electrode (1.5365%), and M_{Li} is the molar mass of Li (6.94 g/mol). Accordingly, n'_{1st-Li} was calculated to be 7.59×10^{-5} mol.

The actual number of lithium ions inserted into HPBQ during the 1st discharge, Q_{1st-Li}, was then determined by the ratio of the molar amount of inserted lithium ions (n'_{1st-Li}) to the molar amount of HPBQ (n_{HPBQ}):

$$Q_{1st-Li} = \frac{n'_{1st-Li}}{n_{HPBQ}} = \frac{7.59 \times 10^{-5}}{2.29 \times 10^{-6}} = 33.14$$

Following the same calculation process for the 3rd discharge cycle, the actual number of lithium ions inserted into HPBQ was determined as follows.

The molar quantity (n_{HPBQ}) of HPBQ in the pristine electrode:

$$n_{HPBQ} = \frac{m_{HPBQ}}{M_{HPBQ}} = \frac{(m_1 - m_0) \times 60\%}{M_{HPBQ}} = \frac{(0.0317\ \ g - 0.0282\ \ g) \times 0.6}{1596 g/mol}$$
$$= 1.32 \times 10^{-6}\ \ mol$$

Actual molar quantity of lithium-ion inserted in the HPBQ electrode during the 3rd discharge (n'_{3rd-Li}):

$$n'_{3rd-Li} = \frac{m_2 \times w}{M_{Li}} = \frac{0.0324\ \ g \times 1.2088\%}{6.94\ \ g/mol} = 5.64 \times 10^{-5} mol$$

The actual number of lithium ions intercalated during the 3rd discharge (Q_{3rd-Li}):

$$Q_{3rd-Li} = \frac{n'_{3rd-Li}}{n_{PBQ}} = \frac{5.64\times10^{-5}}{1.32\times10^{-6}}=42.73$$

Based on these data, the experimentally determined numbers of intercalated lithium ions in HPBQ at the 1st and 3rd discharged states were calculated to be 33.14 and 42.73, respectively, which align closely with the theoretical values of 36 and 48. This close correspondence between experimental data and theoretical predictions quantitatively confirms the proposed multielectron reaction mechanism of HPBQ and validates the reliability of the electrochemical analysis presented in the manuscript.

References:

1. Lin Z, *et al*. A high capacity small molecule quinone cathode for rechargeable aqueous zinc-organic batteries. *Nat. Commun.* **12**, 4424 (2021).
2. Peng C*, et al.* Reversible multi-electron redox chemistry of π-conjugated n-containing heteroaromatic molecule-based organic cathodes. *Nat. Energy* **2**, 17074 (2017).
3. Ding C, *et al*. Regulating intermolecular hydrogen bonds in organic cathode materials to realize ultra-stable, flexible and low-temperature aqueous zinc-organic batteries. *Angew. Chem. Int. Ed.* **64**, e202417988 (2024).
4. Shi R*, et al.* Nitrogen-rich covalent organic frameworks with multiple carbonyls for high-performance sodium batteries. *Nat. Commun.* **11**, 178 (2020).
5. Tian Z*, et al.* High-capacity NH_4^+ charge storage in covalent organic frameworks. *J. Am. Chem. Soc.* **143**, 19178-19186 (2021).
6. Guan X, *et al*. Functional regulation and stability engineering of three-dimensional covalent organic frameworks. *Acc. Chem. Res.* **55**, 1912-1927 (2022).
7. Cabañero M*, et al.* Are polymer-based electrolytes ready for high-voltage lithium battery applications? An overview of degradation mechanisms and battery performance. *Adv. Energy Mater.* **12**, 2201264 (2022).
8. He X*, et al.* The passivity of lithium electrodes in liquid electrolytes for secondary batteries. *Nat. Rev. Mater.* **6**, 1036-1052 (2021).
9. Marrache R, *et al*. Elucidation of losses in cycling lithium-metal anodes in ether-based electrolytes. *J. Electrochem. Soc.* **170**, 040512 (2023).
10. Wang Q*, et al.* Designing organic material electrodes for lithium-ion batteries: Progress,

challenges, and perspectives. *Electrochem. Energy Rev.* **7**, 15 (2024).

11. Wang M*, et al.* Weakening solvent polarity enables shuttle-effect-free and temperature-independent lithium-organic batteries. *Energy Storage Mater.* **60**, 102816 (2023).
12. Feng Q*, et al.* Upgrading traditional liquid electrolyte via in situ gelation for future lithium metal batteries. *Sci. Adv.* **4**, eaat5383 (2018).
13. Chen D*, et al.* Origin of extra capacity in the solid electrolyte interphase near high-capacity iron carbide anodes for li ion batteries. *Energy Environ. Sci.* **13**, 2924-2937 (2020).
14. Shi Y, *et al*. Quinone-amine polymer nanoparticles prepared through facile precipitation polymerization as ultrafast and ultralong cycle life cathode materials for lithium-ion batteries. *Adv. Funct. Mater.* **32**, 2111307 (2022).
15. Yu Q*, et al.* Electrochemically manipulating the redox state of 2,2′,5,5′-tetrahydroxybiphenyl as a new organic li-rich cathode for li-ion batteries. *Org. Electron.* **81**, 105661 (2020).
16. Zhu M*, et al.* Spatially coupling electronic–ionic transport in organic mixed conductors as cathodes for efficient Zn–V batteries. *Angew. Chem. Int. Ed.* **64**, (2025).
17. Wang Y*, et al.* A donor–acceptor-donor-type organic molecule as an effective electrode to form rapid diffusion pathway for high-performance sodium-ion batteries. *Angew. Chem. Int. Ed.* **64**, (2025).
18. Yang X*, et al.* Mesoporous polyimide-linked covalent organic framework with multiple redox-active sites for high-performance cathodic Li storage. *Angew. Chem. Int. Ed.* **61**, e202207043 (2022).

19. Wu M, *et al.* A 2d covalent organic framework as a high-performance cathode material for lithium-ion batteries. *Nano Energy* **70**, 104498 (2020).

20. Luo Z, *et al.* A microporous covalent-organic framework with abundant accessible carbonyl groups for lithium-ion batteries. *Angew. Chem. Int. Ed.* **57**, 9443-9446 (2018).

21. Wang Y, *et al*. Crafting core–shell heterostructures with enriched active centers for high-energy-density symmetric lithium-ion batteries. *ACS Nano* **18**, 23958–23967 (2024).

22. Li X, *et al.* Dynamic covalent aynthesis of crystalline porous graphitic frameworks. *Chem* **6**, 933-944 (2020).

23. Liu X, *et al.* In situ growth of covalent organic framework nanosheets on graphene as the cathode for long-life high-capacity lithium-ion batteries. *Adv. Mater.* **34**, 2203605 (2022).

24. Gao H, *et al.* A pyrene-4,5,9,10-tetraone-based covalent organic framework delivers high specific capacity as a li-ion positive electrode. *J. Am. Chem. Soc.* **144**, 9434-9442 (2022).

25. Zheng Y, *et al.* Accelerating ion dynamics under cryogenic conditions by the amorphization of crystalline cathodes. *Adv. Mater.* **33**, 2102634 (2021).

26. Chen Z, *et al.* Constructing extended π-conjugated molecules with o-quinone groups as high-energy organic cathode materials. *ACS Appl. Mater. Interfaces* **14**, 27994-28003 (2022).

27. Li Z, *et al.* A small molecular symmetric all-organic lithium-ion battery. *Angew. Chem. Int. Ed.* **61**, e202207221 (2022).

28. Wu M, *et al.* Supramolecular self-assembled multi-electron-acceptor organic molecule as high-performance cathode material for Li-ion batteries. *Adv. Energy Mater.* **11**, 2100330

(2021).

29. Zheng S, *et al.* Hydrogen bond networks stabilized high-capacity organic cathode for lithium-ion batteries. *Angew. Chem. Int. Ed.* **62**, e202217710 (2023).

30. Zhang Y, *et al*. An all-organic symmetric battery based on a triquinoxalinylene derivative with different redox voltage active sites and a large conjugation system. *J. Mater. Chem. A* **9**, 26208-26215 (2021).

31. Chen T, *et al*. A layered organic cathode for high-energy, fast-charging, and long-lasting li-ion batteries. *ACS Cent. Sci.* **10** 569–578 (2024).

32. Lu Y, *et al.* Cyclohexanehexone with ultrahigh capacity as cathode materials for lithium-ion batteries. *Angew. Chem. Int. Ed.* **58**, 7020-7024 (2019).

33. Huang W, *et al.* Quasi-solid-state rechargeable lithium-ion batteries with a calix[4]quinone cathode and gel polymer electrolyte. *Angewandte Chemie International Edition* **52**, 9162-9166 (2013).

34. Xiao S, *et al.* S-tetrazine-bridged 2,2′-bipyrimidine as superior atom-economic multi-charge cathode material for lithium-ion batteries. *Adv. Energy Mater.* **14**, 2402247 (2024).

35. Peng H, *et al.* Boosting lithium storage performance of small-molecule organic cathodes through synergistic molecular engineering and nanostructure design. *Angew. Chem. Int. Ed.* **64**, e202502088 (2025).

36. Kim DJ, *et al.* Redox-active macrocycles for organic rechargeable batteries. *J. Am. Chem. Soc.* **139**, 6635-6643 (2017).
