# Supplementary material for: Macrocyclic molecular engineering enables high-energy and durable organic electrode for lithium-ion batteries
Source: Nat Commun. 2026 Aug 29;17:10312. doi: 10.1038/s41467-026-76984-5 (PMC13620139; doi:10.1038/s41467-026-76984-5)
Supplement: Supplementary file 2 [file 41467_2026_76984_MOESM2_ESM.pdf]

Description of Additional Supplementary Files

Supplementary Data 1: Simulated X-ray diffraction (XRD) patterns for three different stacking modes (AA, AB, and ABC) of BQ-COF. These data confirm the energetically most favorable stacking arrangement and provide quantitative structural evidence for COF crystallinity.

Supplementary Data 2: Electrostatic potential (ESP) surfaces of HPBQ and PBQ molecules, calculated using density functional theory (DFT) at the B3LYP-D3(BJ)/ 6-31G(d) level. The ESP maps are visualized as color-coded isosurfaces, illustrating the charge distribution and nucleophilic/electrophilic regions of the two quinone derivatives.

Supplementary Data 3: The calculated reaction pathways for the lithiation process of PBQ, as well as the optimized geometric structures and the corresponding free energies, were computed. The calculations were employed to evaluate the thermodynamic and kinetic feasibility of consecutive lithium intercalation. These results provide support for the discussion on the electrochemical mechanism and capacity of the PBQ electrode.
