# Supplementary material for: Macrocyclic molecular engineering enables high-energy and durable organic electrode for lithium-ion batteries
Source: Nat Commun. 2026 Aug 29;17:10312. doi: 10.1038/s41467-026-76984-5 (PMC13620139; doi:10.1038/s41467-026-76984-5)
Supplement: Supplementary file 3 [file 41467_2026_76984_MOESM4_ESM.pdf]

nature portfolio

Peer Review File

Macrocyclic Molecular Engineering Enables High-Energy and Durable Organic Electrode for Lithium-Ion Batteries

Corresponding Author: Professor Zhiqiang Zhu

This file contains all reviewer reports in order by version, followed by all author rebuttals in order by version.

Version 0:

Reviewer comments:

Reviewer #1

(Remarks to the Author)
The authors report on HPBQ as an electrode material. It was synthesized by condensation between rhodizonic acid and tetraaminobenzoquinone. Its battery performance is compared with other quinone-based materials. The authors claim numerous records for their material. However, regardless of capacity, no clear plateaus are visible in the voltage profiles, and the contribution of conductive carbon has not been evaluated. The voltage range is 1–3 V, which is relatively low for a cathode material, so the claim to outcompete LIB cathodes is not appropriate. The characterization of this material and the description of the synthesis are insufficient. The condensation between rhodizonic acid and tetraaminobenzoquinone could produce a range of possible products. Only a single run of elemental analysis is reported. The other analytical techniques described are not specific enough to clearly identify the structure of the material obtained. Was it obtained as a hydrate? Such compounds (including rhodizonate salts and the intermediate product TABQ during synthesis) tend to form hydrates with water. The authors should describe several repeated runs of the elemental analysis. Was the synthesis optimized in any way? This should be discussed in the manuscript. In addition, results on only one cell are reported in each of the half-cell cycle tests. The authors should report the results for at least three different cells with their average values for all measurements performed. No CVs are reported in the electrochemical characterization. Nothing is said about the choice of conductive carbon (which one was used?) and whether there was any optimization with regard to the choice of a specific carbon, which I reckon plays an important role with such an electrode material with a large π-system. The same applies to the electrolyte used. In summary, I cannot recommend the publication of this manuscript in this journal, nor in its current form in any other journal.

Reviewer #2

(Remarks to the Author)
In this manuscript, Huiling Peng et al. systematically investigated a high-performance organic cathode material for lithium-ion batteries. This work demonstrated the superiority of HPQB from multiple aspects, including structural design, stability, electrochemical performance, and charge-discharge mechanisms. The layout of the manuscript is well-organized, and the results obtained are highly encouraging. However, some minor revisions still need to be complemented. Detailed comments are listed as below:

1. Introduction: Some conclusive statements of this study are suitable for the results and discussion section. It is recommended to appropriately simplify the writing of the final paragraph.
2. Please provide the calculation formulas for theoretical specific capacity and gravimetric energy density.
3. Although SEM and TEM images of HPBQ are presented, it is necessary to discuss the intermolecular interactions and stacking distribution at the molecular levels, as which directly impacts the understanding of micropore dimensions.
4. Fig. 6 DFT calculations on PBQ: Please provide the calculation formulas for theoretical voltages along with the structural and energy information involved.
5. Computational Method: Please supplement the references on software and functional methods appropriately.

Reviewer #3

(Remarks to the Author)

This manuscript reports a macrocyclic molecule as a cathode in lithium-ion batteries, showing a high capacity, ultrahigh energy density, and long cycling stability. The molecule or strategy design in the cathode is new, and a relatively thorough mechanism has been developed in experimental and computational studies. Several points need to be cleared before consideration of publication.

Major comments:

1. COFs are known to be AA, AB, or ABC stacked. What are the stacking or aggregation modes of such mscrocycles? This fundamental study has to be done to understand why such macrocycles are working as good cathdoe materials.
2. HPBQ's dispersibility should be studied. Otherwise, it is not convincing that it can work to host Li ions.
3. What are the stable voltage ranges of the HPBQ?
4. High cathode loading of > 10 mg cm– 2 should be tested to show practicality.
5. A pouch cell should be made and tested.
6. Discharge and charge involving 36 and 48 electrons should be quantitatively studied and analyzed.
7. Would there be an effect of the ring sizes of SMOCCs?
8. The major issue is that the author noted the characterization of XRD, NMR, etc. There are no such figures in the main text or SI. Especially, although the monomer synthesis details have been reported somewhat, the characterization of NMR spectra and MS of the molecules is lacking, so it is hard to prove that the authors have synthesized the molecule successfully. Thus, several experiments or data are required to add to the manuscript. This relates to the manuscript's ethics, integrity, and completeness.
9. What would the XRD pattern of HPBQ look like, is it possible to fit with the simulated XRD in HPBQ? The authors already mentioned the method in the characterization part, while the XRD spectra didn't show up in the manuscript.
10. In Fig. 4a and Fig. S8, showing that HPBQ has a poor solubility in organic solvents, then how could it go across the LC-MS to obtain the MS data (e.g., Fig. 2d), or please provide which solvent is used in LC-MS.
11. Please also provide the XRD and TEM of BQ-COF, and what may be the reason for the BET surface area of BQ-COF (38.4 m2 g-1), even lower than the HPBQ (70.56 m2 g-1)?
12. Supplementary molecular dynamics simulations and analyses are recommended, which are essential to support the ion storage mechanism further.
13. The SEM images of as prepared cathodes are recommended to study to show its morphology. Furthermore, it is suggested to provide the different cathodes' SEM images before and after cycling to check if any differences could be observed.
14. Several recent and relevant studies on macrocyclic molecule background and materials/applications reported in papers are missing. The authors are encouraged to review and incorporate citations of the reported macrocyclic molecules to highlight their advantage among COFs or small molecules in general to match with Fig. 1a. For example, (Nat Commun 2021, 12, 2386; Angew. Chem. Int. Ed. 2023, 62, e202300892.; Molecules, 2024, 29.11: 2522.). Furthermore, is there any reported detailed mechanism to analyze the formation of macrocyclic molecules?

Minor comments:

1. Would this system be suitable for other ion batteries? (e.g. Na, Ca, etc.)
2. Provide a detailed calculation with the formula of the theoretical specific capacity of the cathode in the SI for credibility.
3. The authors introduced the Differential capacity (dQ/dV) profiles (Fig. S14) to probe the redox peaks, while they are similar but not totally the same as Cyclic voltammetry (CV) measurements, it is recommended to add the description in the Electrochemical Measurements part on Page 19.

Reviewer #4

(Remarks to the Author)

This manuscript from Zhu and co-workers describes a novel strategy, macrocyclization that synergizes COF-like robustness and small-molecule-like redox efficiency, and construct a macrocyclic organic cathode material, HPBQ, for efficient Li+ charge storage. Given the current huge pursuit of sustainable energy storage solutions, organic electrode materials, which can be sourced from earth abundant elements, have attracted a lot of attention. This work is addressing one of the long-standing problems of organic electrode materials—how to increase the active site utilization while maintaining low solubility—using the newly developed macrocyclization strategy. The reviewer believes that this work meet well the high standard of Nature Communications and will be attractive to the broad readership of this journal. However, there are still some minor concerns that need to be addressed before this manuscript can be accepted.

1. Since the electrodes were discharged all the way to 1 V vs Li+/Li, it's necessary to measure the capacity contribution of conductive carbon to the overall capacity of electrode. Please also specify the type of conductive carbon used in this work because different conductive carbon will have quite different capacity.
2. O1s XPS spectrum shown in Fig. S3 needs to have a better fitting background.
3. For the solid-state 13C NMR spectrum of HPBQ, it is quite confusing that peak 1 and 2 correspond to the two carbonyl groups in the same benzoquinone ring, which should have very similar chemical shifts, especially given the peak broadening observed in the NMR spectrum. Although solid-state 13C NMR is not quantitative, the peak area can still roughly reflect the amount of different C atoms. Then the current assignment cannot explain why peak 2 has much lower intensity and peak area than peak 1, which in fact indicates that the peak 2 C atoms are much less than peak 1 C atoms. And it's also questionable to assign the peak around 130 ppm to three carbons. Along with the mass spec results where there are multiple signals that have lower m/z values, the reviewer suspects that the OH groups in HPBQ have been partially oxidized to o-benzoquinone moieties, which lead to the presence of peak 2.
4. Based on the N2 isotherm, it's not appropriate to assign it as type I because in the current case, most of the porosity is macroscopic porosity. The microscopic porosity only has small contribution.
5. Please provide the ratios between different elements according to the elemental mapping results.
6. The reviewer is curious about the solid-state packing of HPBQ. How do these macrocycles arrange in the solid-state?

Please add some experimental evidence and discussions.
7. The I-V curve shown in the SI (Fig. S7) only includes the 1 V to 2 V region, which is not a typical way to show I-V curves for DC conductivity measurements. The authors should show the I-V curves from negative voltage to positive voltage, e.g., -2 V to 2 V, and use the linear region (must include the origin of the axis when doing the linear fitting) to get the slope. In addition, the reviewer suggests having a more detailed discussion about the comparison between the conductivity of HPBQ and other SMOCCs. In the reference 33, there are quite a few SMOCCs exhibit conductivity higher than 10^-7 S/cm.
8. Based on the proposed charge storage mechanism, the oxidation of hydroxyl to carbonyl groups during the first charging process should result in much higher capacity (48 e, 812 mAh/g) rather than 615 mAh/g. The fact that the second charging process result in ~800 mAh/g suggests that the irreversible conversion happens at the second charging process rather than the first. Is it possible that only deprotonation happens in the first charging process? Please discuss this issue and provide a better explanation (or better charge storage mechanism). Please also provide discussion about the generation of H2O during this irreversible conversion process since it's well-known that H2O is bad for LIBs.
9. The reviewer appreciates the near quantitative active-site utilization realized by the macrocyclization strategy. The reviewer would like to mention that there are several additional SMOCCs with nearly 100% active site utilization, e.g., 1) quinone-fused aza-phenazines (QAPs)-3, 99.5% during the initial discharge (ACS Energy Lett. 2021, 6, 2, 643–649); 2) TAQ, 100% during the initial discharge (ACS Cent. Sci. 2024, 10, 3, 569–578); 3) DPZPC, 96.3% (Chemical Engineering Journal, 2022, 450, 137920.), etc
10. Please provide some discussions about the reason why the Coulombic Efficiency of HPBQ is not close to 100% (looks like around 95%) during the initial 40 cycles or so. Does this relate to the strange fluctuations of the discharge capacity in the same range? Similarly, after the high rate cycling at 5 A/g and reverse back to 0.1 A/g, the CE is not that satisfactory, which requires some discussions and explanations.
11. The reviewer is curious about how carbon content (e.g.., using lower amount of carbon like 20 wt.%) and areal mass loading of active materials (e.g., increasing from 1.5 mg/cm2 to 2 or 3 mg/cm2) affect the performance of HPBQ.
12. It would be great if the authors can provide longer cycling data at 5 A/g since 500 cycles at this rate do not correspond to a long cycle life. 1000 or even 2000 cycles would be great.
13. The reviewer really appreciates the detailed in-situ characterizations of electrode solubility. It would be even nicer to get some quantitative insights into the values of solubility.
14. For Fig. 5b, The reviewer suggests that it would be clearer to also have the spectra curves shown together with the contour map because with the contour map only, sometimes could be kind of misleading or confusing.
15. The reviewer suggests having some discussions about the atomic ratio between Li, N, and O of discharged HPBQ according to XPS results. Do these values match with the proposed mechanism? If possible, it would be great to measure the ex-situ XPS at different state of charge to corroborate the intermediate stages proposed by calculation.

Finally, there are some typos:
1) In the method part, electrochemical measurements, I guess it should be 2032-type coin cell, not 2023.
2) Line 105-106, should be Fourier transform infrared (FTIR) spectroscopy, and "spectrometer" should be removed.

Version 1:

Reviewer comments:

Reviewer #1

(Remarks to the Author)
The authors thoroughly revised the manuscript, which is now much improved and can be accepted without further revision.

Reviewer #2

(Remarks to the Author)
In response to the comments and suggestions I raised, the authors have made meticulous and professional revisions to the manuscript. I am satisfied with the authors' revisions and agree to accept the manuscript for publication. In its current state, I have no further comments or suggestions regarding this manuscript.

Reviewer #3

(Remarks to the Author)
Comments are addressed.

Reviewer #4

(Remarks to the Author)
The additional evidence and results have addressed all my concerns, and I therefore recommend to publish this manuscript in its current form without further change.

Open Access This Peer Review File is licensed under a Creative Commons Attribution 4.0 International License, which permits use, sharing, adaptation, distribution and reproduction in any medium or format, as long as you give appropriate credit to the original author(s) and the source, provide a link to the Creative Commons license, and indicate if changes were made.
In cases where reviewers are anonymous, credit should be given to 'Anonymous Referee' and the source.
The images or other third party material in this Peer Review File are included in the article's Creative Commons license, unless indicated otherwise in a credit line to the material. If material is not included in the article's Creative Commons license and your intended use is not permitted by statutory regulation or exceeds the permitted use, you will need to obtain permission directly from the copyright holder.
To view a copy of this license, visit https://creativecommons.org/licenses/by/4.0/
